# Experimental short-term heatwaves negatively impact body weight gain and survival during larval development in *Bombus terrestris* L. (Hymenoptera: Apidae)

**DOI:** 10.1242/bio.061781

**Published:** 2025-04-25

**Authors:** Laura Wögler, Christoph Kurze

**Affiliations:** Institute for Zoology and Evolutionary Biology, Faculty of Biology and Preclinical Medicine, Universitätsstraße 31, 93053 Regensburg, Germany

**Keywords:** Social insect, Extreme heat, Acute stress, Critical weight, Metamorphosis, Brood, *Bombus terrestris*

## Abstract

Climate change-induced heatwaves threaten global biodiversity, including crucial pollinators like bumblebees. In particular, the increasing frequency, duration and intensity of heatwaves is alarming. Despite these projections, little is known about the effects of short-term heatwaves on insect larval development. Hence, we investigated the impact of simulated heatwaves on the development of 4th instar larvae (L4) of *Bombus terrestris* L. (Hymenoptera: Apidae) using an *in vitro* rearing method. Individual larvae were incubated at 37°C and 38°C for a period of 4 days, with a constant rearing temperature of 34°C as the control. We examined body weight gain, developmental duration, survival to adult stage, and adult body size (i.e. dry mass, intertegular distance, and head width). A simulated heatwave of 37°C did not significantly affect larval development, but 38°C impaired larval body mass gain. While developmental duration and adult body size were unaffected, an acute heat stress of 38°C during the L4 stage reduced the probability of pupae reaching adulthood. These findings highlight the potential for heatwaves to negatively affect bee populations by impairing larval growth and reducing survival to the adult stage, which may have severe implications for colony fitness.

## Teaser

**Summary:** Extreme heatwaves impair bumblebee larval growth and survival, revealing climate change impacts on temperate insect development.

## INTRODUCTION

Climate change is a major challenge of the 21st century, causing cascading effects that impact weather patterns, biodiversity, and entire ecosystems (Garcia et al., 2014; IPCC, 2023). Particularly concerning are heatwaves, defined as at least three consecutive days with extreme heat, which have increased in frequency and intensity (Lhotka et al., 2018; Perkins-Kirkpatrick and Lewis, 2020; Stillman, 2019). These heatwaves have a significant impact on terrestrial animals, including humans (Stillman, 2019), small mammals (Fuller et al., 2021; Ratnayake et al., 2019; Zhao et al., 2020), birds (Conradie et al., 2019; McKechnie and Wolf, 2010), and insects (Bodlah et al., 2023; González-Tokman et al., 2020; Ma et al., 2021). Despite this threat, our understanding of the impact of heatwaves on animal development, their fitness, and populations remains limited (Fuller et al., 2021; González-Tokman et al., 2020; Jentsch et al., 2007; Ma et al., 2021; Stillman, 2019).

Cold-adapted heterothermic species like bumblebees (*Bombus* sp.), which have species-specific distributions ranging from the Arctic to temperate/Mediterranean regions, may be particularly at risk (Ghisbain et al., 2024; Maebe et al., 2021; Martinet et al., 2021a; Rasmont and Iserbyt, 2012; Soroye et al., 2020; Suzuki-Ohno et al., 2020). As key pollinators in many ecosystems and for agriculture (Cameron and Sadd, 2020; Corbet et al., 1991; Klein et al., 2007; Potts et al., 2016), understanding how extreme heat events impact their physiology and fitness is crucial. Although heat tolerance varies greatly between species, with alpine and polar species being particularly sensitive to heat stress (Martinet et al., 2021a; Zambra et al., 2020), commercially used bumblebees such as *Bombus terrestris* are typically housed aboveground, making them more frequently exposed to extreme heat. Recent studies show that heat stress can reduce adult survival (Kuo et al., 2023; Quinlan et al., 2023) and elevated temperatures may lower colony fitness (Martinet et al., 2021b; Theodorou et al., 2022). Under heatwave-like temperatures, adult bumblebees exhibit impaired cognition (Gérard et al., 2022a) and scent perception (Nooten et al., 2024), and display altered fanning and foraging behaviours (Bretzlaff et al., 2024; Kuo et al., 2023; Sepúlveda et al., 2024), potentially affecting colony fitness. Interestingly, foraging behaviour and response to stimuli are altered in adults even when exposed to heatwave-like temperatures during their larval and pupal development (Gérard et al., 2022b; Perl et al., 2022). Exposing entire colonies to high temperatures for extended periods can lead to alterations in wing size asymmetry, wing shape and size, and reductions in body and antennae sizes (Guiraud et al., 2021; Gérard et al., 2022b, 2023, 2018; Perl et al., 2022). Despite that different developmental stages are likely to be variably affected, it is unclear which stages are particularly vulnerable to heatwaves and whether shorter extreme heat events are sufficient to impair their development.

To address this knowledge gap, we adapted an *in vitro* rearing protocol (Kato et al., 2022) to examine the direct impact of a 4-day-long heat stress period during the development of 4th instar larvae (L4). This experimental approach prevented heat mitigation through worker fanning activities (Weidenmüller et al., 2002). While *in vitro* rearing is a standard procedure in honeybee research (Crailsheim et al., 2013; Schmehl et al., 2016), it is rarely used in bumblebee research (Kato et al., 2022; Pereboom et al., 2003). Therefore, we chose *B. terrestris* as a model species, although it is known to be rather heat-tolerant (Martinet et al., 2021a; Zambra et al., 2020). We used this method to investigate changes in body mass, developmental duration, survival until pupation and emergence, and adult body size following a 4-day-long heatwave-like exposure during L4 development. Additionally, we assessed lipid content; as the fat body in adult bees is linked to reproductive success, immunity, stress resilience, and foraging efficiency, making it a potentially valuable marker for monitoring bee health (Vanderplanck et al., 2021).

## RESULTS

**Fig. 1.**
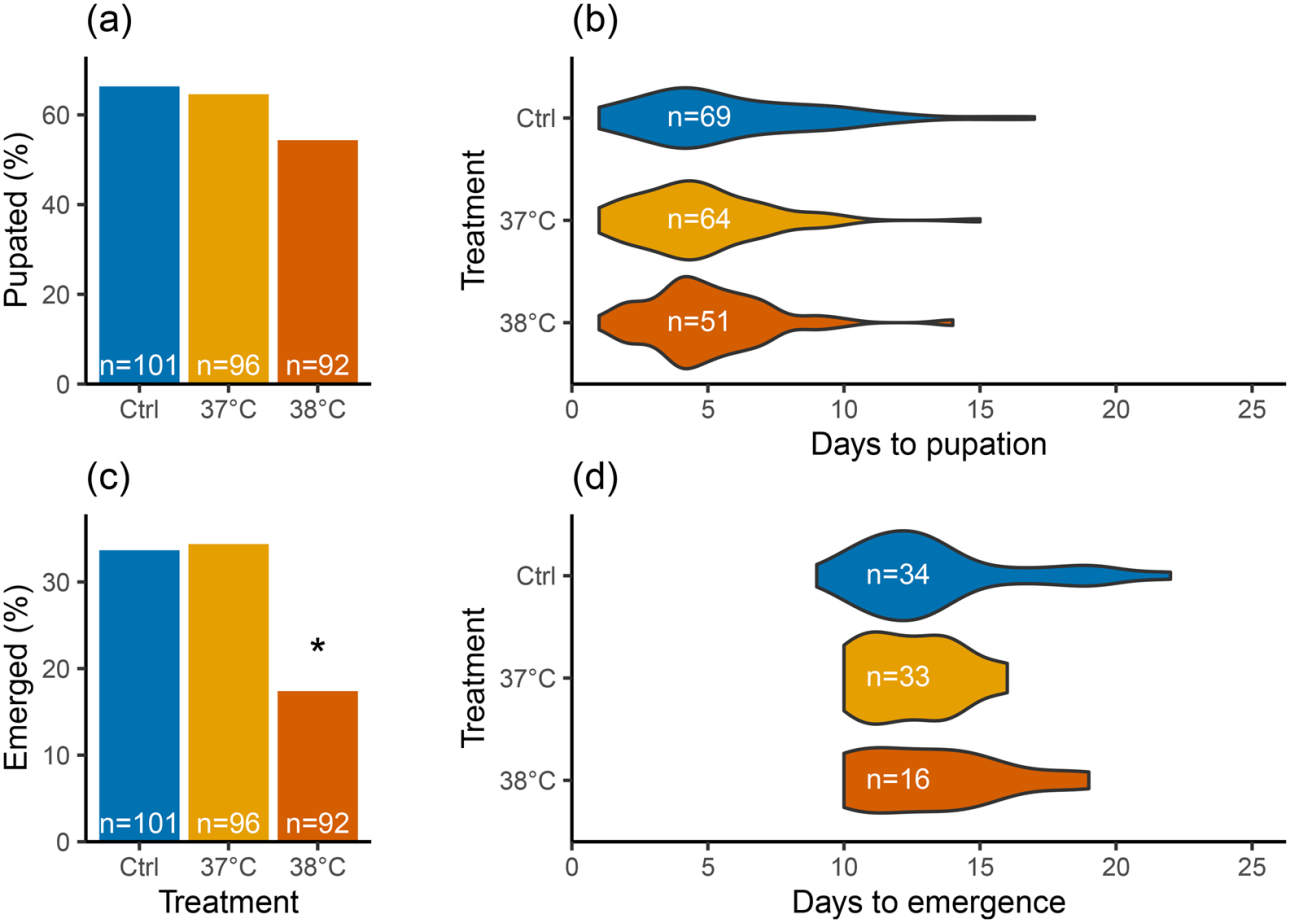
**Effects of 4-day-long simulated heatwaves on survival and developmental duration of L4 larvae (A,C) and pupae (C,D).** The controls (Ctrl, reared at constant 34°C, in blue) were compared to individuals that had been exposed to increased rearing temperatures at 37°C (in warm yellow) and 38°C (in reddish-orange) during L4 development for 4 days. Significant differences (*P*<0.05) are denoted with an asterisk (*).

A 4-day-long heatwave at the beginning of L4 development significantly impacted *B. terrestris*' probability of reaching adulthood (*χ*^2^=6.48, d.f.=2, *P*=0.039; Fig. 1C). Larvae exposed to 38°C were 50% less likely to reach adulthood compared to larvae exposed to 37°C (*P*=0.024) or the control group (*P*=0.024). However, there was no significant difference in survival between the 37°C group and the control group (*P*=1). The number of L4 larvae reaching the pupal stage was not significantly affected (*χ*^2^=1.36, d.f.=2, *P*=0.507; Fig. 1A). There was also no significant effect of heatwave treatment on the developmental times for larvae reaching pupal stage [generalized linear mixed effect model (GLMM): *χ*^2^=2.50, d.f.=2, *P*=0.287; Fig. 1B] and for pupae until emergence (GLMM: *χ*^2^=0.54, d.f.=2, *P*=0.762; Fig. 1D).

**Fig. 2.**
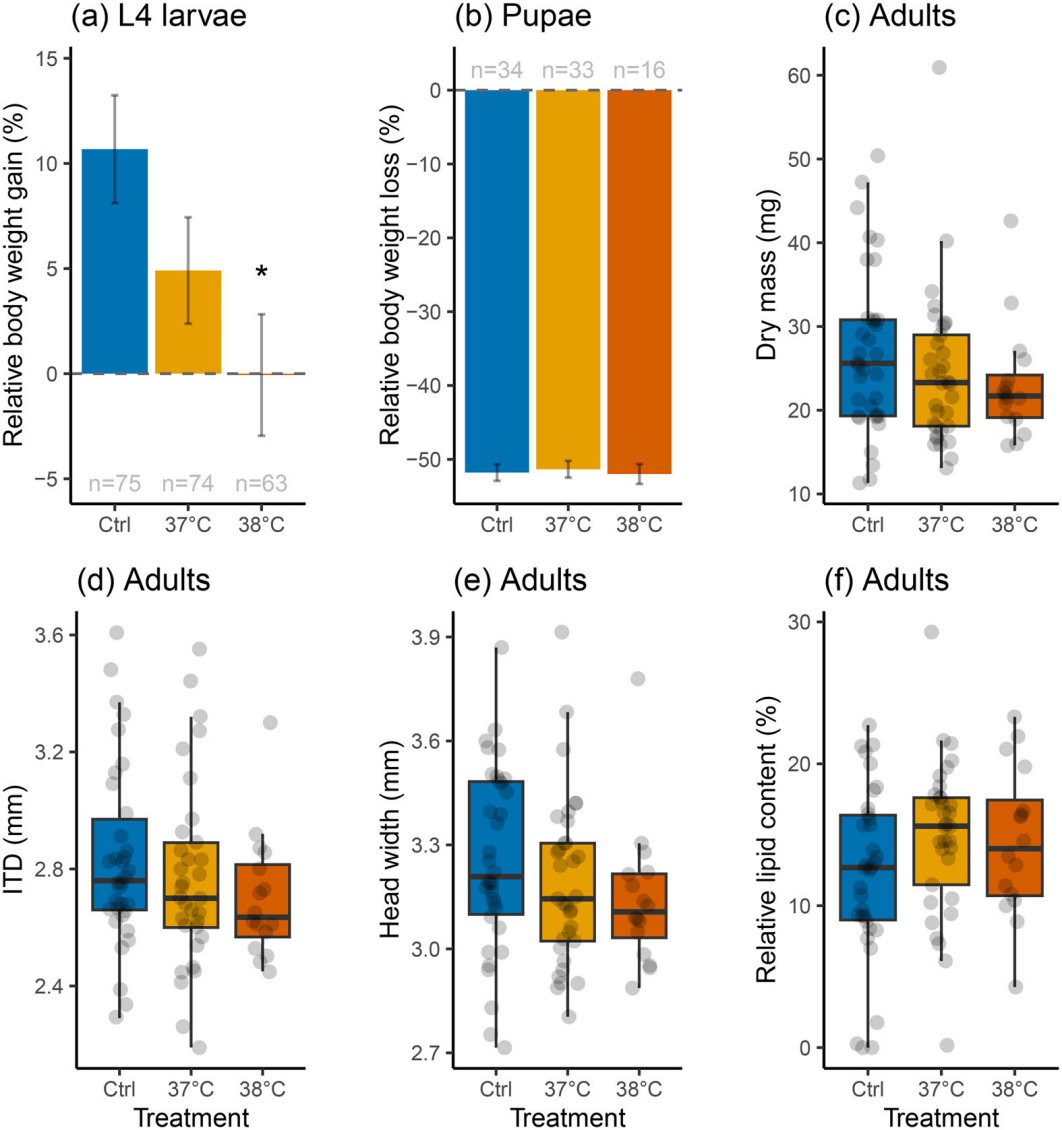
**Effects of 4-day-long simulated heatwaves during L4 stage on bumblebee morphometrics.** (A) Relative body mass gain during treatment (means±s.e.). (B) Relative body mass loss during pupation (means±s.e.). (C-F) Consequences on their dry mass, ITD (intertegular distance), head width, and relative lipid content as 2-day-old adults. The controls (Ctrl, reared at constant 34°C, in blue) were compared to individuals that had been exposed to increased rearing temperatures at 37°C (in warm yellow) and 38°C (in reddish-orange) during L4 development for 4 days. Significant differences (*P*<0.05) are denoted with an asterisk (*).

Heatwaves significantly affected the relative weight gain in L4 larvae during the treatment (glmm: *χ*^2^=7.94, d.f.=2, *P*=0.019; Fig. 2A). While exposure to 38°C revealed significantly lower weight gains compared to the control (Tukey HSD: t-ratio=2.80, *P*=0.015), there was no significant effect for larvae exposed to 37°C (t-ratio=1.61, *P*=0.242). Regardless of treatment, their body weight loss during pupation had no significant effect (*χ*^2^=0.60, d.f.=2, *P*=0.741; Fig. 2B). There were also no significant effects on morphometrics in 2-day-old adults, including their dry mass (*χ*^2^=2.26, d.f.=2, *P*=0.322; Fig. 2C), ITD (*χ*^2^=2.58, d.f.=2, *P*=0.275; Fig. 2D), head width (*χ*^2^=2.25, d.f.=2, *P*=0.325; Fig. 2E), and relative lipid content (*χ*^2^=4.07, d.f.=2, *P*=0.131; Fig. 2F). However, it is worth noting that the sample size was significantly reduced in the 38°C treatment group at the adult stage (*n*=16, see Fig. 1C,D).

We found that both the heatwave treatment and relative body weight gain during treatment impacted the probability of larvae reaching adulthood (treatment: *χ*^2^=14.759, d.f.=2, *P*=0.0006; weight gain: *χ*^2^=24.35, d.f.=1, *P*<0.0001), but not their interactions (*χ*^2^=0.04, d.f.=2, *P*=0.980). In addition, we found that relative body weight gain had a significant effect on the probability of larvae to pupate (*χ*^2^=14.10, d.f.=1, *P*=0.0002).

## DISCUSSION

Our data provide evidence of how simulated short-term heatwaves during the L4 stage affect development and survival until adulthood in *B. terrestris* (Figs 1A and 2A). Nonetheless, there was no effect on the duration of larval development (Fig. 1B,D), nor on the morphometrics in adults (Fig. 2C-E). This contrasts with previous studies showing that exposing colonies to elevated temperatures produced smaller workers, as indicated by smaller ITD (Guiraud et al., 2021; Gérard et al., 2023), with reduced antennae in *B. terrestris* (Gérard et al., 2023). In addition, while wing size and shape can be affected (Gérard et al., 2018), this may not always be the case (Gérard et al., 2023). As only 17% of individuals emerged as adults in the 38°C treatment group of our experiment, this sample size was too small to thoroughly analyse wing morphology. Regardless, a potential explanation for this discrepancy could be that colonies were exposed to higher temperatures for extended periods in those experiments, whereas we tested the effect of short-term heatwave-like exposures in L4 larvae. This explanation is supported by another study showing that body or organ sizes were also not altered when colonies were exposed to elevated temperatures for shorter periods (Perl et al., 2022).

We found there was a 50% lower probability that L4 larvae exposed to 38°C would emerge as adults compared to both the 37°C heatwave group and the control group (Fig. 1C). Although the emergence rate in our *in vitro* rearing was low, our pupation rate, ranging between 55-66% irrespective of treatment, was similar to previous research (Kato et al., 2022). This suggests that 38°C might be a threshold temperature with ripple effects on critical processes during pupation. While insects typically exhibit left-skewed temperature responses, characterized by a steep decline in fitness beyond the temperature optimum, this pattern is generally more pronounced in tropical- rather than cold-adapted insects (Deutsch et al., 2008).

The evolution of complex thermoregulatory behaviours, including fanning, metabolic heat generation, and direct incubation of brood, likely resulted in a narrower optimal temperature range in bumblebee larvae, similar to that of honeybees (Jones and Oldroyd, 2006). Nonetheless, nest thermoregulation comes with increased energetic costs (Bretzlaff et al., 2023), which likely lead to reduced colony growth (Theodorou et al., 2022; Vogt, 1986). Above a certain temperature threshold, or when colonies are not large enough, maintaining nest thermoregulation may become unsustainable. For example, in *B. impatiens*, offspring production decreases at 35°C as workers abandon their colony (Bretzlaff et al., 2024). In *B. terrestris*, this threshold appears to be higher, with drone production increasing with elevated nest temperatures up to 34-36°C in microcolonies, at which point workers massively increase fanning activity (Sepúlveda et al., 2024). Besides differences between bumblebee species and populations (Zambra et al., 2020), resilience to heat stress likely also differs between aboveground and belowground nesting species (Gonzalez et al., 2024). To limit confounding effects, such as worker behaviour, food supply and quality, we removed larvae from their natural nest environment and reared them *in vitro* under highly controlled conditions. While our experiment approach does not reflect a realistic scenario, *in vitro* rearing allowed us to control the timing and duration of heatwaves. Furthermore, it allowed us to closely monitor weight gain and loss throughout larval development until emergence (Fig. 2A,B).

A 4-day-long exposure to 38°C during L4 development resulted in lower weight gain compared to both the 37°C heatwave and control groups (Fig. 2A). This suggests that larvae either consumed less food or experienced increased energetic costs that could not be compensated by food intake. While it is known that stress reduces food intake and consequently weight gain in mammals (Rabasa and Dickson, 2016), surprisingly little is known about how acute stress affects food consumption and body weight gain in insects. Reaching a critical weight during the larval development, however, is crucial for initiating molting and metamorphosis, as shown in the tobacco hornworm (*Manduca sexta* L., Lepidoptera: Sphingidae) (Nijhout and Williams, 1974). It has also been shown that reaching a critical weight can be influenced by both temperature and food quality (Davidowitz et al., 2003). Our data confirms this for bumblebees, with weight gain at the L4 stage being a significant predictor of pupation success, irrespective of the treatment group.

In 3rd instar fruit fly larvae (*Drosophila melanogaster* M., Diptera: Drosophilidae) short-term heat stress reduced food intake in adults on the day after emergence, without impacting their body weight but leading to increased glucose and trehalose levels while reducing lipid stores (Karpova et al., 2024). Although we did not detect any significant difference in body size (i.e. dry mass, ITD, and head width) or relative lipid content in adult bees between treatment groups, we observed a notable decrease in body size variance with increasing heat stress (Fig. 2C-F). One potential explanation is that our extreme heat stress may have selected for individuals with adaptive traits, resulting in a more homogenous size distribution among the surviving bees. Another possibility is that the massive weight loss during metamorphosis may mask more subtle effects on adult body size. A third explanation could be the reduced sample size, particularly in the 38°C heatwave group, and the slightly higher number of males in the control group.

Although both the heatwave treatment and the weight gain had a significant effect on the probability of larvae reaching adulthood, there was no interaction between both factors. This suggests that weight gain similarly increased survival, regardless of the treatment group. Nevertheless, our data supports the hypothesis that acute stress during larval development has a drastic impact at a later life stage (Karpova et al., 2024), although effects could have also just been delayed in our experiment. An exposure to 38°C at the L4 stage marked a threshold at which processes during metamorphosis are likely impaired in *B. terrestris*. It would be interesting to see how acute stress in early development would impact the life-history of adult bees. It has already been shown that heatwave-like temperatures during late development impact initial behavioural responses to sensory stimuli in adult workers of *B. terrestris* (Perl et al., 2022). This could not only have detrimental effects for the individual worker but also ripple effects on colony fitness.

In conclusion, while our *in-vitro* rearing experiment showed a certain resilience of *B. terrestris* larvae to heatwave-like exposures up to 37°C, extreme temperatures of 38°C appeared to be the threshold where pupal development was severely impaired. Individuals reaching adulthood, however, did not differ in their body size (i.e. dry mass, ITD, and head width) and relative lipid content, suggesting potential adaptive advantages in those surviving bees. With our experimental approach, we aimed to investigate the specific effects of acute thermal stress at the L4 stage, which we traded for experimental realism. Larvae were taken out of their natural nest environment, which likely has a large impact on their survival. Although our selected temperatures may appear extreme, they are only 3°C and 4°C above what are considered to be optimal rearing conditions. Our heatwave scenarios closely reflect realistic conditions in commercially used bumblebees, which are typically housed aboveground. Since we studied the relatively heat-tolerant *B. terrestris* as a model species, we would expect more severe effects in cold-adapted species, such as *B. lapidarius*, *B. alpinus* or *B. poralris* (Martinet et al., 2021a). Additionally, the impact of heatwave could also be more pronounced under natural conditions, where colonies are exposed to multiple stressors simultaneously (Theodorou et al., 2022). Given the increasing frequency and severity of heatwaves, it is crucial to investigate their impact on the life-history and adaptive potential of keystone species like bumblebees.

## MATERIALS AND METHODS

### Experimental overview

To simulate the effect of short-term heatwaves on larval development, we collected L4 larvae (a total of 289) from five commercial *B. terrestris* colonies to rear them *in vitro*. These larvae were pseudo-randomly assigned to one of the three experimental groups to ensure equal distribution among treatments and colonies. Larvae of the simulated heatwave treatment were exposed to either 37°C (*n*=96) or 38°C (*n*=92) for 4 days, while the control group (ctrl, *n*=101) was reared at a constant 34°C. The control temperature was chosen based on previous research (Kato et al., 2022), although it is at the upper range of the typical brood temperatures reported in earlier studies (Vogt, 1986; Weidenmüller et al., 2002). Our treatment temperatures aligned with our separate study on pupal development (Laußer and Kurze, 2025 preprint), aimed to simulate potential extreme heatwave scenarios in Europe. For example, heatwaves reaching or exceeding temperatures of 38°C have occurred in parts of the British Isles, Mediterranean and Eastern Europe in recent years (Barriopedro et al., 2011; Lhotka and Kyselý, 2024; Rita et al., 2020). Moreover, our treatments are still comparable to previous studies, considering that the actual brood temperature is typically 2°C warmer than the ambient nest temperature (Vogt, 1986; Weidenmüller et al., 2002). At these elevated treatment temperatures, *B. terrestris* workers spend significantly more time cooling the brood through fanning (Sepúlveda et al., 2024), suggesting that such temperatures could have adverse effects on larval development. We recorded body mass changes during treatment and pupal stage. Their survival was checked daily until adult bees emerged and reached the age of 2 days. These adult bees were freeze-killed and kept at −20°C for subsequent morphometric measurements and analysis of their dry mass and lipid content.

### Colony husbandry

Upon arrival, each *B. terrestris* colony (Natupol Research Hives, Koppert B.V., Netherlands) consisted of 20-30 workers, brood, and one queen. They were housed and maintained under similar conditions as described previously (Gilgenreiner and Kurze, 2024). Briefly, bumblebees had access to 70% (w/v) sucrose solution *ad libitum* in a foraging arena [59 (l)×39 (w)×26 (h) cm] with 14:10 h light:dark regime. Depending on colony size, each colony received 6-11 g of pollen candy daily, consisting of 67% organic pollen (naturwaren-niederrhein GmbH, Germany), 25% sucrose and 8% tap water. The room temperature was maintained at 25°C±1°C and 30-50% relative humidity. We allowed all colonies to develop for at least 1 month before starting to collect L4 larvae for the experiment.

### Collection of 4th instar larvae

In this study, we exclusively focused on 4th instar larvae because they are easily identifiable inside the nest by their individual, spherical brood cells with a small opening for food provisioning (Tian and Hines, 2018). Additionally, our pilot study showed that they can be reared *in vitro* more successfully than earlier larval stages without unacceptably high mortality rates. Before collecting L4 larvae for our experiment, we carefully removed all existing L4 larvae from each colony using soft tweezers. This allowed us to identify and collect larvae that just had entered the L4 stage during our daily colonies checks over the following days. To facilitate the collection process, we temporarily moved all adult bumblebees to a separate cage and returned them afterwards.

### *In vitro* rearing

**Fig. 3.**
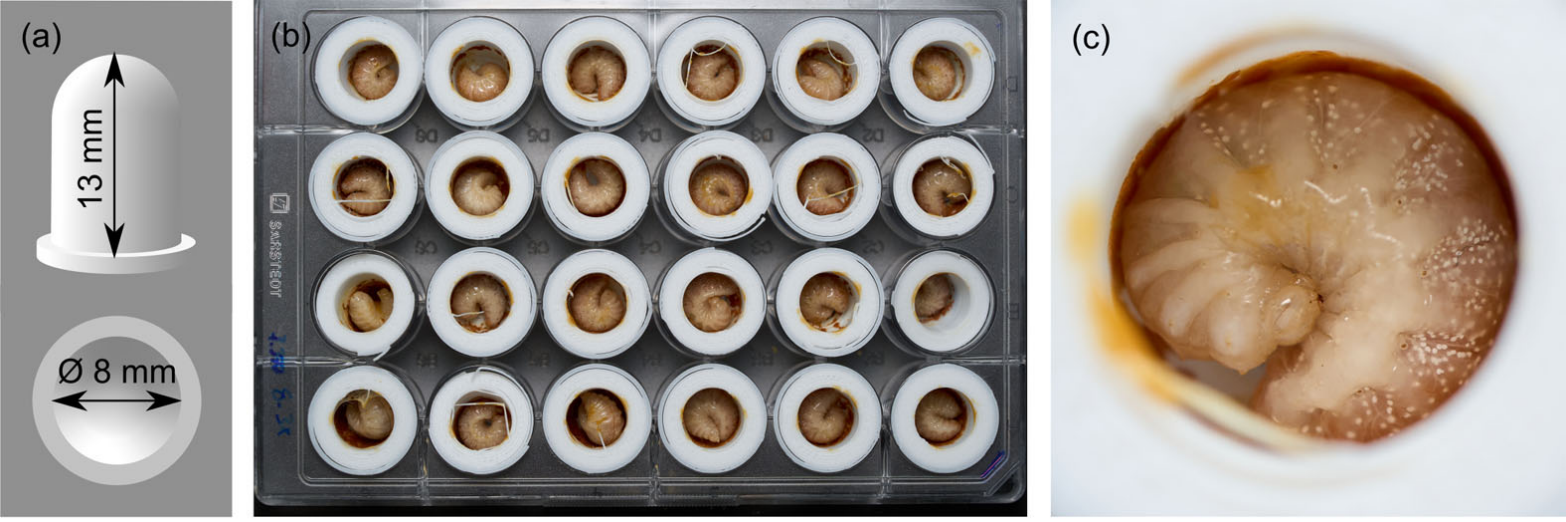
***In vitro* rearing of L4 larvae.** (A) Design of 3D-printed polylactide (PLA) artificial brood cells (capacity: 0.6 ml). (B) Artificial brood cells with L4 larvae inside a 24-well clear flat bottom plate, and (C) L4 feeding on medium.

We followed established *in vitro* rearing procedures with slight modifications (Crailsheim et al., 2013; Kato et al., 2022), where we carefully transferred L4 larvae into 3D-printed polylactide (PLA) artificial brood cells (capacity=0.6 ml, diameter=8 mm, Fig. 3A). This facilitated measuring their weight gain without touching them again. To simulate short-term heatwaves, we randomly assigned L4 larvae to one of the three experimental groups and either reared them at 37°C and 38°C for 4 days (KB115, BINDER GmbH, Germany). The control group (ctrl) was maintained at constant temperature of 34°C (Kato et al., 2022; Pereboom et al., 2003). Those artificial brood cells were placed into 24- or 48-well clear flat bottom plates (Falcon/Corning, USA) (Fig. 3B) and kept inside in ventilated plastic containers (18.5×18.5×11.5 cm) together with a 120 ml cup of saturated sodium chloride solution to maintain 65±10% relative humidity.

Larvae were fed with a pollen medium twice daily, in the morning and in the evening. The medium contained 50% w/v sucrose solution (Südzucker AG, Germany), 40% honeybee collected organic pollen (Bio-Blütenpollen, naturwaren-niederrhein GmbH, Germany), 10% Bacto yeast extract (Bacto™, BD, USA), and 1% casein sodium salt from bovine milk (Sigma-Aldrich, Germany). Aliquots of medium were stored at −20°C and warmed up to 34°C and vortexed before feeding. A feeding session consisted of two 20-min rounds on a heated plate at 35°C (Medax model 12801, Medax Nagel GmbH, Germany). Larvae were initially fed a 6 μl droplet (7.1±1.6 mg) of medium onto their ventral abdomen (Fig. 3C). We monitored larval behaviour to determine satiation. A larva was considered satiated when it curled up and ceased movement and hungry when it remained active (Movie 1). Larvae that did not consume food during the first feeding round were not offered additional food. At the end of the feeding session, any remaining food was carefully removed to prevent the larvae from suffocating due to dried food blocking their trachea. Larvae entering pupal stage were no longer fed. Due to the difficulty in accurately monitoring survival during the pupal stage, daily survival data were not included. Instead, we present the proportion of larvae that pupated and successfully reached adulthood.

### Measurements of body mass

Each individual bee was weight at four different stages: as L4 larvae pre- and post-treatment, at the beginning of pupal stage, and as a newly emerged adult using a fine scale (d=0.1 mg, Sartorius AC120S, Sartorius AG, Germany). To reduce stress and avoid any potential handling damage of the larvae, we kept them inside their 3D-printed cell. Consequently, we subtracted the empty cell weight to obtain the actual weights.

### Morphometric measurements, dry mass, and lipid content in adults

The intertegular distance (ITD) and dry mass serve as a proxy for adult body size (Gilgenreiner and Kurze, 2024; Kendall et al., 2019). In addition to the ITD, we measured the head width and determined the sex by counting antennal segments (females have 12, males 13) using a digital microscope (CHX-500F, Keyence GmbH, Germany). In total, 64 females and 19 males emerged as adults. We obtained individual dry mass and lipid content following our previous protocol (Gilgenreiner and Kurze, 2024). Briefly, we dissected the ventral abdominal segments and dried corpses at 60°C for 3 days in a drying cabinet (U40, Memmert GmbH & Co. KG, Germany). After weighing their dry mass (d=0.1 mg, analytic balance M-Pact AX224, Sartorius GmbH, Germany), we extracted their body lipid with petroleum ether for 5 days. After discarding the ether and rinsing them with fresh ether, the bees were dried for an additional 3 days and weighed again. The lipid content was calculated as the difference between the initial and post-extraction dry weights.

### Statistical analyses

All statistical analyses and data visualizations were performed using R Statistical Software (v4.4.1; R Core Team, 2024). The complete code and output are provided in the electronic supplementary information. Briefly, the probability of L4 larvae reaching papal stage and adulthood was calculated using *chisq-test* function to perform a Pearson's *χ*^2^ test for count data with Yate's continuity correction. Pairwise comparisons of survival probabilities between survival treatment groups were conducted using the *pairwise.prop.test* function with Benjamini-Hochberg correction. In addition, we ran GLMMs using the *glmmTMB* package (Brooks et al., 2017) with either gaussian or gamma data distribution to analyse effects of the heatwave treatment as a fixed factor on the duration to pupation and to emergence, the relative body weight gain during treatment, the relative body weight loss during pupation, adult body size (with dry mass, ITD, and head width serving as proxies), and the relative lipid content as response variables. In addition, we ran another GLMM based on binomial data distribution to test whether both heatwave and relative body weight gain as well as their interactions affected their probability to reach pupal stage and adulthood. We included ‘colony ID’ as a random effect in all models to account for colony-specific variability. Additionally, we included ‘sex’ as a random effect in models involving adult body size measurements (dry mass and head width) and relative lipid contents to account for potential morphometric differences between workers and male bumblebees. Model selection was performed based on the Akaike information criterion (AIC) and likelihood ratio tests. The final models were compared with their respective null-models. Model assumptions and dispersion of the data were checked using the *DHARMa* package (Hartig, 2020). Significance levels (*P*<0.05) were determined using the *Anova* function of the *car* package (Zuur et al., 2009). Pairwise comparisons between treatment groups were conducted using the function *emmeans* (Lenth and Lenth, 2018) adjusted with Tukey's HSD.

### Ethics statement

This study was conducted in accordance with the ethical regulations of the German Animal Welfare Act (TierSchG) for conducting experiments with insects.

## Supplementary Material

10.1242/biolopen.061781_sup1 Supplementary information

Table S1.

Table S2.

## References

[BIO061781C1] Barriopedro, D., Fischer, E. M., Luterbacher, J., Trigo, R. M. and García-Herrera, R. (2011). The hot summer of 2010: redrawing the temperature record map of Europe. *Science* 332, 220-224. 10.1126/science.1201224 21415316

[BIO061781C2] Bodlah, M. A., Iqbal, J., Ashiq, A., Bodlah, I., Jiang, S., Mudassir, M. A., Rasheed, M. T. and Fareen, A. G. E. (2023). Insect behavioral restraint and adaptation strategies under heat stress: an inclusive review. *J. Saudi Soc. Agric. Sci.* 22, 327-350. 10.1016/j.jssas.2023.02.004

[BIO061781C3] Bretzlaff, T., Kerr, J. T. and Darveau, C.-A. (2023). High temperature sensitivity of bumblebee castes and the colony-level costs of thermoregulation in *Bombus impatiens*. *J. Therm. Biol.* 117, 103710. 10.1016/j.jtherbio.2023.103710 37716225

[BIO061781C4] Bretzlaff, T., Kerr, J. T. and Darveau, C.-A. (2024). Handling heatwaves: balancing thermoregulation, foraging and bumblebee colony success. *Conserv. Physiol.* 12, coae006. 10.1093/conphys/coae006 38332907 PMC10853005

[BIO061781C5] Brooks, M. E., Kristensen, K., Van Benthem, K. J., Magnusson, A., Berg, C. W., Nielsen, A., Skaug, H. J., Mächler, M. and Bolker, B. M. (2017). glmmTMB balances speed and flexibility among packages for zero-inflated generalized linear mixed modeling. *R Journal* 9, 378-400. 10.32614/RJ-2017-066

[BIO061781C6] Cameron, S. A. and Sadd, B. M. (2020). Global trends in bumble bee health. *Annu. Rev. Entomol.* 65, 209-232. 10.1146/annurev-ento-011118-111847 31610137

[BIO061781C7] Conradie, S. R., Woodborne, S. M., Cunningham, S. J. and McKechnie, A. E. (2019). Chronic, sublethal effects of high temperatures will cause severe declines in southern African arid-zone birds during the 21st century. *Proc. Natl Acad. Sci. USA* 116, 14065-14070. 10.1073/pnas.1821312116 31235571 PMC6628835

[BIO061781C8] Corbet, S. A., Williams, I. H. and Osborne, J. L. (1991). Bees and the pollination of crops and wild flowers in the European Community. *Bee World* 72, 47-59. 10.1080/0005772X.1991.11099079

[BIO061781C9] Crailsheim, K., Brodschneider, R., Aupinel, P., Behrens, D., Genersch, E., Vollmann, J. and Riessberger-Gallé, U. (2013). Standard methods for artificial rearing of *Apis mellifera* larvae. *J. Apic. Res.* 52, 1-16. 10.3896/IBRA.1.52.1.05

[BIO061781C10] Davidowitz, G., D'amico, L. J. and Nijhout, H. F. (2003). Critical weight in the development of insect body size. *Evol. Dev.* 5, 188-197. 10.1046/j.1525-142X.2003.03026.x 12622736

[BIO061781C11] Deutsch, C. A., Tewksbury, J. J., Huey, R. B., Sheldon, K. S., Ghalambor, C. K., Haak, D. C. and Martin, P. R. (2008). Impacts of climate warming on terrestrial ectotherms across latitude. *Proc. Natl Acad. Sci. USA* 105, 6668-6672. 10.1073/pnas.0709472105 18458348 PMC2373333

[BIO061781C12] Fuller, A., Mitchell, D., Maloney, S. K., Hetem, R. S., Fonsêca, V. F., Meyer, L. C., van de Ven, T. M. and Snelling, E. P. (2021). How dryland mammals will respond to climate change: the effects of body size, heat load and a lack of food and water. *J. Exp. Biol.* 224, jeb238113. 10.1242/jeb.238113 33627465

[BIO061781C13] Garcia, R. A., Cabeza, M., Rahbek, C. and Araújo, M. B. (2014). Multiple dimensions of climate change and their implications for biodiversity. *Science* 344, 1247579. 10.1126/science.1247579 24786084

[BIO061781C14] Gérard, M., Michez, D., Debat, V., Fullgrabe, L., Meeus, I., Piot, N., Sculfort, O., Vastrade, M., Smagghe, G. and Vanderplanck, M. (2018). Stressful conditions reveal decrease in size, modification of shape but relatively stable asymmetry in bumblebee wings. *Sci. Rep.* 8, 15169. 10.1038/s41598-018-33429-4 30310103 PMC6181934

[BIO061781C15] Gérard, M., Amiri, A., Cariou, B. and Baird, E. (2022a). Short–term exposure to heatwave-like temperatures affects learning and memory in bumblebees. *Glob. Change Biol.* 28, 4251-4259. 10.1111/gcb.16196 PMC9541601 35429217

[BIO061781C16] Gérard, M., Cariou, B., Henrion, M., Descamps, C. and Baird, E. (2022b). Exposure to elevated temperature during development affects bumblebee foraging behavior. *Behav. Ecol.* 33, 816-824. 10.1093/beheco/arac045 35812365 PMC9262166

[BIO061781C17] Gérard, M., Guiraud, M., Cariou, B., Henrion, M. and Baird, E. (2023). Elevated developmental temperatures impact the size and allometry of morphological traits of the bumblebee Bombus terrestris. *J. Exp. Biol.* 226, jeb245728. 10.1242/jeb.245728 36995273 PMC10263145

[BIO061781C18] Ghisbain, G., Thiery, W., Massonnet, F., Erazo, D., Rasmont, P., Michez, D. and Dellicour, S. (2024). Projected decline in European bumblebee populations in the twenty-first century. *Nature* 628, 337-341. 10.1038/s41586-023-06471-0 37704726

[BIO061781C19] Gilgenreiner, M. and Kurze, C. (2024). Age dominates flight distance and duration, while body size shapes flight speed in *Bombus terrestris* L. (Hymenoptera: Apidae). *Proc. R. Soc. B* 291, 20241001. 10.1098/rspb.2024.1001 PMC11288671 39079662

[BIO061781C20] Gonzalez, V. H., Herbison, N., Robles Perez, G., Panganiban, T., Haefner, L., Tscheulin, T., Petanidou, T. and Hranitz, J. (2024). Bees display limited acclimation capacity for heat tolerance. *Biol. Open* 13, bio060179. 10.1242/bio.060179 38427330 PMC10979511

[BIO061781C21] González-Tokman, D., Córdoba-Aguilar, A., Dáttilo, W., Lira-Noriega, A., Sánchez-Guillén, R. A. and Villalobos, F. (2020). Insect responses to heat: physiological mechanisms, evolution and ecological implications in a warming world. *Biol. Rev.* 95, 802-821. 10.1111/brv.12588 32035015

[BIO061781C22] Guiraud, M., Cariou, B., Henrion, M., Baird, E. and Gérard, M. (2021). Higher developmental temperature increases queen production and decreases worker body size in the bumblebee *Bombus terrestris*. *J. Hymenoptera Res.* 88, 39-49. 10.3897/jhr.88.73532

[BIO061781C23] Hartig, F. (2024). *DHARMa: Residual Diagnostics for Hierarchical (Multi-Level / Mixed) Regression Models*. R package version 0.4.7, https://github.com/florianhartig/dharma

[BIO061781C24] IPCC. (2022). Summary for policymakers. In *Climate Change 2022: Impacts, Adaptation and Vulnerability. Contribution of Working Group II to the Sixth Assessment Report of the Intergovernmental Panel on Climate Change* (ed. H.-O. Pörtner, D. C. Roberts, M. Tignor, E. S. Poloczanska, K. Mintenbeck, A. Alegría, M. Craig, S. Langsdorf, S. Löschke, V. Möller, A. Okem and B. Rama), pp. 3-33. Cambridge, UK and New York, NY, USA: Cambridge University Press. 10.1017/9781009325844.001

[BIO061781C25] Jentsch, A., Kreyling, J. and Beierkuhnlein, C. (2007). A new generation of climate–change experiments: events, not trends. *Front. Ecol. Environ.* 5, 365-374. 10.1890/1540-9295(2007)5[365:ANGOCE]2.0.CO;2

[BIO061781C26] Jones, J. C. and Oldroyd, B. P. (2006). Nest thermoregulation in social insects. *Adv. Insect Physiol.* 33, 153-191. 10.1016/S0065-2806(06)33003-2

[BIO061781C27] Karpova, E. K., Bobrovskikh, M. A., Burdina, E. V., Adonyeva, N. V., Deryuzhenko, M. A., Zakharenko, L. P., Petrovskii, D. V. and Gruntenko, N. E. (2024). Larval stress affects adult Drosophila behavior and metabolism. *J. Insect Physiol.* 159, 104709. 10.1016/j.jinsphys.2024.104709 39299381

[BIO061781C28] Kato, Y., Kikuta, S., Barribeau, S. M. and Inoue, M. N. (2022). In vitro larval rearing method of eusocial bumblebee *Bombus terrestris* for toxicity test. *Sci. Rep.* 12, 15783. 10.1038/s41598-022-19965-0 36138070 PMC9499950

[BIO061781C29] Kendall, L. K., Rader, R., Gagic, V., Cariveau, D. P., Albrecht, M., Baldock, K. C., Freitas, B. M., Hall, M., Holzschuh, A., Molina, F. P. et al. (2019). Pollinator size and its consequences: Robust estimates of body size in pollinating insects. *Ecol. Evol.* 9, 1702-1714. 10.1002/ece3.4835 30847066 PMC6392396

[BIO061781C30] Klein, A.-M., Vaissière, B. E., Cane, J. H., Steffan-Dewenter, I., Cunningham, S. A., Kremen, C. and Tscharntke, T. (2007). Importance of pollinators in changing landscapes for world crops. *Proc. R. Soc. B* 274, 303-313. 10.1098/rspb.2006.3721 PMC1702377 17164193

[BIO061781C31] Kuo, Y., Lu, Y.-H., Lin, Y.-H., Lin, Y.-C. and Wu, Y.-L. (2023). Elevated temperature affects energy metabolism and behavior of bumblebees. *Insect Biochem. Mol. Biol.* 155, 103932. 10.1016/j.ibmb.2023.103932 36921734

[BIO061781C32] Laußer, S. and Kurze, C. (2025). Heatwave length and severity drive development disruption in a key pollinator. *bioRxiv*. 10.1101/2025.03.26.645459

[BIO061781C33] Lenth, R. and Lenth, M. R. (2018). Package ‘lsmeans’. *Am. Stat.* 34, 216-221.

[BIO061781C34] Lhotka, O. and Kyselý, J. (2024). Three-dimensional analysis reveals diverse heat wave types in Europe. *Commun. Earth Environ.* 5, 323. 10.1038/s43247-024-01497-2

[BIO061781C35] Lhotka, O., Kyselý, J. and Farda, A. (2018). Climate change scenarios of heat waves in Central Europe and their uncertainties. *Theor. Appl. Clim.* 131, 1043-1054. 10.1007/s00704-016-2031-3

[BIO061781C36] Ma, C.-S., Ma, G. and Pincebourde, S. (2021). Survive a warming climate: insect responses to extreme high temperatures. *Annu. Rev. Entomol.* 66, 163-184. 10.1146/annurev-ento-041520-074454 32870704

[BIO061781C37] Maebe, K., Hart, A. F., Marshall, L., Vandamme, P., Vereecken, N. J., Michez, D. and Smagghe, G. (2021). Bumblebee resilience to climate change, through plastic and adaptive responses. *Glob. Change Biol.* 27, 4223-4237. 10.1111/gcb.15751 PMC13420811 34118096

[BIO061781C38] Martinet, B., Dellicour, S., Ghisbain, G., Przybyla, K., Zambra, E., Lecocq, T., Boustani, M., Baghirov, R., Michez, D. and Rasmont, P. (2021a). Global effects of extreme temperatures on wild bumblebees. *Conserv. Biol.* 35, 1507-1518. 10.1111/cobi.13685 33319368

[BIO061781C39] Martinet, B., Zambra, E., Przybyla, K., Lecocq, T., Anselmo, A., Nonclercq, D., Rasmont, P., Michez, D. and Hennebert, E. (2021b). Mating under climate change: Impact of simulated heatwaves on the reproduction of model pollinators. *Funct. Ecol.* 35, 739-752. 10.1111/1365-2435.13738

[BIO061781C40] McKechnie, A. E. and Wolf, B. O. (2010). Climate change increases the likelihood of catastrophic avian mortality events during extreme heat waves. *Biol. Lett.* 6, 253-256. 10.1098/rsbl.2009.0702 19793742 PMC2865035

[BIO061781C41] Nijhout, H. F. and Williams, C. M. (1974). Control of moulting and metamorphosis in the tobacco hornworm, *Manduca sexta* (L.): growth of the last-instar larva and the decision to pupate. *J. Exp. Biol.* 61, 481-491. 10.1242/jeb.61.2.481 4443740

[BIO061781C42] Nooten, S. S., Korten, H., Schmitt, T. and Kárpáti, Z. (2024). The heat is on: reduced detection of floral scents after heatwaves in bumblebees. *Proc. R. Soc. B* 291, 20240352. 10.1098/rspb.2024.0352 PMC11349442 39191280

[BIO061781C43] Pereboom, J., Velthuis, H. and Duchateau, M. (2003). The organisation of larval feeding in bumblebees (Hymenoptera, Apidae) and its significance to caste differentiation. *Insectes Soc.* 50, 127-133. 10.1007/s00040-003-0639-7

[BIO061781C44] Perkins-Kirkpatrick, S. E. and Lewis, S. C. (2020). Increasing trends in regional heatwaves. *Nat. Commun.* 11, 3357. 10.1038/s41467-020-16970-7 32620857 PMC7334217

[BIO061781C45] Perl, C. D., Johansen, Z. B., Moradinour, Z., Guiraud, M., Restrepo, C. E., Wen Jie, V., Miettinen, A. and Baird, E. (2022). Heatwave-like events during development are sufficient to impair bumblebee worker responses to sensory stimuli. *Front. Ecol. Evol.* 9, 1-10. 10.3389/fevo.2021.776830

[BIO061781C46] Potts, S. G., Imperatriz-Fonseca, V., Ngo, H. T., Aizen, M. A., Biesmeijer, J. C., Breeze, T. D., Dicks, L. V., Garibaldi, L. A., Hill, R., Settele, J. et al. (2016). Safeguarding pollinators and their values to human well-being. *Nature* 540, 220-229. 10.1038/nature20588 27894123

[BIO061781C47] Quinlan, G. M., Feuerborn, C., Hines, H. M. and Grozinger, C. M. (2023). Beat the heat: thermal respites and access to food associated with increased bumble bee heat tolerance. *J. Exp. Biol.* 226, jeb245924. 10.1242/jeb.245924 37578032 PMC10508702

[BIO061781C48] R Core Team. (2024). *_R: A Language and Environment for Statistical Computing_*. R Foundation for Statistical Computing, Vienna, Austria. https://www.R-project.org/

[BIO061781C49] Rabasa, C. and Dickson, S. L. (2016). Impact of stress on metabolism and energy balance. *Curr. Opin. Behav. Sci.* 9, 71-77. 10.1016/j.cobeha.2016.01.011

[BIO061781C50] Rasmont, P. and Iserbyt, S. (2012). The Bumblebees Scarcity Syndrome: are heat waves leading to local extinctions of bumblebees (Hymenoptera: Apidae: *Bombus*)? *Annal. Soc. Entomol. France (N.S.)* 48, 275-280. 10.1080/00379271.2012.10697776

[BIO061781C51] Ratnayake, H. U., Kearney, M. R., Govekar, P., Karoly, D. and Welbergen, J. A. (2019). Forecasting wildlife die-offs from extreme heat events. *Anim. Conserv.* 22, 386-395. 10.1111/acv.12476

[BIO061781C52] Rita, A., Camarero, J. J., Nolè, A., Borghetti, M., Brunetti, M., Pergola, N., Serio, C., Vicente-Serrano, S. M., Tramutoli, V. and Ripullone, F. (2020). The impact of drought spells on forests depends on site conditions: the case of 2017 summer heat wave in southern Europe. *Glob. Change Biol.* 26, 851-863. 10.1111/gcb.14825 31486191

[BIO061781C53] Schmehl, D. R., Tomé, H. V., Mortensen, A. N., Martins, G. F. and Ellis, J. D. (2016). Protocol for the in vitro rearing of honey bee (Apis mellifera L.) workers. *J. Apic. Res.* 55, 113-129. 10.1080/00218839.2016.1203530

[BIO061781C54] Sepúlveda, Y., Nicholls, E., Schuett, W. and Goulson, D. (2024). Heatwave-like events affect drone production and brood-care behaviour in bumblebees. *PeerJ* 12, e17135. 10.7717/peerj.17135 38529302 PMC10962346

[BIO061781C55] Soroye, P., Newbold, T. and Kerr, J. (2020). Climate change contributes to widespread declines among bumble bees across continents. *Science* 367, 685-688. 10.1126/science.aax8591 32029628

[BIO061781C56] Stillman, J. H. (2019). Heat waves, the new normal: summertime temperature extremes will impact animals, ecosystems, and human communities. *Physiology* 34, 86-100. 10.1152/physiol.00040.2018 30724132

[BIO061781C57] Suzuki-Ohno, Y., Yokoyama, J., Nakashizuka, T. and Kawata, M. (2020). Estimating possible bumblebee range shifts in response to climate and land cover changes. *Sci. Rep.* 10, 19622. 10.1038/s41598-020-76164-5 33184331 PMC7661518

[BIO061781C58] Theodorou, P., Kühn, O., Baltz, L. M., Wild, C., Rasti, S. L., Bucksch, C. R., Strohm, E., Paxton, R. J. and Kurze, C. (2022). Bumble bee colony health and performance vary widely across the urban ecosystem. *J. Anim. Ecol.* 91, 2135-2148. 10.1111/1365-2656.13797 36002939

[BIO061781C59] Tian, L. and Hines, H. M. (2018). Morphological characterization and staging of bumble bee pupae. *PeerJ* 6, e6089. 10.7717/peerj.6089 30588402 PMC6302898

[BIO061781C60] Vanderplanck, M., Michez, D., Albrecht, M., Attridge, M., Babin, A., Bottero, I., Breeze, T., Brown, M., Chauzat, M.-P., Cini, E. et al. (2021). Monitoring bee health in European agroecosystems using wing morphology and fat bodies. *One Ecosystem* 6, e63653. 10.3897/oneeco.6.e63653

[BIO061781C61] Vogt, F. D. (1986). Thermoregulation in bumblebee colonies. I. Thermoregulatory versus brood-maintenance behaviors during acute changes in ambient temperature. *Physiol. Zool.* 59, 55-59. 10.1086/physzool.59.1.30156090

[BIO061781C62] Weidenmüller, A., Kleineidam, C. and Tautz, J. (2002). Collective control of nest climate parameters in bumblebee colonies. *Anim. Behav.* 63, 1065-1071. 10.1006/anbe.2002.3020

[BIO061781C63] Zambra, E., Martinet, B., Brasero, N., Michez, D. and Rasmont, P. (2020). Hyperthermic stress resistance of bumblebee males: test case of Belgian species. *Apidologie* 51, 911-920. 10.1007/s13592-020-00771-4

[BIO061781C64] Zhao, Z.-J., Hambly, C., Shi, L.-L., Bi, Z.-Q., Cao, J. and Speakman, J. R. (2020). Late lactation in small mammals is a critically sensitive window of vulnerability to elevated ambient temperature. *Proc. Natl. Acad. Sci. USA* 117, 24352-24358. 10.1073/pnas.2008974117 32929014 PMC7533837

[BIO061781C65] Zuur, A. F., Ieno, E. N., Walker, N., Saveliev, A. A. and Smith, G. M. (2009). Mixed effects models and extensions in ecology with R (Vol. 574, p. 574). New York: springer. 10.1007/978-0-387-87458-6

