# Supplementary material for: Experimental short-term heatwaves negatively impact body weight gain and survival during larval development in Bombus terrestris L. (Hymenoptera: Apidae)
Source: Biol Open. 2025 Apr 25;14(4):bio061781. doi: 10.1242/bio.061781 (PMC12045637; doi:10.1242/bio.061781)
Supplement: Supplementary information [file biolopen-14-061781-s1.pdf]

Table S1.

Available for download at
https://journals.biologists.com/bio/article-lookup/doi/10.1242/bio.061781#supplementary-data

Table S2.

Available for download at
https://journals.biologists.com/bio/article-lookup/doi/10.1242/bio.061781#supplementary-data

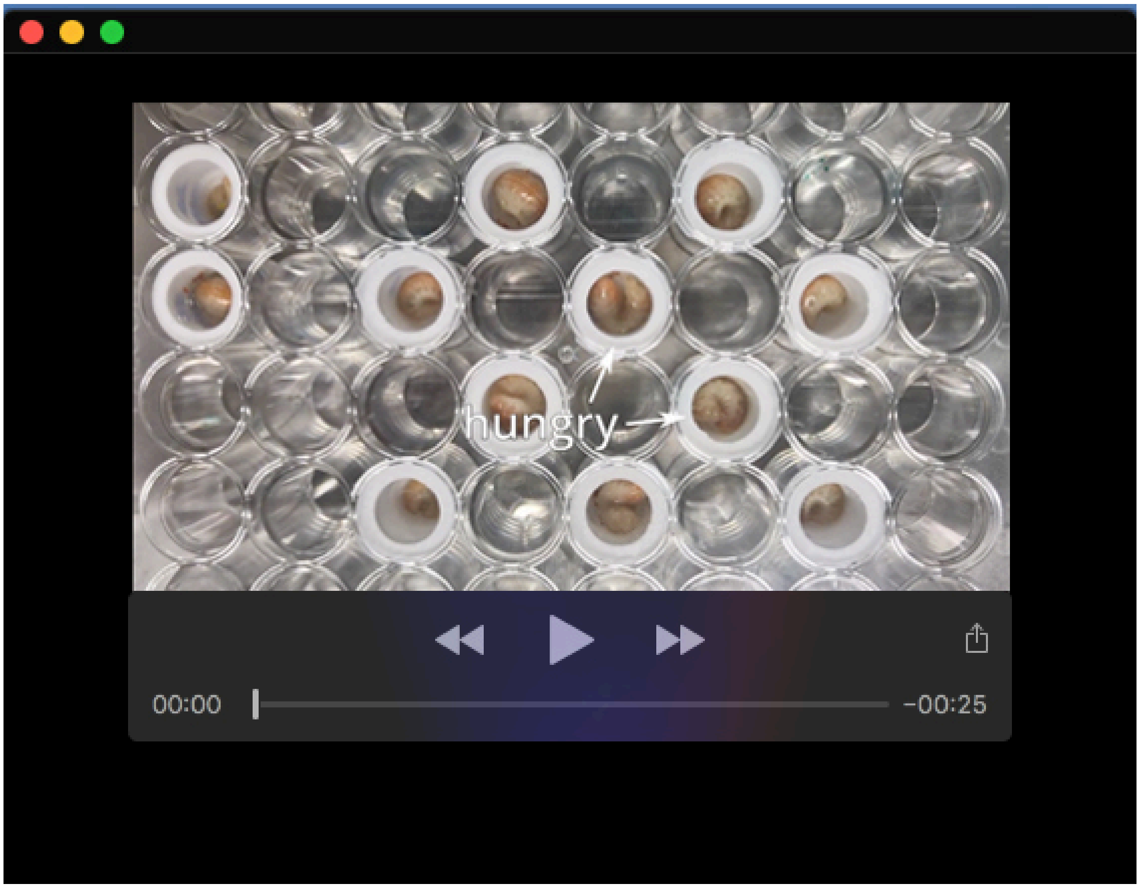

Movie 1.

Biology Open • Supplementary information

Biology Open (2025): doi:10.1242/bio.061781: Supplementary information

Statistical analysis: Influence of simulated heat waves on larval development in *Bombus terrestris*

Christoph Kurze

2024-12-08

This R Markdown file presents the statistical analysis conducted on the data outlined in our manuscript titled "Influence of simulated heat waves on larval development in *Bombus terrestris*". The experiment involved the in-vitro rearing of larvae subjected to a 4-day heat wave simulation, with temperatures set at 37°C and 38°C, contrasted against a control group maintained at 34°C.

Libraries

```r
rm(list = ls())
library(Rmisc)
```

```
## Loading required package: lattice

## Loading required package: plyr
```

```r
library(readr)
library(tidyverse)
```

```
## -- Attaching core tidyverse packages ------------------------ tidyverse 2.0.0 --
## v dplyr     1.1.4     v purrr     1.0.2
## v forcats   1.0.0     v stringr   1.5.1
## v ggplot2   3.5.1     v tibble    3.2.1
## v lubridate 1.9.3     v tidyr     1.3.1

## -- Conflicts ------------------------------------------ tidyverse_conflicts() --
## x dplyr::arrange()   masks plyr::arrange()
## x purrr::compact()   masks plyr::compact()
## x dplyr::count()     masks plyr::count()
## x dplyr::desc()      masks plyr::desc()
## x dplyr::failwith()  masks plyr::failwith()
## x dplyr::filter()    masks stats::filter()
## x dplyr::id()        masks plyr::id()
## x dplyr::lag()       masks stats::lag()
## x dplyr::mutate()    masks plyr::mutate()
## x dplyr::rename()    masks plyr::rename()
## x dplyr::summarise() masks plyr::summarise()
## x dplyr::summarize() masks plyr::summarize()
## i Use the conflicted package (<http://conflicted.r-lib.org/>) to force all conflicts to become errors
```


Biology Open • Supplementary information

```r
library(ggsignif)
library(ggforce)
library(gridExtra)
```

```
##
## Attaching package: 'gridExtra'
##
## The following object is masked from 'package:dplyr':
##
##     combine
```

```r
library(emmeans)
```

```
## Welcome to emmeans.
## Caution: You lose important information if you filter this package's results.
## See '? untidy'
```

```r
library(glmmTMB)
library(DHARMa)
```

```
## This is DHARMa 0.4.6. For overview type '?DHARMa'. For recent changes, type news(package = 'DHARMa')
```

```r
library(lme4)
```

```
## Loading required package: Matrix
##
## Attaching package: 'Matrix'
##
## The following objects are masked from 'package:tidyr':
##
##     expand, pack, unpack
```

```r
library(car)
```

```
## Loading required package: carData
##
## Attaching package: 'car'
##
## The following object is masked from 'package:dplyr':
##
##     recode
##
## The following object is masked from 'package:purrr':
##
##     some
```

```r
library(lmtest)
```


Biology Open • Supplementary information

```
## Loading required package: zoo
##
## Attaching package: 'zoo'
##
## The following objects are masked from 'package:base':
##
##     as.Date, as.Date.numeric
```

```r
# Load data
Df <- read_csv("Data_heatwaves_larval_development.csv")
```

Load data, calculations and subsetting

```
## Rows: 289 Columns: 19
## -- Column specification --------------------------------------------------------
## Delimiter: ","
## chr   (5): id, colony, treatment, developmental_stage_reached, sex
## dbl  (10): mass_larvae_before, mass_larvae_after, mass_pupae, mass_adults, p...
## date  (4): collected, date_pupation, date_hatching, date_death
##
## i Use `spec()` to retrieve the full column specification for this data.
## i Specify the column types or set `show_col_types = FALSE` to quiet this message.
```

```r
Df$id <- as.factor(Df$id)
Df$colony <- as.factor(Df$colony)
Df$treatment <- as.factor(Df$treatment)
Df$mass_larvae_before <- as.numeric(Df$mass_larvae_before)
Df$mass_larvae_after <- as.numeric(Df$mass_larvae_after)
Df$mass_pupae <- as.numeric(Df$mass_pupae)
Df$mass_adults <- as.numeric(Df$mass_adults)
Df$collected <- as.Date(Df$collected)
Df$date_pupation <- as.Date(Df$date_pupation)
Df$date_hatching <- as.Date(Df$date_hatching)
Df$date_death <- as.Date(Df$date_death)
Df$developmental_stage_reached <- as.factor(Df$developmental_stage_reached)
Df$reached_adulthood <- as.numeric(Df$reached_adulthood)
Df$pupated <- as.numeric(Df$pupated)
Df$sex <- as.factor(Df$sex)
Df$itd <- as.numeric(Df$itd)
Df$headwidth <- as.numeric(Df$headwidth)
Df$dry_mass <- as.numeric(Df$dry_mass)
Df$fat <- as.numeric(Df$fat)

# Define the order of treatments
Df$treatment <- fct_recode(Df$treatment,
                        Ctrl = "Control")
treatment_order <- c("Ctrl", "37°C", "38°C")
Df <- Df %>%
  mutate(treatment = factor(treatment, levels = treatment_order))
# calculations
Df <- Df %>%
```


Biology Open • Supplementary information

```
  mutate(BWG_treatment = (mass_larvae_after - mass_larvae_before)) %>% # BWG = body weight gain
  mutate(BWG_to_pupation = (mass_pupae - mass_larvae_before)) %>%
  mutate(BWG_to_hatching = (mass_adults - mass_larvae_before)) %>%
  mutate(rel_BWG_treatment = (mass_larvae_after - mass_larvae_before)*100/mass_larvae_before) %>% # BWG = body weight g
  mutate(rel_BWG_to_pupation = (mass_pupae - mass_larvae_before)*100/mass_larvae_before) %>%
  mutate(rel_BWG_during_pupation = (mass_adults - mass_pupae)*100/mass_pupae) %>%
  mutate(days_to_pupation = as.numeric(difftime(date_pupation, collected, units = "days"))) %>%
  mutate(days_to_hatching = as.numeric(difftime(date_hatching, collected, units = "days")))

# Sub-setting
Df_34d <- Df %>%
  filter(treatment == "Ctrl")
Df_37C <- Df %>%
  filter(treatment == "37°C")
Df_38C <- Df %>%
  filter(treatment == "38°C")
```

Data summaries

```
# Sample size per colony and treatment
NCol <- Df %>%
  group_by(colony)%>%
  count(treatment)
NCol
```

```
## # A tibble: 15 x 3
## # Groups:   colony [5]
##    colony    treatment     n
##    <fct>     <fct>     <int>
##  1 Christina Ctrl         17
##  2 Christina 37°C         17
##  3 Christina 38°C         17
##  4 Jane      Ctrl         20
##  5 Jane      37°C         17
##  6 Jane      38°C         17
##  7 Karl      Ctrl         17
##  8 Karl      37°C         16
##  9 Karl      38°C         16
## 10 Konrad    Ctrl         20
## 11 Konrad    37°C         26
## 12 Konrad    38°C         21
## 13 Rosalind  Ctrl         27
## 14 Rosalind  37°C         20
## 15 Rosalind  38°C         21
```

```
# Sample size per sex
NSex <- Df %>%
  filter(reached_adulthood == 1) %>%
  group_by(sex)%>%
  count(treatment)
NSex
```


Biology Open • Supplementary information

```
## # A tibble: 6 x 3
## # Groups:   sex [2]
##   sex    treatment     n
##   <fct>  <fct>     <int>
## 1 female Ctrl         23
## 2 female 37°C         29
## 3 female 38°C         12
## 4 male   Ctrl         11
## 5 male   37°C          4
## 6 male   38°C          4
```

```
# Larvae body weight at the start of the experiment
MassBeforeTreat <- summarySE(Df, measurevar="mass_larvae_before", groupvars=c("treatment"))
MassBeforeTreat
```

```
##   treatment   N mass_larvae_before         sd          se         ci
## 1      Ctrl 101          0.1568505 0.06082166 0.006051982 0.01200696
## 2      37°C  96          0.1626500 0.06046253 0.006170931 0.01225085
## 3      38°C  92          0.1617043 0.07600878 0.007924463 0.01574097
```

Calculation of the mortality rates

```
# Calculation of the mortality rates per developmental stage for each treatment ----
Df_mortality <- Df %>%
  count(treatment, developmental_stage_reached) %>%
  group_by(treatment) %>%
  mutate(total = sum(n)) %>%
  ungroup() %>%
  mutate(mortality_rate = n / total) %>%
  select(treatment, developmental_stage_reached, total, mortality_rate, n)
Df_mortality
```

```
## # A tibble: 9 x 5
##   treatment developmental_stage_reached total mortality_rate     n
##   <fct>     <fct>                       <int>          <dbl> <int>
## 1 Ctrl      Adult                         101          0.337    34
## 2 Ctrl      Larva                         101          0.337    34
## 3 Ctrl      Pupa                          101          0.327    33
## 4 37°C      Adult                          96          0.344    33
## 5 37°C      Larva                          96          0.354    34
## 6 37°C      Pupa                           96          0.302    29
## 7 38°C      Adult                          92          0.174    16
## 8 38°C      Larva                          92          0.457    42
## 9 38°C      Pupa                           92          0.370    34
```

```
# Calculation of the survival rate ----
Df_survival <- Df_mortality %>%
  filter(!is.na(treatment)) %>%
  group_by(treatment) %>%
  mutate(
    survival_rate = case_when(
```


Biology Open • Supplementary information

```r
      developmental_stage_reached == "Adult" ~ 1 - sum(mortality_rate[developmental_stage_reached %in% c("Larva", "Pupa[illegible]
      developmental_stage_reached == "Pupa" ~ 1 - mortality_rate[developmental_stage_reached == "Larva"],
      TRUE ~ NA_real_  # for larvae, set survival rate as NA
    ),
    survival = survival_rate*100
  ) %>%
  ungroup()


Df_survival <- Df_mortality %>%
  group_by(treatment) %>%
  mutate(
    survival_rate = case_when(
      developmental_stage_reached == "Adult" ~ 1 - sum(mortality_rate[developmental_stage_reached %in% c("Larva", "Pupa[illegible]
      developmental_stage_reached == "Pupa" ~ 1 - mortality_rate[developmental_stage_reached == "Larva"],
      TRUE ~ NA_real_  # for larvae, set survival rate as NA
    ),
    survival = survival_rate*100
    ) %>%
  ungroup()
Df_survival
```

```
## # A tibble: 9 x 7
##   treatment developmental_stage_reached total mortality_rate     n survival_rate
##   <fct>     <fct>                       <int>          <dbl> <int>         <dbl>
## 1 Ctrl      Adult                         101          0.337    34         0.337
## 2 Ctrl      Larva                         101          0.337    34        NA
## 3 Ctrl      Pupa                          101          0.327    33         0.663
## 4 37°C      Adult                          96          0.344    33         0.344
## 5 37°C      Larva                          96          0.354    34        NA
## 6 37°C      Pupa                           96          0.302    29         0.646
## 7 38°C      Adult                          92          0.174    16         0.174
## 8 38°C      Larva                          92          0.457    42        NA
## 9 38°C      Pupa                           92          0.370    34         0.543
## # i 1 more variable: survival <dbl>
```

Analysis of survival

```r
# subset per developmental stage
survival_Pupae<- Df_survival %>%
  filter(developmental_stage_reached == "Pupa")
survival_Adults <- Df_survival %>%
  filter(developmental_stage_reached == "Adult")

# chi-squared test for survival to pupae stage
cont_table <- xtabs(survival ~ treatment, data = survival_Pupae) # Create a contingency table
chi_squared_result <- chisq.test(cont_table)
chi_squared_result
```

```
##
##  Chi-squared test for given probabilities
```


Biology Open • Supplementary information

```
##
## data:  cont_table
## X-squared = 1.3579, df = 2, p-value = 0.5072
```

```
# chi-squared tests for survival to adult stage
cont_table <- xtabs(survival ~ treatment, data = survival_Adults)
chi_squared_result <- chisq.test(cont_table)
chi_squared_result
```

```
##
##  Chi-squared test for given probabilities
##
## data:  cont_table
## X-squared = 6.4817, df = 2, p-value = 0.03913
```

```
# Perform pairwise comparisons with Benjamini-Hochberg correction
counts <- survival_Adults$n
total <- survival_Adults$total

pairwise_result <- pairwise.prop.test(counts, total, p.adjust.method = "BH")
treatment_names <- survival_Adults$treatment # Define the treatment names

comparison_matrix <- pairwise_result$p.value
dimnames(comparison_matrix) <- list(treatment_names[-1], treatment_names[-length(treatment_names)])
comparison_matrix
```

```
##            Ctrl       37°C
## 37°C 1.00000000         NA
## 38°C 0.02376039 0.02376039
```

Figure 1ac

```
Fig1a <- ggplot(data = survival_Pupae, aes(x = treatment, y = survival, fill = treatment)) +
  geom_bar(stat = "identity") +
  labs(title = "(a)",
       x = "",
       y = "Pupated (%)") +
  scale_fill_manual(values = c(alpha("#0072B2", 1),
                               alpha("#E69F00", 1),
                               alpha("#D55E00", 1))) +
  scale_y_continuous(expand = expansion(mult = c(0.0, 0.1))) +
  geom_text(data = survival_Pupae, aes(x = treatment, y = 2, label = paste0("n=", total)),
            size = 3, vjust = 0 , hjust = 0.5, color = "white") +
  theme_classic() +
  theme(
    plot.title = element_text(hjust = 0, vjust = 1),
    text = element_text(size = 10),
    legend.position = "none",
    plot.margin = margin(0, 1, 0, 0, "cm")
    )
```


Biology Open • Supplementary information

```r
Fig1c <- ggplot(data = survival_Adults, aes(x = treatment, y = survival, fill = treatment)) +
  geom_bar(stat = "identity") +
  labs(title = "(c)",
       x = "Treatment",
       y = "Emerged (%)") +
  scale_fill_manual(values = c(alpha("#0072B2", 1),
                               alpha("#E69F00", 1),
                               alpha("#D55E00", 1))) +
  scale_y_continuous(expand = expansion(mult = c(0.0, 0.1))) +
  geom_text(data = data.frame(x = c("38°C"),
                              y = c(18),
                              label = c("*")), # from post-hoc test
            aes(x = x, y = y, label = label), size = 5, color = "black", vjust = -0.5, inherit.aes = FALSE) +
  geom_text(data = survival_Adults, aes(x = treatment, y = 1, label = paste0("n=", total)),
            size = 3, vjust = 0 , hjust = 0.5, color = "white") +
  theme_classic() +
  theme(
    plot.title = element_text(hjust = 0, vjust = 1),
    text = element_text(size = 10),
    legend.position = "none",
    plot.margin = margin(0, 1, 0, 0, "cm")
    )
```

```r
Df2 <- Df[!is.na(Df$rel_BWG_to_pupation), ]
Df4 <- Df2 %>% filter(!is.na(days_to_pupation))
# NULL MODEL
m0e <- glmmTMB(
  days_to_pupation ~ 1 + (1|colony),
  family = Gamma(link = "log"), Df4)
# FULL MODEL
m4f <- glmmTMB(
  days_to_pupation ~ treatment + (1|colony),
  family = Gamma(link = "log"), Df4)
summary(m4f)
```

Analysis of developmental times to pupation

```
##  Family: Gamma  ( log )
## Formula:          days_to_pupation ~ treatment + (1 | colony)
## Data: Df4
##
##      AIC      BIC   logLik deviance df.resid
##    851.0    867.1   -420.5    841.0      179
##
## Random effects:
##
## Conditional model:
##  Groups Name        Variance Std.Dev.
##  colony (Intercept) 0.04047  0.2012
## Number of obs: 184, groups:  colony, 5
```

```
##
## Dispersion estimate for Gamma family (sigma^2): 0.246
##
## Conditional model:
##                Estimate Std. Error z value Pr(>|z|)
## (Intercept)     1.72193    0.10924  15.763   <2e-16 ***
## treatment37°C -0.14271    0.09037  -1.579    0.114
## treatment38°C -0.07437    0.09381  -0.793    0.428
## ---
## Signif. codes:  0 '***' 0.001 '**' 0.01 '*' 0.05 '.' 0.1 ' ' 1
```

```
Anova(m4f)
```

```
## Analysis of Deviance Table (Type II Wald chisquare tests)
##
## Response: days_to_pupation
##            Chisq Df Pr(>Chisq)
## treatment 2.4971  2     0.2869
```

```
### Comparing slopes
em_means4f <- emmeans(m4f, ~ treatment) # Obtain marginal means
em_means4f
```

```
##  treatment emmean    SE  df asymp.LCL asymp.UCL
##  Ctrl        1.72 0.109 Inf      1.51      1.94
##  37°C        1.58 0.110 Inf      1.36      1.80
##  38°C        1.65 0.116 Inf      1.42      1.87
##
## Results are given on the log (not the response) scale.
## Confidence level used: 0.95
```

```
slope_comparisons4f <- contrast(em_means4f, interaction = "pairwise")
slope_comparisons4f
```

```
##  treatment_pairwise estimate     SE  df z.ratio p.value
##  Ctrl - 37°C          0.1427 0.0904 Inf   1.579  0.1143
##  Ctrl - 38°C          0.0744 0.0938 Inf   0.793  0.4279
##  37°C - 38°C         -0.0683 0.0949 Inf  -0.720  0.4713
##
## Results are given on the log (not the response) scale.
```

```
# Lower AIC or BIC values indicate better-fitting models
AIC(m0e, m4f)
```

```
##     df      AIC
## m0e  3 849.4666
## m4f  5 850.9828
```

```
BIC(m0e, m4f)
```

```
##     df      BIC
## m0e  3 859.1114
## m4f  5 867.0575
```

```
# Compared to NULL model
lrtest(m0e, m4f)
```

```
## Likelihood ratio test
##
## Model 1: days_to_pupation ~ 1 + (1 | colony)
## Model 2: days_to_pupation ~ treatment + (1 | colony)
##   #Df  LogLik Df  Chisq Pr(>Chisq)
## 1   3 -421.73
## 2   5 -420.49  2 2.4838     0.2888
```

```
anova(m0e, m4f)
```

```
## Data: Df4
## Models:
## m0e: days_to_pupation ~ 1 + (1 | colony), zi=~0, disp=~1
## m4f: days_to_pupation ~ treatment + (1 | colony), zi=~0, disp=~1
##     Df    AIC    BIC  logLik deviance  Chisq Chi Df Pr(>Chisq)
## m0e  3 849.47 859.11 -421.73   843.47
## m4f  5 850.98 867.06 -420.49   840.98 2.4838      2     0.2888
```

```
# Model diagnostics
testDispersion(m4f)
```

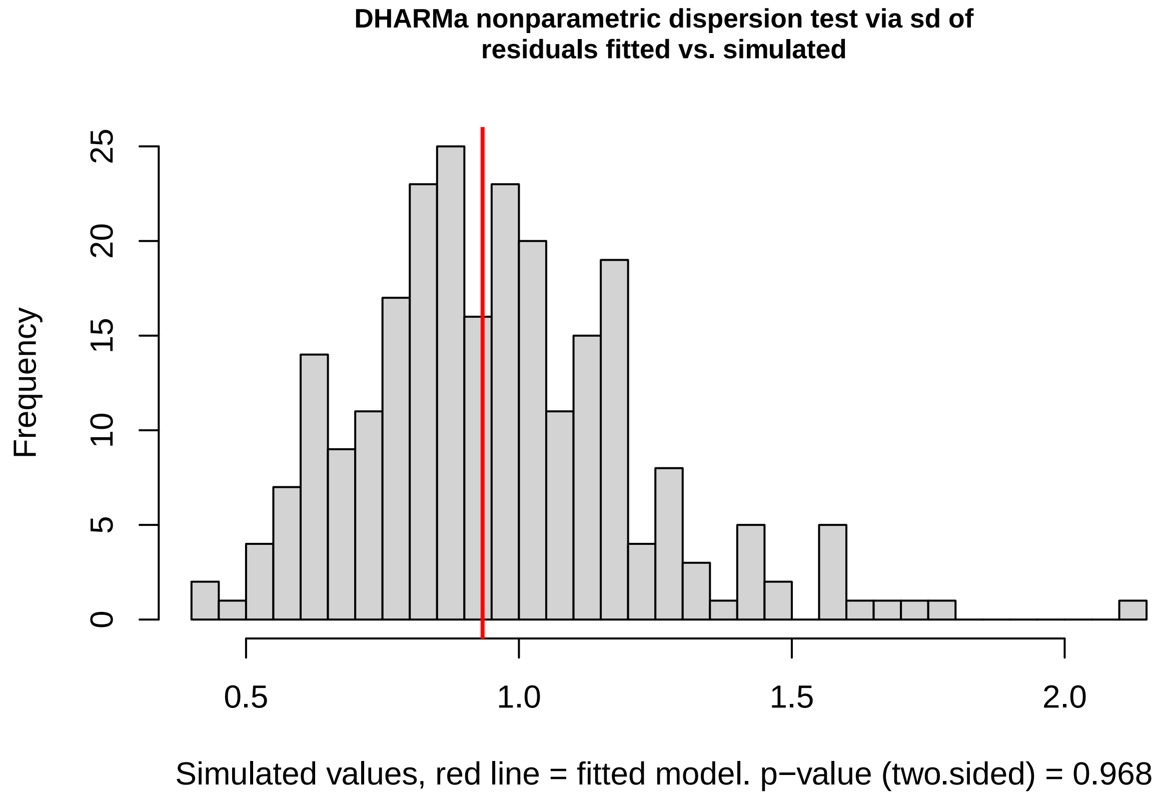

Biology Open • Supplementary information

```
##
##  DHARMa nonparametric dispersion test via sd of residuals fitted vs.
##  simulated
##
## data:  simulationOutput
## dispersion = 0.97025, p-value = 0.968
## alternative hypothesis: two.sided
```

```r
simulationOutput <- simulateResiduals(fittedModel = m4f, plot = F)
residuals(simulationOutput)
```

```
##   [1] 0.704 0.372 0.632 0.952 0.824 0.852 0.628 0.924 0.944 0.712 0.596 0.208
##  [13] 0.400 0.432 0.064 0.440 0.932 0.856 0.820 0.740 0.156 0.916 0.924 0.492
##  [25] 0.892 0.784 0.580 0.380 0.704 0.076 0.904 0.556 0.388 0.344 0.792 0.784
##  [37] 0.776 0.780 0.968 0.360 0.588 0.808 0.800 0.088 0.288 0.588 0.504 0.588
##  [49] 0.688 0.612 0.360 0.792 0.556 0.476 0.392 0.396 0.812 0.340 0.668 0.408
##  [61] 0.204 0.600 0.116 0.756 0.912 0.972 0.996 0.868 0.768 0.712 0.924 0.904
##  [73] 0.532 0.100 0.500 0.620 0.388 0.708 0.284 0.656 0.604 0.416 0.360 0.896
##  [85] 0.556 0.892 0.248 0.496 0.764 0.184 0.824 0.124 0.192 0.820 0.804 0.436
##  [97] 0.320 0.052 0.992 0.264 0.088 0.444 0.452 0.340 0.608 0.100 0.388 0.012
## [109] 0.328 0.388 0.004 0.008 0.472 0.640 0.480 0.380 0.592 0.216 0.564 0.596
## [121] 0.796 0.636 0.396 0.504 0.056 0.016 0.228 0.004 0.004 0.108 0.688 0.448
## [133] 0.408 0.364 0.540 0.224 0.392 0.480 0.492 0.204 0.580 0.616 0.232 0.560
## [145] 0.452 0.456 0.576 0.368 0.084 0.008 0.072 0.364 0.076 0.772 0.044 0.400
## [157] 0.104 0.992 0.992 0.248 0.628 0.700 0.216 0.620 0.108 0.416 0.264 0.144
## [169] 0.060 0.096 0.232 0.080 0.376 0.312 0.804 0.508 0.492 0.640 0.632 0.652
## [181] 0.004 0.156 0.928 0.960
```

```r
residuals(simulationOutput, quantileFunction = qnorm, outlierValues = c(-7,7))
```

```
##   [1]  0.53594003 -0.32656093  0.33715508  1.66456286  0.93071695  1.04504970
##   [7]  0.32656093  1.43250272  1.58926756  0.55923698  0.24300697 -0.81338039
##  [13] -0.25334710 -0.17128459 -1.52203624 -0.15096922  1.49085336  1.06251930
##  [19]  0.91536509  0.64334541 -1.01103433  1.37865873  1.43250272 -0.02005437
##  [25]  1.23723460  0.78577383  0.20189348 -0.30548079  0.53594003 -1.43250272
##  [31]  1.30468539  0.14083537 -0.28453554 -0.40157070  0.81338039  0.78577383
##  [37]  0.75875354  0.77219321  1.85217986 -0.35845879  0.22240323  0.87054983
##  [43]  0.84162123 -1.35317415 -0.55923698  0.22240323  0.01002668  0.22240323
##  [49]  0.49018923  0.28453554 -0.35845879  0.81338039  0.14083537 -0.06019541
##  [55] -0.27411012 -0.26371440  0.88529045 -0.41246313  0.43439724 -0.23269275
##  [61] -0.82741832  0.25334710 -1.19522278  0.69349335  1.35317415  1.91103565
##  [67]  2.65206981  1.11698673  0.73227620  0.55923698  1.43250272  1.30468539
##  [73]  0.08029831 -1.28155157  0.00000000  0.30548079 -0.28453554  0.54755135
##  [79] -0.57099947  0.40157070  0.26371440 -0.21213720 -0.35845879  1.25908398
##  [85]  0.14083537  1.23723460 -0.68079692 -0.01002668  0.71922873 -0.90022599
##  [91]  0.93071695 -1.15522085 -0.87054983  0.91536509  0.85599599 -0.16111859
##  [97] -0.46769880 -1.62576339  2.40891555 -0.63106198 -1.35317415 -0.14083537
## [103] -0.12060993 -0.41246313  0.27411012 -1.28155157 -0.28453554 -2.25712924
## [109] -0.44544251 -0.28453554 -2.65206981 -2.40891555 -0.07024331  0.35845879
## [115] -0.05015358 -0.30548079  0.23269275 -0.78577383  0.16111859  0.24300697
## [121]  0.82741832  0.34778720 -0.26371440  0.01002668 -1.58926756 -2.14441062
## [127] -0.74544955 -2.65206981 -2.65206981 -1.23723460  0.49018923 -0.13071597
## [133] -0.23269275 -0.34778720  0.10043372 -0.75875354 -0.27411012 -0.05015358
```


Biology Open • Supplementary information

```
## [139] -0.02005437 -0.82741832  0.20189348  0.29499199 -0.73227620  0.15096922
## [145] -0.12060993 -0.11051620  0.19167090 -0.33715508 -1.37865873 -2.40891555
## [151] -1.46105627 -0.34778720 -1.43250272  0.74544955 -1.70604340 -0.25334710
## [157] -1.25908398  2.40891555  2.40891555 -0.68079692  0.32656093  0.52440051
## [163] -0.78577383  0.30548079 -1.23723460 -0.21213720 -0.63106198 -1.06251930
## [169] -1.55477359 -1.30468539 -0.73227620 -1.40507156 -0.31600330 -0.49018923
## [175]  0.85599599  0.02005437 -0.02005437  0.35845879  0.33715508  0.39072570
## [181] -2.65206981 -1.01103433  1.46105627  1.75068607
```

```r
plot(simulationOutput)
```

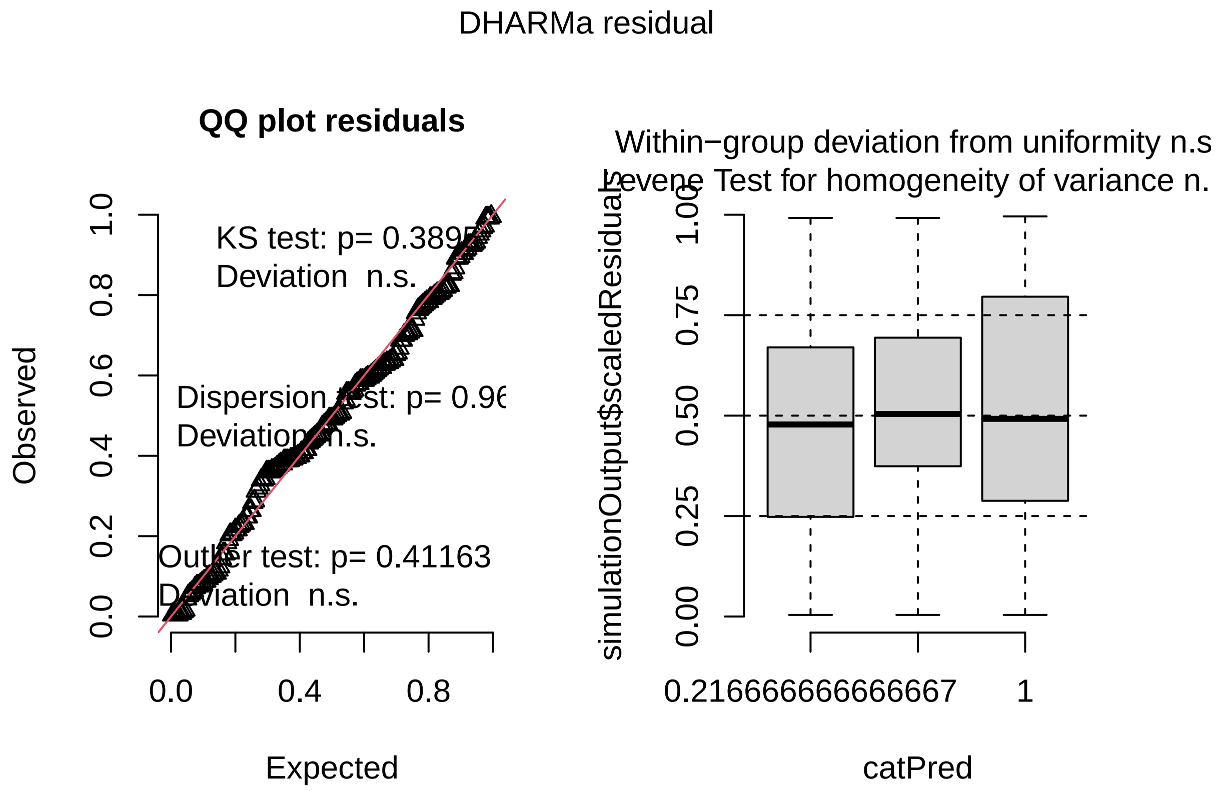


```r
# non-parametric alternative for treatment effect
kruskal.test(mass_larvae_before ~ treatment, data = Df)
```

```
##
##  Kruskal-Wallis rank sum test
##
## data:  mass_larvae_before by treatment
## Kruskal-Wallis chi-squared = 0.37302, df = 2, p-value = 0.8299
```

```r
kruskal.test(days_to_pupation ~ treatment, data = Df4)
```

```
##
##  Kruskal-Wallis rank sum test
```


Biology Open • Supplementary information

```
##
## data:  days_to_pupation by treatment
## Kruskal-Wallis chi-squared = 2.7244, df = 2, p-value = 0.2561
```

```
# non-parametric alternative for mass effect
cor.test(Df4$mass_larvae_before, Df4$days_to_pupation, method = "spearman")
```

Correlations

```
## Warning in cor.test.default(Df4$mass_larvae_before, Df4$days_to_pupation, :
## Cannot compute exact p-value with ties

##
##  Spearman's rank correlation rho
##
## data:  Df4$mass_larvae_before and Df4$days_to_pupation
## S = 1221411, p-value = 0.01658
## alternative hypothesis: true rho is not equal to 0
## sample estimates:
##        rho
## -0.1764476
```

```
cor.test(Df4$mass_larvae_before, Df4$days_to_pupation, method = "kendall")
```

```
##
##  Kendall's rank correlation tau
##
## data:  Df4$mass_larvae_before and Df4$days_to_pupation
## z = -2.6726, p-value = 0.007526
## alternative hypothesis: true tau is not equal to 0
## sample estimates:
##        tau
## -0.1401035
```

```
# non-parametric alternative for relative body weight gain effect
cor.test(Df4$rel_BWG_to_pupation, Df4$days_to_pupation, method = "spearman")
```

```
## Warning in cor.test.default(Df4$rel_BWG_to_pupation, Df4$days_to_pupation, :
## Cannot compute exact p-value with ties

##
##  Spearman's rank correlation rho
##
## data:  Df4$rel_BWG_to_pupation and Df4$days_to_pupation
## S = 291678, p-value < 2.2e-16
## alternative hypothesis: true rho is not equal to 0
## sample estimates:
##       rho
## 0.7190595
```


Biology Open • Supplementary information

```
cor.test(Df4$rel_BWG_to_pupation, Df4$days_to_pupation, method = "kendall")
```

```
##
##  Kendall's rank correlation tau
##
## data:  Df4$rel_BWG_to_pupation and Df4$days_to_pupation
## z = 10.454, p-value < 2.2e-16
## alternative hypothesis: true tau is not equal to 0
## sample estimates:
##       tau
## 0.5478779
```

```
Df5 <- Df[!is.na(Df$days_to_hatching), ]
DaysToHatching <- summarySE(Df5, measurevar="days_to_hatching", groupvars=c("treatment"))
DaysToHatching
```

Analysis of developmental times to emergence

```
##   treatment  N days_to_hatching       sd        se        ci
## 1      Ctrl 34         13.41176 3.144188 0.5392238 1.0970590
## 2      37°C 33         12.51515 1.839178 0.3201598 0.6521442
## 3      38°C 16         13.18750 2.762095 0.6905237 1.4718163
```

```
# NULL MODEL
m0f <- glmmTMB(
  days_to_hatching ~ 1 + (1|colony) ,
  family = Gamma(link = "log"), Df5)
# FULL MODEL
m5 <- glmmTMB(
  days_to_hatching ~ treatment + (1|colony),
  family = Gamma(link = "log"), Df5)
summary(m5)
```

```
##  Family: Gamma  ( log )
## Formula:          days_to_hatching ~ treatment + (1 | colony)
## Data: Df5
##
##      AIC      BIC   logLik deviance df.resid
##    369.3    381.4   -179.6    359.3       78
##
## Random effects:
##
## Conditional model:
##  Groups Name        Variance Std.Dev.
##  colony (Intercept) 0.01598  0.1264
## Number of obs: 83, groups:  colony, 5
##
## Dispersion estimate for Gamma family (sigma^2): 0.0234
##
```

```
## Conditional model:
##                Estimate Std. Error z value Pr(>|z|)
## (Intercept)    2.598590   0.063135   41.16   <2e-16 ***
## treatment37°C -0.028139   0.040524   -0.69    0.487
## treatment38°C -0.002489   0.047088   -0.05    0.958
## ---
## Signif. codes:  0 '***' 0.001 '**' 0.01 '*' 0.05 '.' 0.1 ' ' 1
```

```
Anova(m5)
```

```
## Analysis of Deviance Table (Type II Wald chisquare tests)
##
## Response: days_to_hatching
##            Chisq Df Pr(>Chisq)
## treatment 0.5426  2     0.7624
```

```
### Comparing slopes
em_means5 <- emmeans(m5, ~ treatment) # Obtain marginal means
em_means5
```

```
##  treatment emmean     SE  df asymp.LCL asymp.UCL
##  Ctrl        2.60 0.0631 Inf      2.47      2.72
##  37°C        2.57 0.0655 Inf      2.44      2.70
##  38°C        2.60 0.0705 Inf      2.46      2.73
##
## Results are given on the log (not the response) scale.
## Confidence level used: 0.95
```

```
slope_comparisons5 <- contrast(em_means5, interaction = "pairwise")
slope_comparisons5
```

```
##  treatment_pairwise estimate     SE  df z.ratio p.value
##  Ctrl - 37°C         0.02814 0.0405 Inf   0.694  0.4874
##  Ctrl - 38°C         0.00249 0.0471 Inf   0.053  0.9578
##  37°C - 38°C        -0.02565 0.0483 Inf  -0.531  0.5952
##
## Results are given on the log (not the response) scale.
```

```
# Lower AIC or BIC values indicate better-fitting models
AIC(m0f, m5)
```

```
##     df      AIC
## m0f  3 365.8103
## m5   5 369.2682
```

```
BIC(m0f, m5)
```

```
##     df      BIC
## m0f  3 373.0668
## m5   5 381.3624
```

```
# Compared to NULL model
lrtest(m0f, m5)
```

```
## Likelihood ratio test
##
## Model 1: days_to_hatching ~ 1 + (1 | colony)
## Model 2: days_to_hatching ~ treatment + (1 | colony)
##   #Df  LogLik Df Chisq Pr(>Chisq)
## 1   3 -179.91
## 2   5 -179.63  2 0.542     0.7626
```

```
anova(m0f, m5)
```

```
## Data: Df5
## Models:
## m0f: days_to_hatching ~ 1 + (1 | colony), zi=~0, disp=~1
## m5: days_to_hatching ~ treatment + (1 | colony), zi=~0, disp=~1
##     Df    AIC    BIC  logLik deviance Chisq Chi Df Pr(>Chisq)
## m0f  3 365.81 373.07 -179.91   359.81
## m5   5 369.27 381.36 -179.63   359.27 0.542      2     0.7626
```

```
# Model diagnostics
testDispersion(m5)
```

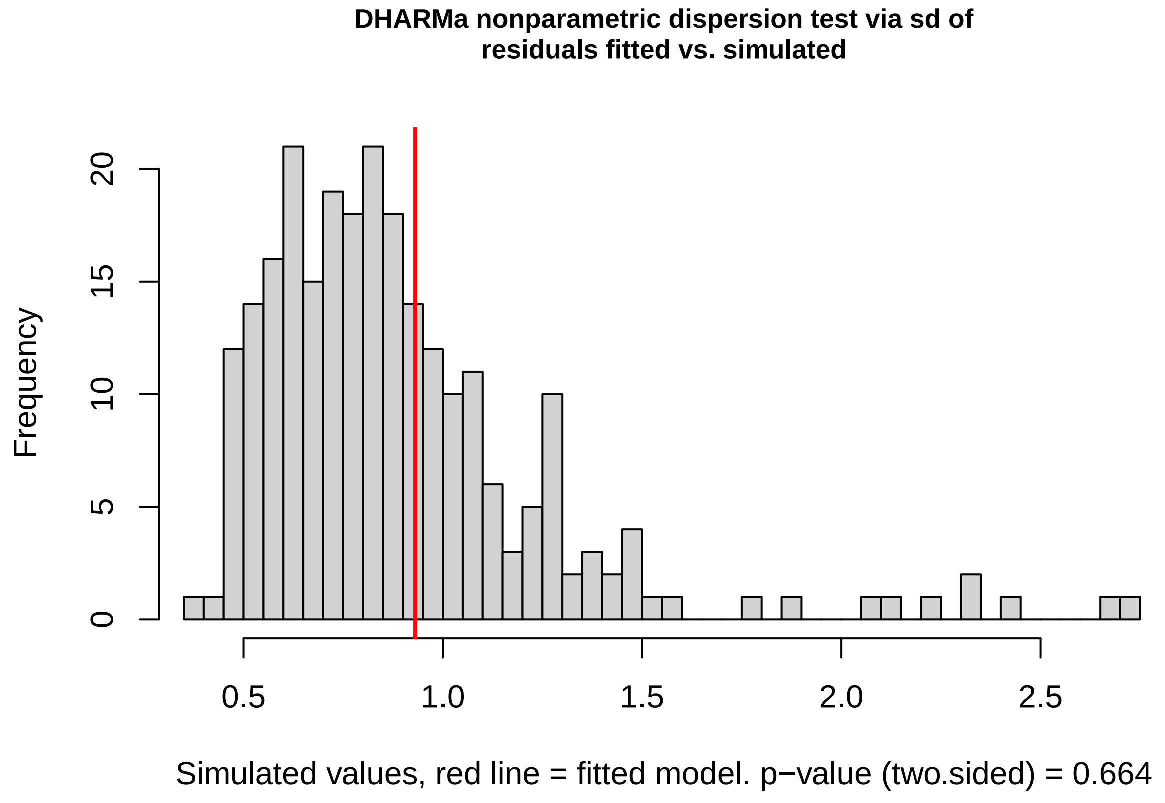

```
##
##  DHARMa nonparametric dispersion test via sd of residuals fitted vs.
##  simulated
##
## data:  simulationOutput
## dispersion = 1.0376, p-value = 0.664
## alternative hypothesis: two.sided
```

```
simulationOutput <- simulateResiduals(fittedModel = m5, plot = F)
residuals(simulationOutput)
```

```
##  [1] 0.456 0.952 0.928 0.812 0.272 0.980 0.976 0.752 0.964 0.448 0.208 0.744
## [13] 0.288 0.436 0.720 0.876 0.484 0.720 0.616 0.516 0.728 0.652 0.556 0.512
## [25] 0.440 0.540 0.696 0.996 0.972 0.600 0.184 0.604 0.348 0.764 0.696 0.496
## [37] 0.648 0.364 0.164 0.212 0.164 0.600 0.312 0.160 0.196 0.220 0.440 0.312
## [49] 0.096 0.048 0.120 0.088 0.088 0.388 0.176 0.492 0.492 0.088 0.100 0.148
## [61] 0.028 0.056 0.120 0.760 0.312 0.300 0.592 0.352 0.088 0.652 0.076 0.892
## [73] 0.144 0.072 0.184 0.324 0.216 0.344 0.156 0.844 0.876 0.168 0.468
```

```
residuals(simulationOutput, quantileFunction = qnorm, outlierValues = c(-7,7))
```

```
##  [1] -0.11051620  1.66456286  1.46105627  0.88529045 -0.60677536  2.05374891
##  [7]  1.97736843  0.68079692  1.79911811 -0.13071597 -0.81338039  0.65572668
## [13] -0.55923698 -0.16111859  0.58284151  1.15522085 -0.04011681  0.58284151
## [19]  0.29499199  0.04011681  0.60677536  0.39072570  0.14083537  0.03008408
## [25] -0.15096922  0.10043372  0.51293041  2.65206981  1.91103565  0.25334710
## [31] -0.90022599  0.26371440 -0.39072570  0.71922873  0.51293041 -0.01002668
## [37]  0.37992647 -0.34778720 -0.97815029 -0.79950094 -0.97815029  0.25334710
## [43] -0.49018923 -0.99445788 -0.85599599 -0.77219321 -0.15096922 -0.49018923
## [49] -1.30468539 -1.66456286 -1.17498679 -1.35317415 -1.35317415 -0.28453554
## [55] -0.93071695 -0.02005437 -0.02005437 -1.35317415 -1.28155157 -1.04504970
## [61] -1.91103565 -1.58926756 -1.17498679  0.70630256 -0.49018923 -0.52440051
## [67]  0.23269275 -0.37992647 -1.35317415  0.39072570 -1.43250272  1.23723460
## [73] -1.06251930 -1.46105627 -0.90022599 -0.45654238 -0.78577383 -0.40157070
## [79] -1.01103433  1.01103433  1.15522085 -0.96209875 -0.08029831
```

```
plot(simulationOutput)
```


Biology Open • Supplementary information

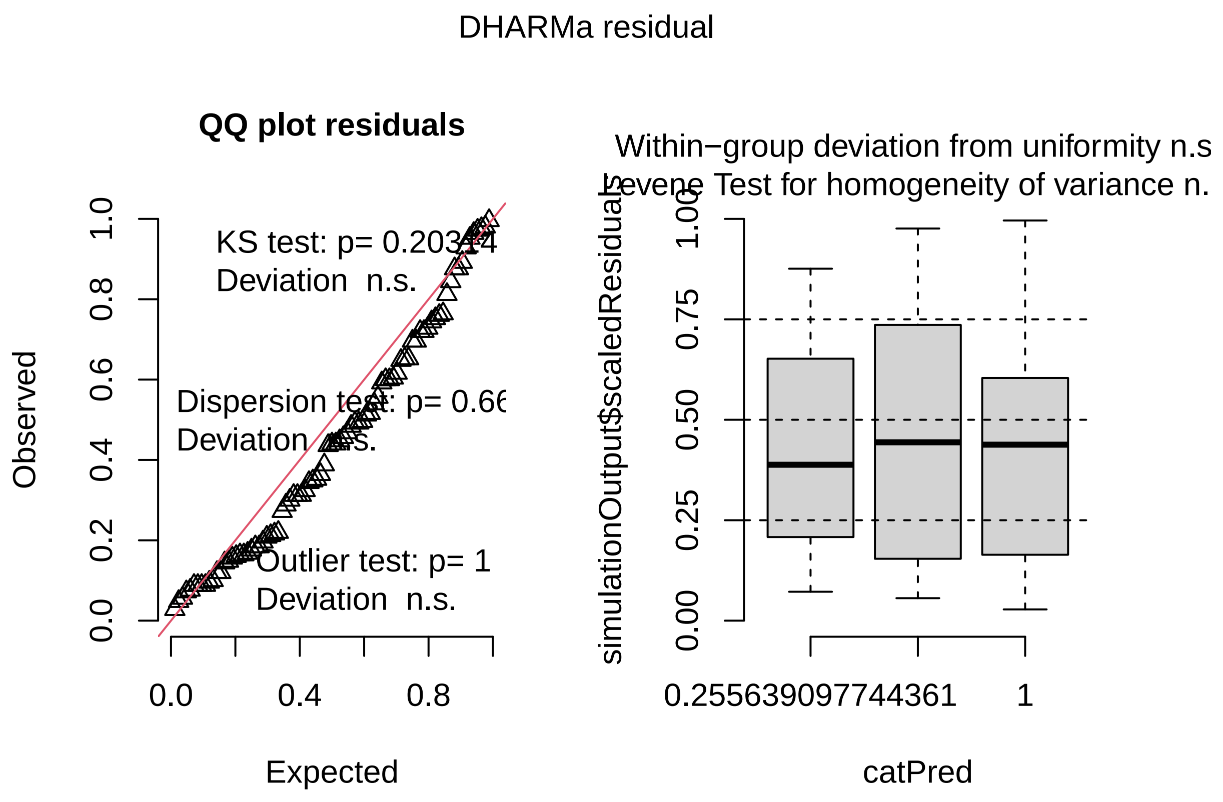


```r
# non-parametric alternative for mass effect
# Correlations
cor.test(Df5$mass_larvae_before, Df5$days_to_hatching, method= "spearman")
```

Correlations

```
## Warning in cor.test.default(Df5$mass_larvae_before, Df5$days_to_hatching, :
## Cannot compute exact p-value with ties

##
##  Spearman's rank correlation rho
##
## data:  Df5$mass_larvae_before and Df5$days_to_hatching
## S = 106467, p-value = 0.2907
## alternative hypothesis: true rho is not equal to 0
## sample estimates:
##        rho
## -0.1173633
```

```r
cor.test(Df5$mass_larvae_before, Df5$days_to_hatching, method= "kendall")
```

```
##
```

```
##  Kendall's rank correlation tau
##
## data:  Df5$mass_larvae_before and Df5$days_to_hatching
## z = -1.1256, p-value = 0.2603
## alternative hypothesis: true tau is not equal to 0
## sample estimates:
##         tau
## -0.08882004
```

```
# non-parametric alternative for relative body weight gain effect
cor.test(Df5$rel_BWG_to_pupation, Df5$days_to_hatching, method = "spearman")
```

```
## Warning in cor.test.default(Df5$rel_BWG_to_pupation, Df5$days_to_hatching, :
## Cannot compute exact p-value with ties
```

```
##
##  Spearman's rank correlation rho
##
## data:  Df5$rel_BWG_to_pupation and Df5$days_to_hatching
## S = 34976, p-value = 1.365e-10
## alternative hypothesis: true rho is not equal to 0
## sample estimates:
##       rho
## 0.6329303
```

```
cor.test(Df5$rel_BWG_to_pupation, Df5$days_to_hatching, method = "kendall")
```

```
##
##  Kendall's rank correlation tau
##
## data:  Df5$rel_BWG_to_pupation and Df5$days_to_hatching
## z = 6.36, p-value = 2.018e-10
## alternative hypothesis: true tau is not equal to 0
## sample estimates:
##       tau
## 0.5017014
```

Figure 1bd

```
# Figure 1b ----
treatment_order2 <- c("38°C", "37°C","Ctrl")

Df4 <- Df4 %>%
  mutate(treatment = factor(treatment, levels = treatment_order2))

sample_sizes4 <- Df4 %>%
  group_by(treatment) %>%
  summarise(n = n())
sample_sizes4
```


Biology Open • Supplementary information

```
## # A tibble: 3 x 2
##   treatment     n
##   <fct>     <int>
## 1 38°C         51
## 2 37°C         64
## 3 Ctrl         69
```

```
Fig1b <- Df4 %>%
  mutate(gr = interaction(days_to_pupation, treatment)) %>%
  ggplot(aes(x = treatment, y = days_to_pupation, fill = treatment)) +
  scale_fill_manual(values = c(alpha("#D55E00", 1),
                               alpha("#E69F00", 1),
                               alpha("#0072B2", 1))) +
  geom_violin() +
  #geom_jitter(size=1, alpha = 0.2, width = 0.1) +
  scale_y_continuous(breaks =  c(0, 5, 10, 15, 20, 25), limits = c(0, 25),
                     expand = expansion(mult = c(0.0, 0.05))) +
  geom_text(data = sample_sizes4, aes(x = treatment, y = 3.5, label = paste0("n=", n)),
            size = 3, vjust = 0.4 , hjust = 0, color = "white") +
  labs(title = "(b)",
       x = "Treatment",
       y = "Days to pupation") +
  coord_flip()+
  theme_classic() +
  theme(
    plot.title = element_text(hjust = 0, vjust = 1),
    text = element_text(size = 10),
    legend.position = "none",
    plot.margin = margin(0, 1, 0, 0, "cm")
  )
# Figure 1d ----
Df5 <- Df5 %>%
  mutate(treatment = factor(treatment, levels = treatment_order2))

sample_sizes5 <- Df5 %>%
  group_by(treatment) %>%
  summarise(n = n())
sample_sizes5
```

```
## # A tibble: 3 x 2
##   treatment     n
##   <fct>     <int>
## 1 38°C         16
## 2 37°C         33
## 3 Ctrl         34
```

```
Fig1d <- Df5 %>%
  mutate(gr = interaction(days_to_hatching, treatment)) %>%
  ggplot(aes(x = treatment, y = days_to_hatching, fill = treatment)) +
  scale_fill_manual(values = c(alpha("#D55E00", 1),
                               alpha("#E69F00", 1),
                               alpha("#0072B2", 1))) +
  geom_violin() +
```


Biology Open • Supplementary information

```
  #geom_jitter(size=1, alpha = 0.2, width = 0.1) +
  scale_y_continuous(breaks =  c(0, 5, 10, 15, 20, 25), limits = c(0, 25),
                     expand = expansion(mult = c(0.0, 0.05))) +
  geom_text(data = sample_sizes5, aes(x = treatment, y = 12, label = paste0("n=", n)),
            size = 3, vjust = 0.4 , hjust = 0.5, color = "white") +
  labs(title = "(d)",
       x = "Treatment",
       y = "Days to emergence") +
  coord_flip()+
  theme_classic() +
  theme(
    plot.title = element_text(hjust = 0, vjust = 1),
    text = element_text(size = 10),
    legend.position = "none",
    plot.margin = margin(0, 1, 0, 0, "cm")
  )
```

Arrangement of Figure 1

```
Fig1 <- grid.arrange(
  arrangeGrob(Fig1a),
  arrangeGrob(Fig1b),
  arrangeGrob(Fig1c),
  arrangeGrob(Fig1d),
  layout_matrix = matrix(c(1, 2, 2,
                           3, 4, 4),
                         nrow = 2, byrow = TRUE)

)
```


Biology Open • Supplementary information

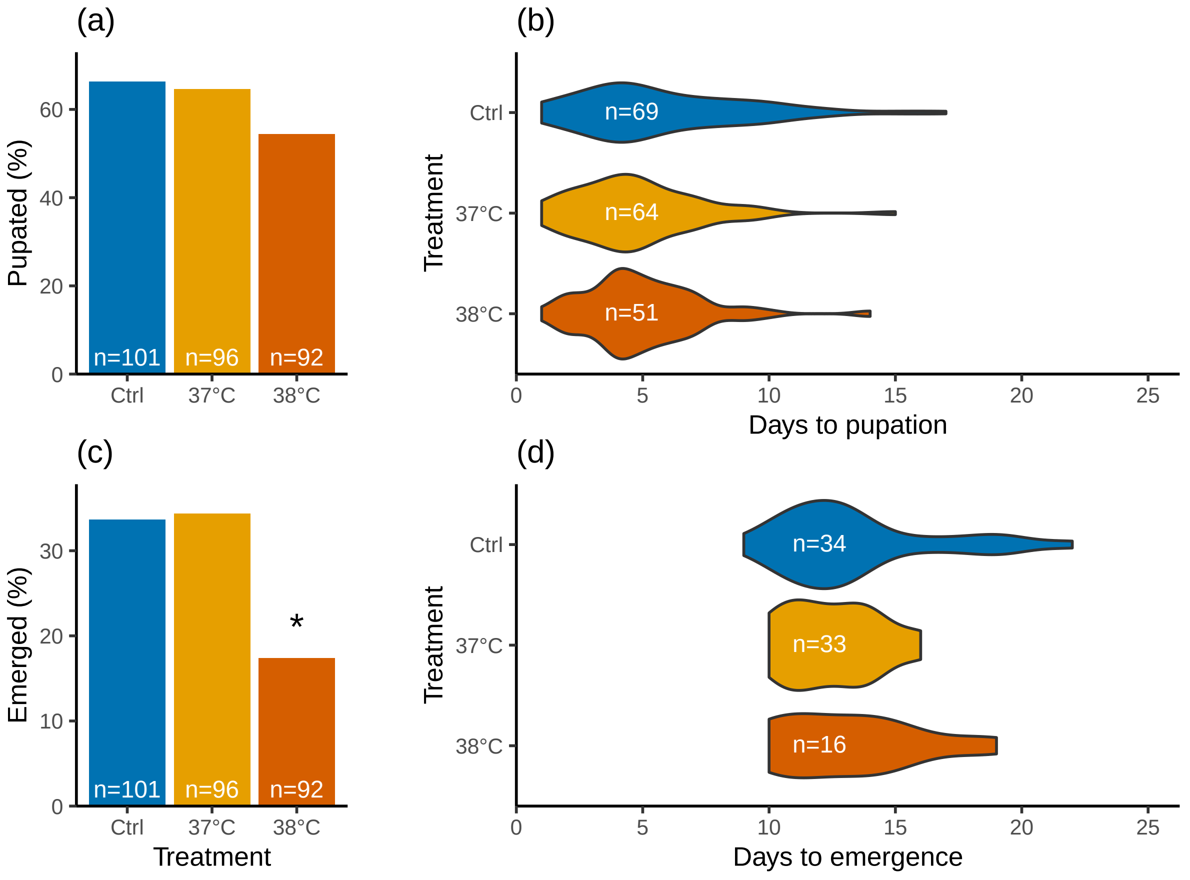


```
Fig1
```

```
## TableGrob (2 x 3) "arrange": 4 grobs
##   z     cells    name           grob
## 1 1 (1-1,1-1) arrange gtable[arrange]
## 2 2 (1-1,2-3) arrange gtable[arrange]
## 3 3 (2-2,1-1) arrange gtable[arrange]
## 4 4 (2-2,2-3) arrange gtable[arrange]
```

```
ggsave(Fig1, filename = "Fig1.png", width = 6, height = 4, dpi = 300, units = "in", device='png')
```

```
Df1 <- Df[!is.na(Df$rel_BWG_treatment), ] # Remove rows with missing values only for the column "BWG_treatment"
MassGainTreat <- summarySE(Df1, measurevar="rel_BWG_treatment", groupvars=c("treatment"))
MassGainTreat
```

Analysis of relative body weight gain during treatment

```
##   treatment  N rel_BWG_treatment       sd       se       ci
## 1      Ctrl 75       10.68058521 22.19146 2.562449 5.105790
## 2      37°C 74        4.90606457 21.75604 2.529088 5.040465
## 3      38°C 63       -0.06353202 22.92633 2.888446 5.773921
```


Biology Open • Supplementary information

```r
# NULL MODEL
m0 <- glmmTMB(
  rel_BWG_treatment ~ 1 + (1|colony),
  family = "gaussian", Df1)
# FULL MODEL
m1 <- glmmTMB(
  rel_BWG_treatment ~ treatment + (1|colony),
  family = "gaussian", Df1)
summary(m1)
```

```
##  Family: gaussian  ( identity )
## Formula:          rel_BWG_treatment ~ treatment + (1 | colony)
## Data: Df1
##
##      AIC      BIC   logLik deviance df.resid
##   1913.6   1930.4   -951.8   1903.6      207
##
## Random effects:
##
## Conditional model:
##  Groups   Name        Variance Std.Dev.
##  colony   (Intercept)  38.8     6.229
##  Residual             448.3    21.174
## Number of obs: 212, groups:  colony, 5
##
## Dispersion estimate for gaussian family (sigma^2):  448
##
## Conditional model:
##               Estimate Std. Error z value Pr(>|z|)
## (Intercept)     10.725      3.729   2.876  0.00402 **
## treatment37°C   -5.694      3.528  -1.614  0.10652
## treatment38°C  -10.217      3.647  -2.802  0.00508 **
## ---
## Signif. codes:  0 '***' 0.001 '**' 0.01 '*' 0.05 '.' 0.1 ' ' 1
```

```r
Anova(m1)
```

```
## Analysis of Deviance Table (Type II Wald chisquare tests)
##
## Response: rel_BWG_treatment
##            Chisq Df Pr(>Chisq)
## treatment 7.9381  2    0.01889 *
## ---
## Signif. codes:  0 '***' 0.001 '**' 0.01 '*' 0.05 '.' 0.1 ' ' 1
```

```r
### Post-hoc tests
means1 <- emmeans(m1, ~ treatment) # Obtain marginal means
posthoc_1 <- pairs(means1, adjust="Tukey")
posthoc_1
```

```
##  contrast    estimate   SE  df t.ratio p.value
##  Ctrl - 37°C     5.69 3.53 207   1.614  0.2420
```


Biology Open • Supplementary information

```
##  Ctrl - 38°C    10.22 3.65 207   2.802  0.0153
##  37°C - 38°C     4.52 3.65 207   1.239  0.4314
##
## P value adjustment: tukey method for comparing a family of 3 estimates
```

```
# Lower AIC or BIC values indicate better-fitting models
AIC(m0, m1)
```

```
##    df      AIC
## m0  3 1917.422
## m1  5 1913.623
```

```
BIC(m0, m1)
```

```
##    df      BIC
## m0  3 1927.492
## m1  5 1930.406
```

```
# Compared to NULL model
lrtest(m0, m1)
```

```
## Likelihood ratio test
##
## Model 1: rel_BWG_treatment ~ 1 + (1 | colony)
## Model 2: rel_BWG_treatment ~ treatment + (1 | colony)
##   #Df  LogLik Df  Chisq Pr(>Chisq)
## 1   3 -955.71
## 2   5 -951.81  2 7.7984    0.02026 *
## ---
## Signif. codes:  0 '***' 0.001 '**' 0.01 '*' 0.05 '.' 0.1 ' ' 1
```

```
anova(m0, m1)
```

```
## Data: Df1
## Models:
## m0: rel_BWG_treatment ~ 1 + (1 | colony), zi=~0, disp=~1
## m1: rel_BWG_treatment ~ treatment + (1 | colony), zi=~0, disp=~1
##    Df    AIC    BIC  logLik deviance  Chisq Chi Df Pr(>Chisq)
## m0  3 1917.4 1927.5 -955.71   1911.4
## m1  5 1913.6 1930.4 -951.81   1903.6 7.7984      2    0.02026 *
## ---
## Signif. codes:  0 '***' 0.001 '**' 0.01 '*' 0.05 '.' 0.1 ' ' 1
```

```
# Model diagnostics
testDispersion(m1)
```


Biology Open • Supplementary information

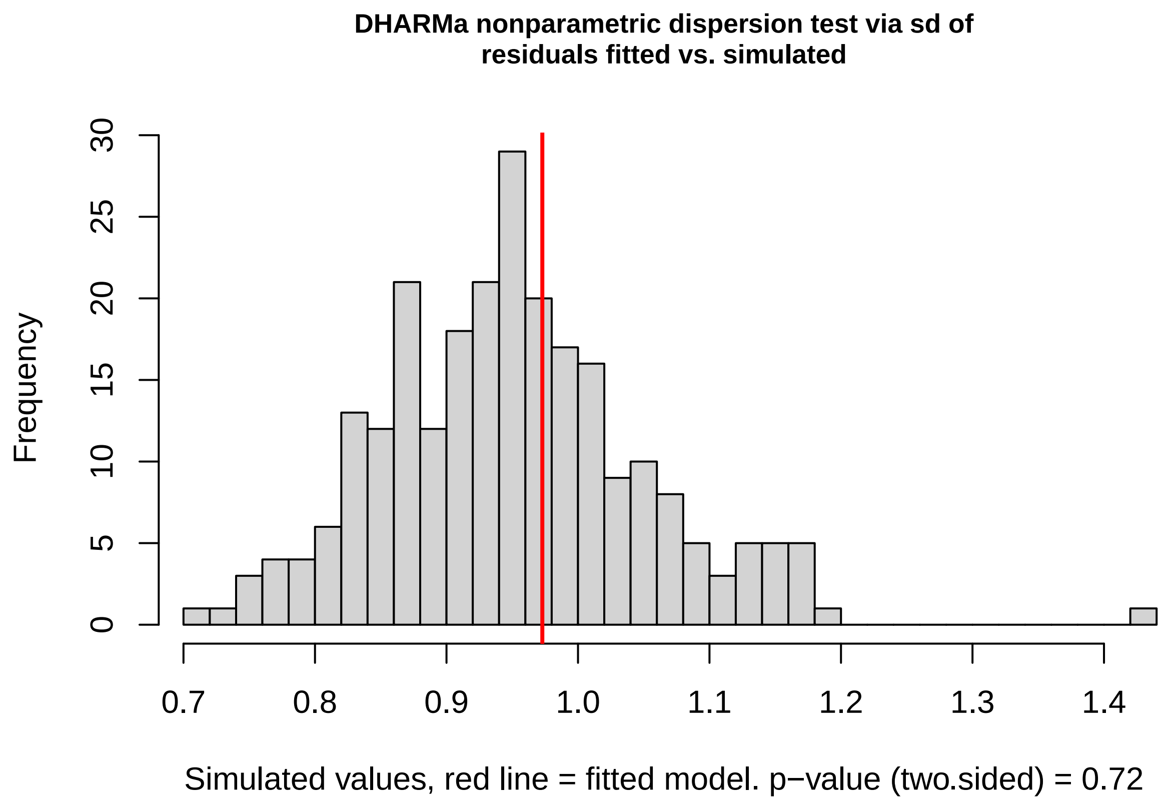


```
##
##  DHARMa nonparametric dispersion test via sd of residuals fitted vs.
##  simulated
##
## data:  simulationOutput
## dispersion = 1.0241, p-value = 0.72
## alternative hypothesis: two.sided
```

```
simulationOutput <- simulateResiduals(fittedModel = m1, plot = F)
residuals(simulationOutput)
```

```
##   [1] 0.660 0.688 0.668 0.468 0.528 0.876 0.868 0.720 0.940 0.740 0.384 0.636
##  [13] 0.484 0.676 0.416 0.336 0.148 0.104 0.704 0.244 0.692 0.516 0.708 0.300
##  [25] 0.348 0.608 0.944 0.952 0.120 0.812 0.860 0.264 0.896 0.008 0.248 0.496
##  [37] 0.348 0.828 0.132 0.796 0.584 0.536 0.528 0.916 0.120 0.612 0.844 0.664
##  [49] 0.792 0.704 0.856 0.236 0.628 0.608 0.896 0.156 0.400 0.556 0.744 0.480
##  [61] 0.720 0.464 0.128 0.720 0.820 0.272 0.340 0.548 0.436 0.516 0.468 0.272
##  [73] 0.092 0.920 0.152 0.800 0.876 0.552 0.804 0.868 0.828 0.832 0.116 0.452
##  [85] 0.652 0.416 1.000 0.164 0.180 0.520 0.620 0.416 0.968 0.384 0.512 0.588
##  [97] 0.284 0.244 0.960 0.716 0.884 0.224 0.656 0.852 0.148 1.000 0.068 0.232
## [109] 0.608 0.984 0.736 0.280 0.124 0.644 0.240 0.996 0.932 0.724 0.088 0.128
## [121] 0.704 0.188 0.120 0.476 0.072 0.076 0.092 0.140 0.152 0.076 0.344 0.120
## [133] 0.200 0.792 0.556 0.524 0.376 0.896 0.196 0.596 0.772 0.992 0.844 0.328
## [145] 0.720 0.656 0.160 0.120 0.184 0.108 0.164 0.120 0.960 0.660 0.284 0.464
## [157] 0.272 0.192 0.420 0.716 0.620 0.248 0.528 0.340 0.200 0.448 0.064 0.084
```


Biology Open • Supplementary information

```
## [169] 0.716 0.544 0.556 0.868 0.120 0.132 0.292 0.124 0.608 0.064 0.200 0.108
## [181] 0.832 0.444 0.708 0.704 0.104 0.272 0.548 0.800 0.384 0.116 0.500 0.040
## [193] 0.288 0.300 0.124 0.036 0.092 0.056 0.160 0.168 0.128 0.572 0.500 0.916
## [205] 0.572 0.576 0.636 0.736 0.076 0.188 0.992 0.996
```

```
residuals(simulationOutput, quantileFunction = qnorm, outlierValues = c(-7,7))
```

```
##   [1]  0.41246313  0.49018923  0.43439724 -0.08029831  0.07024331  1.15522085
##   [7]  1.11698673  0.58284151  1.55477359  0.64334541 -0.29499199  0.34778720
##  [13] -0.04011681  0.45654238 -0.21213720 -0.42340472 -1.04504970 -1.25908398
##  [19]  0.53594003 -0.69349335  0.50152740  0.04011681  0.54755135 -0.52440051
##  [25] -0.39072570  0.27411012  1.58926756  1.66456286 -1.17498679  0.88529045
##  [31]  1.08031934 -0.63106198  1.25908398 -2.40891555 -0.68079692 -0.01002668
##  [37] -0.39072570  0.94629136 -1.11698673  0.82741832  0.21213720  0.09036144
##  [43]  0.07024331  1.37865873 -1.17498679  0.28453554  1.01103433  0.42340472
##  [49]  0.81338039  0.53594003  1.06251930 -0.71922873  0.32656093  0.27411012
##  [55]  1.25908398 -1.01103433 -0.25334710  0.14083537  0.65572668 -0.05015358
##  [61]  0.58284151 -0.09036144 -1.13589622  0.58284151  0.91536509 -0.60677536
##  [67] -0.41246313  0.12060993 -0.16111859  0.04011681 -0.08029831 -0.60677536
##  [73] -1.32853933  1.40507156 -1.02789335  0.84162123  1.15522085  0.13071597
##  [79]  0.85599599  1.11698673  0.94629136  0.96209875 -1.19522278 -0.12060993
##  [85]  0.39072570 -0.21213720  7.00000000 -0.97815029 -0.91536509  0.05015358
##  [91]  0.30548079 -0.21213720  1.85217986 -0.29499199  0.03008408  0.22240323
##  [97] -0.57099947 -0.69349335  1.75068607  0.57099947  1.19522278 -0.75875354
## [103]  0.40157070  1.04504970 -1.04504970  7.00000000 -1.49085336 -0.73227620
## [109]  0.27411012  2.14441062  0.63106198 -0.58284151 -1.15522085  0.36917136
## [115] -0.70630256  2.65206981  1.49085336  0.59476585 -1.35317415 -1.13589622
## [121]  0.53594003 -0.88529045 -1.17498679 -0.06019541 -1.46105627 -1.43250272
## [127] -1.32853933 -1.08031934 -1.02789335 -1.43250272 -0.40157070 -1.17498679
## [133] -0.84162123  0.81338039  0.14083537  0.06019541 -0.31600330  1.25908398
## [139] -0.85599599  0.24300697  0.74544955  2.40891555  1.01103433 -0.44544251
## [145]  0.58284151  0.40157070 -0.99445788 -1.17498679 -0.90022599 -1.23723460
## [151] -0.97815029 -1.17498679  1.75068607  0.41246313 -0.57099947 -0.09036144
## [157] -0.60677536 -0.87054983 -0.20189348  0.57099947  0.30548079 -0.68079692
## [163]  0.07024331 -0.41246313 -0.84162123 -0.13071597 -1.52203624 -1.37865873
## [169]  0.57099947  0.11051620  0.14083537  1.11698673 -1.17498679 -1.11698673
## [175] -0.54755135 -1.15522085  0.27411012 -1.52203624 -0.84162123 -1.23723460
## [181]  0.96209875 -0.14083537  0.54755135  0.53594003 -1.25908398 -0.60677536
## [187]  0.12060993  0.84162123 -0.29499199 -1.19522278  0.00000000 -1.75068607
## [193] -0.55923698 -0.52440051 -1.15522085 -1.79911811 -1.32853933 -1.58926756
## [199] -0.99445788 -0.96209875 -1.13589622  0.18146832  0.00000000  1.37865873
## [205]  0.18146832  0.19167090  0.34778720  0.63106198 -1.43250272 -0.88529045
## [211]  2.40891555  2.65206981
```

```
plot(simulationOutput)
```


Biology Open • Supplementary information

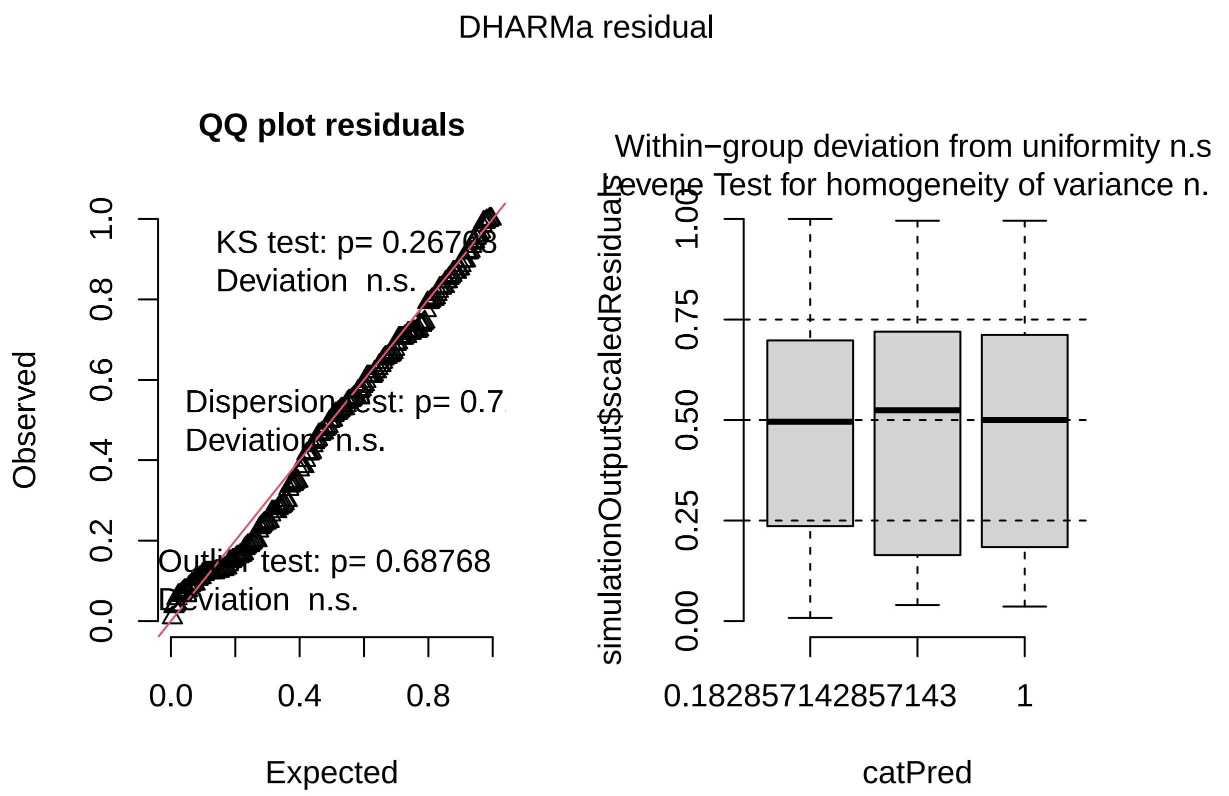


```
Df3 <- Df[!is.na(Df$rel_BWG_during_pupation), ]
MassGainHatch <- summarySE(Df3, measurevar="rel_BWG_during_pupation", groupvars=c("treatment"))
MassGainHatch
```

Analysis of relative body weight loss during pupation

```
##   treatment  N rel_BWG_during_pupation       sd       se       ci
## 1      Ctrl 34               -51.78433 6.542355 1.122005 2.282736
## 2      37°C 33               -51.33760 6.518598 1.134742 2.311394
## 3      38°C 16               -51.98395 5.397937 1.349484 2.876358
```

```
# NULL MODEL
m0c <- glmmTMB(
  rel_BWG_during_pupation ~ 1 + (1|colony),
  family = "gaussian", Df1)
# Full MODEL
m3 <- glmmTMB(
  rel_BWG_during_pupation ~ treatment + (1|colony),
  family = "gaussian", Df1)
summary(m3)
```

```
##  Family: gaussian  ( identity )
```

```
## Formula:          rel_BWG_during_pupation ~ treatment + (1 | colony)
## Data: Df1
##
##      AIC      BIC   logLik deviance df.resid
##    545.7    557.8   -267.8    535.7       78
##
## Random effects:
##
## Conditional model:
##  Groups   Name        Variance Std.Dev.
##  colony   (Intercept)  3.622   1.903
##  Residual             35.133   5.927
## Number of obs: 83, groups:  colony, 5
##
## Dispersion estimate for gaussian family (sigma^2): 35.1
##
## Conditional model:
##               Estimate Std. Error z value Pr(>|z|)
## (Intercept)   -52.2155     1.3711  -38.08   <2e-16 ***
## treatment37°C   0.8341     1.5037    0.55    0.579
## treatment38°C  -0.5263     1.8153   -0.29    0.772
## ---
## Signif. codes:  0 '***' 0.001 '**' 0.01 '*' 0.05 '.' 0.1 ' ' 1
```

```
Anova(m3)
```

```
## Analysis of Deviance Table (Type II Wald chisquare tests)
##
## Response: rel_BWG_during_pupation
##            Chisq Df Pr(>Chisq)
## treatment 0.5995  2      0.741
```

```
### Post-hoc tests
means3 <- emmeans(m3, ~ treatment) # Obtain marginal means
posthoc3 <- pairs(means3, adjust= "Tukey")
posthoc3
```

```
##  contrast    estimate   SE df t.ratio p.value
##  Ctrl - 37°C   -0.834 1.50 78  -0.555  0.8444
##  Ctrl - 38°C    0.526 1.82 78   0.290  0.9548
##  37°C - 38°C    1.360 1.87 78   0.728  0.7475
##
## P value adjustment: tukey method for comparing a family of 3 estimates
```

```
# Lower AIC or BIC values indicate better-fitting models
AIC(m0c, m3)
```

```
##     df      AIC
## m0c  3 542.2677
## m3   5 545.6716
```


Biology Open • Supplementary information

```
BIC(m0c, m3)
```

```
##     df      BIC
## m0c  3 549.5242
## m3   5 557.7658
```

```
# Compared to NULL model
lrtest(m0c, m3)
```

```
## Likelihood ratio test
##
## Model 1: rel_BWG_during_pupation ~ 1 + (1 | colony)
## Model 2: rel_BWG_during_pupation ~ treatment + (1 | colony)
##   #Df  LogLik Df Chisq Pr(>Chisq)
## 1   3 -268.13
## 2   5 -267.84  2 0.596     0.7423
```

```
anova(m0c, m3)
```

```
## Data: Df1
## Models:
## m0c: rel_BWG_during_pupation ~ 1 + (1 | colony), zi=~0, disp=~1
## m3: rel_BWG_during_pupation ~ treatment + (1 | colony), zi=~0, disp=~1
##     Df    AIC    BIC  logLik deviance Chisq Chi Df Pr(>Chisq)
## m0c  3 542.27 549.52 -268.13   536.27
## m3   5 545.67 557.77 -267.84   535.67 0.596      2     0.7423
```

```
# Model diagnostics
testDispersion(m3)
```

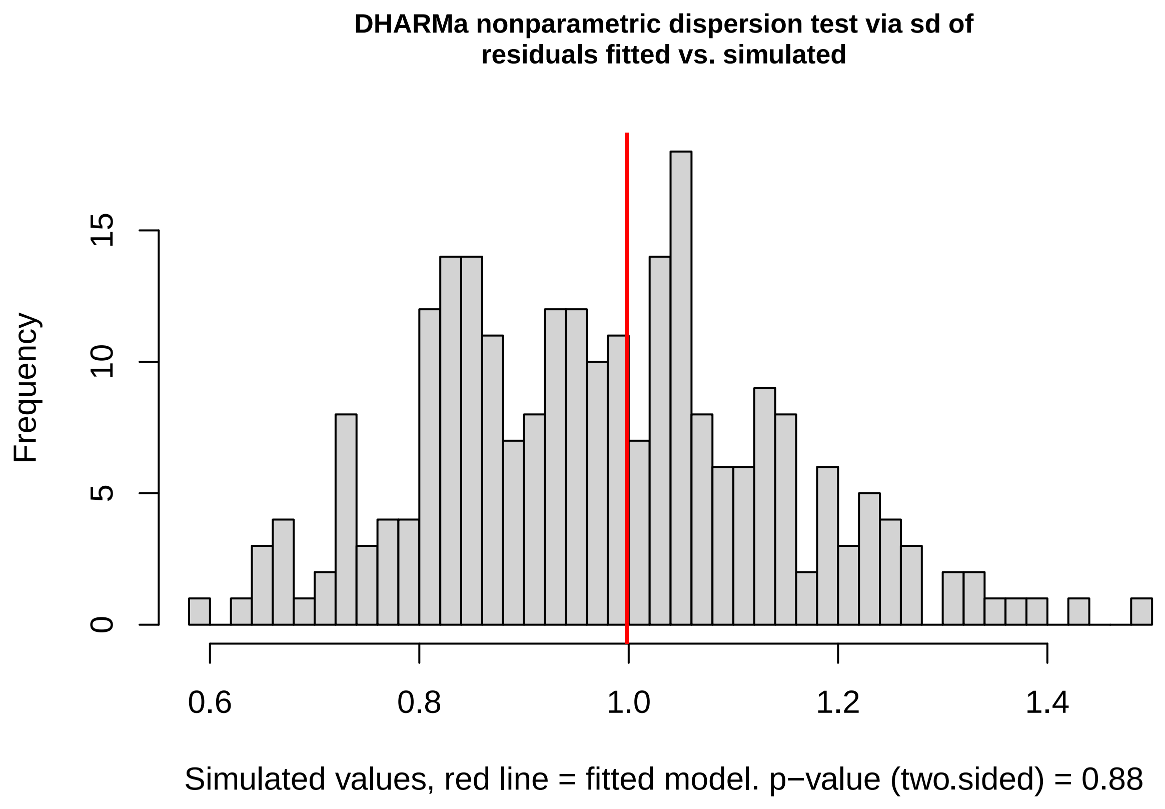


```
##
##  DHARMa nonparametric dispersion test via sd of residuals fitted vs.
##  simulated
##
## data:  simulationOutput
## dispersion = 1.0236, p-value = 0.88
## alternative hypothesis: two.sided
```

```
simulationOutput <- simulateResiduals(fittedModel = m3, plot = F)
residuals(simulationOutput)
```

```
##  [1] 0.180 0.112 0.184 0.272 0.556 0.256 0.280 0.604 0.028 0.800 0.820 0.824
## [13] 0.708 0.284 0.420 0.192 0.500 0.216 0.428 0.544 0.404 0.252 0.284 0.748
## [25] 0.436 0.232 0.392 0.012 0.200 0.336 0.976 0.796 0.548 0.160 0.292 0.388
## [37] 0.424 0.464 0.788 0.104 0.904 0.316 0.688 0.708 0.576 0.980 0.448 0.652
## [49] 0.960 0.348 0.716 0.220 0.312 0.640 0.864 0.432 0.744 0.820 0.768 0.796
## [61] 0.996 0.272 0.692 0.308 0.780 0.208 0.008 0.412 0.912 0.996 0.912 0.412
## [73] 0.680 0.880 0.884 0.060 0.668 0.020 0.772 0.404 0.168 0.972 0.484
```

```
residuals(simulationOutput, quantileFunction = qnorm, outlierValues = c(-7,7))
```

```
##  [1] -0.91536509 -1.21596042 -0.90022599 -0.60677536  0.14083537 -0.65572668
##  [7] -0.58284151  0.26371440 -1.91103565  0.84162123  0.91536509  0.93071695
## [13]  0.54755135 -0.57099947 -0.20189348 -0.87054983  0.00000000 -0.78577383
```


Biology Open • Supplementary information

```
## [19] -0.18146832  0.11051620 -0.24300697 -0.66820930 -0.57099947  0.66820930
## [25] -0.16111859 -0.73227620 -0.27411012 -2.25712924 -0.84162123 -0.42340472
## [31]  1.97736843  0.82741832  0.12060993 -0.99445788 -0.54755135 -0.28453554
## [37] -0.19167090 -0.09036144  0.79950094 -1.25908398  1.30468539 -0.47891373
## [43]  0.49018923  0.54755135  0.19167090  2.05374891 -0.13071597  0.39072570
## [49]  1.75068607 -0.39072570  0.57099947 -0.77219321 -0.49018923  0.35845879
## [55]  1.09846842 -0.17128459  0.65572668  0.91536509  0.73227620  0.82741832
## [61]  2.65206981 -0.60677536  0.50152740 -0.50152740  0.77219321 -0.81338039
## [67] -2.40891555 -0.22240323  1.35317415  2.65206981  1.35317415 -0.22240323
## [73]  0.46769880  1.17498679  1.19522278 -1.55477359  0.43439724 -2.05374891
## [79]  0.74544955 -0.24300697 -0.96209875  1.91103565 -0.04011681
```

```
plot(simulationOutput)
```

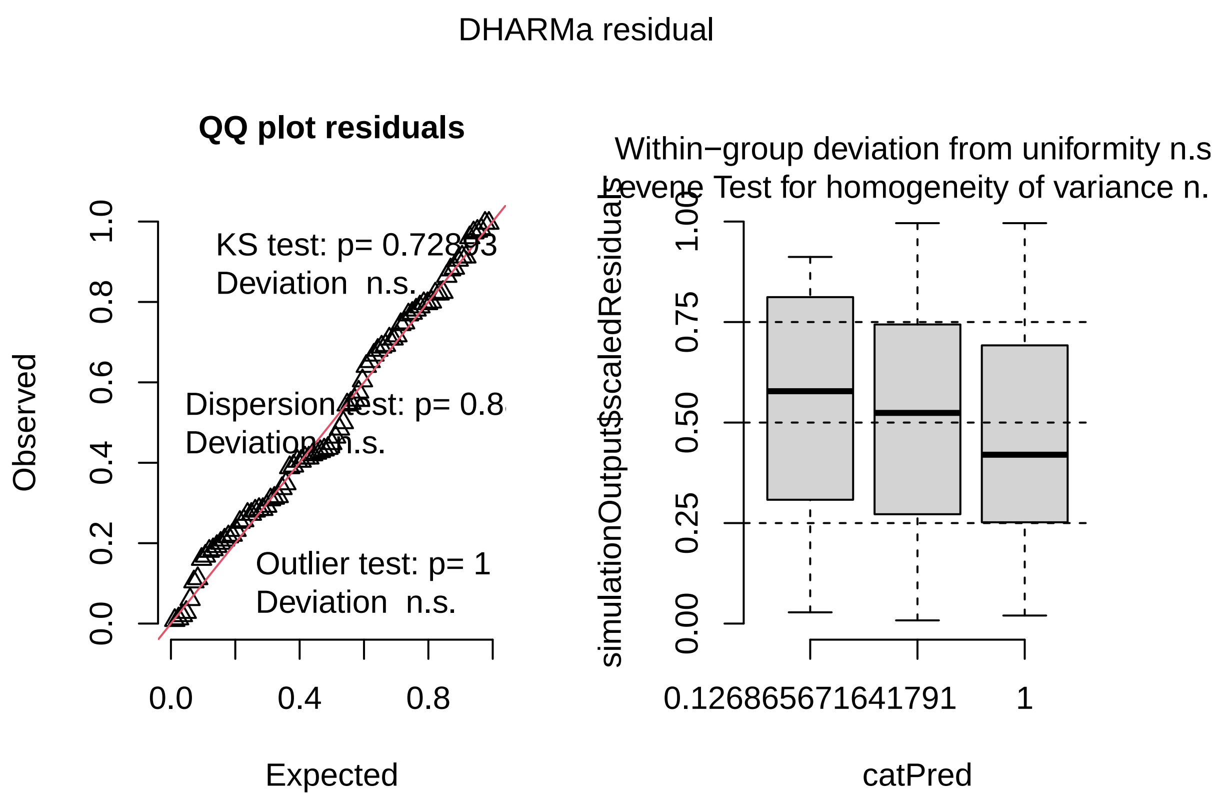


```
Df_Adults <- Df1 %>%
  filter(developmental_stage_reached == "Adult")

m0d2 <- glmmTMB(
  log10(dry_mass+1) ~ 1 + (1|colony) + (1|sex),
  family = Gamma(link = "log"), Df_Adults)
# FULL MODEL
m4b <- glmmTMB(
```


Biology Open • Supplementary information

```
  log10(dry_mass+1)  ~ treatment + (1|colony) + (1|sex),
  family = Gamma(link = "log"), Df_Adults)
summary(m4b)
```

Analysis of adult body size using dry mass as proxy

```
##  Family: Gamma  ( log )
## Formula:          log10(dry_mass + 1) ~ treatment + (1 | colony) + (1 | sex)
## Data: Df_Adults
##
##      AIC      BIC   logLik deviance df.resid
##   -705.1   -690.6    358.5   -717.1       77
##
## Random effects:
##
## Conditional model:
##  Groups Name        Variance Std.Dev.
##  colony (Intercept) 0.007847 0.08858
##  sex    (Intercept) 0.015585 0.12484
## Number of obs: 83, groups:  colony, 5; sex, 2
##
## Dispersion estimate for Gamma family (sigma^2): 0.0886
##
## Conditional model:
##               Estimate Std. Error z value Pr(>|z|)
## (Intercept)   -4.45451    0.11388  -39.12   <2e-16 ***
## treatment37°C -0.03786    0.07536   -0.50    0.615
## treatment38°C -0.13893    0.09253   -1.50    0.133
## ---
## Signif. codes:  0 '***' 0.001 '**' 0.01 '*' 0.05 '.' 0.1 ' ' 1
```

```
Anova(m4b)
```

```
## Analysis of Deviance Table (Type II Wald chisquare tests)
##
## Response: log10(dry_mass + 1)
##           Chisq Df Pr(>Chisq)
## treatment 2.264  2     0.3224
```

```
### Post-hoc tests
means4b <- emmeans(m4b, ~ treatment) # Obtain marginal means
posthoc_4b <- pairs(means4b, adjust="Tukey")
posthoc_4b
```

```
##  contrast    estimate     SE  df z.ratio p.value
##  Ctrl - 37°C   0.0379 0.0754 Inf   0.502  0.8702
##  Ctrl - 38°C   0.1389 0.0925 Inf   1.502  0.2902
##  37°C - 38°C   0.1011 0.0935 Inf   1.081  0.5261
##
## Note: contrasts are still on the log(mu + 1) scale. Consider using
##       regrid() if you want contrasts of back-transformed estimates.
## Results are given on the log10 (not the response) scale.
## P value adjustment: tukey method for comparing a family of 3 estimates
```


Biology Open • Supplementary information

```r
# Lower AIC or BIC values indicate better-fitting models
AIC(m0d2, m4b)
```

```
##      df       AIC
## m0d2  4 -706.8818
## m4b   6 -705.0637
```

```r
BIC(m0d2, m4b)
```

```
##      df       BIC
## m0d2  4 -697.2064
## m4b   6 -690.5506
```

```r
# Compared to NULL model
lrtest(m0d2, m4b)
```

```
## Likelihood ratio test
##
## Model 1: log10(dry_mass + 1) ~ 1 + (1 | colony) + (1 | sex)
## Model 2: log10(dry_mass + 1) ~ treatment + (1 | colony) + (1 | sex)
##   #Df LogLik Df  Chisq Pr(>Chisq)
## 1   4 357.44
## 2   6 358.53  2 2.1819     0.3359
```

```r
anova(m0d2, m4b)
```

```
## Data: Df_Adults
## Models:
## m0d2: log10(dry_mass + 1) ~ 1 + (1 | colony) + (1 | sex), zi=~0, disp=~1
## m4b: log10(dry_mass + 1) ~ treatment + (1 | colony) + (1 | sex), zi=~0, disp=~1
##      Df     AIC     BIC logLik deviance  Chisq Chi Df Pr(>Chisq)
## m0d2  4 -706.88 -697.21 357.44  -714.88
## m4b   6 -705.06 -690.55 358.53  -717.06 2.1819      2     0.3359
```

```r
# Model diagnostics
testDispersion(m4b)
```


Biology Open • Supplementary information

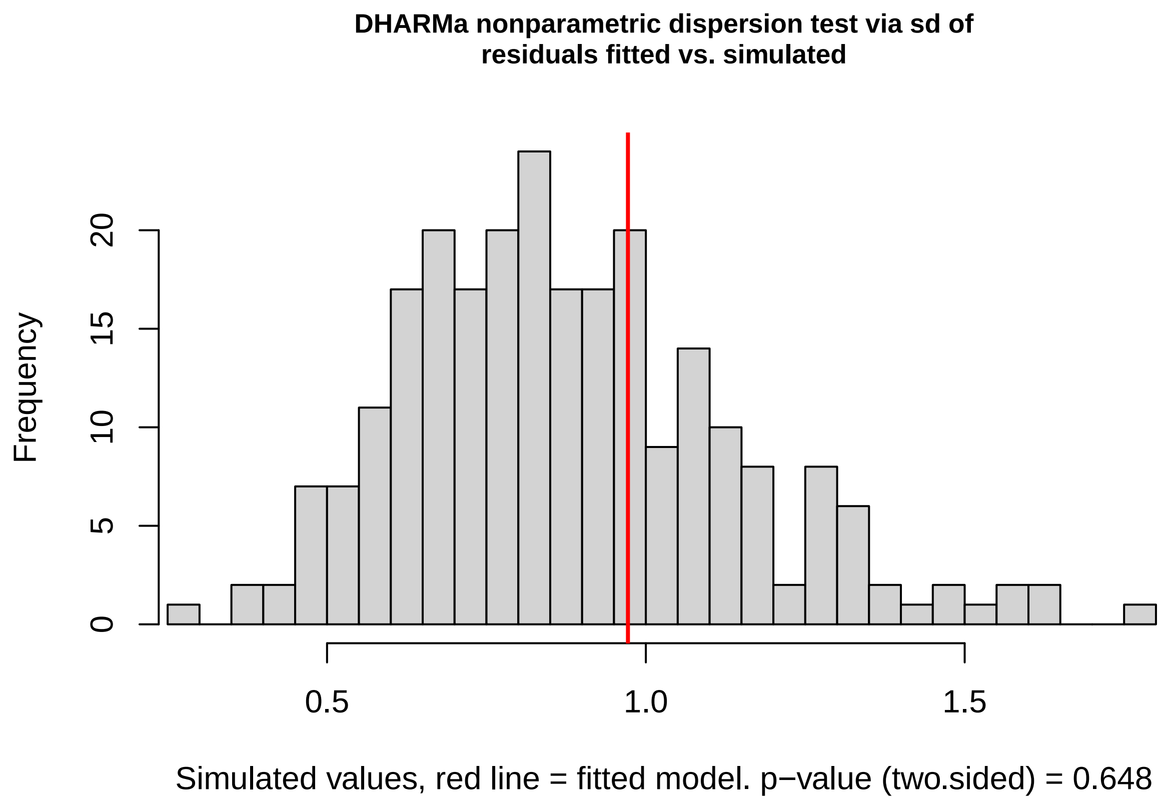


```
##
##  DHARMa nonparametric dispersion test via sd of residuals fitted vs.
##  simulated
##
## data:  simulationOutput
## dispersion = 1.1078, p-value = 0.648
## alternative hypothesis: two.sided
```

```
simulationOutput <- simulateResiduals(fittedModel = m4b, plot = F)
residuals(simulationOutput)
```

```
##  [1] 0.068 0.516 0.456 0.176 0.424 0.676 0.660 0.972 0.372 0.296 0.768 0.976
## [13] 0.216 0.656 0.240 0.468 0.348 0.512 0.460 0.712 0.428 0.168 0.092 0.484
## [25] 0.616 0.424 0.724 0.588 0.700 0.868 0.368 0.916 0.156 0.152 0.240 0.296
## [37] 0.348 0.440 0.884 0.656 0.440 0.264 0.284 0.556 0.152 0.688 0.252 0.876
## [49] 0.844 0.516 0.208 0.416 0.140 0.108 0.296 0.124 0.236 0.164 0.588 0.432
## [61] 0.668 0.412 0.104 1.000 0.156 0.024 0.016 0.056 0.484 0.932 0.728 0.612
## [73] 0.488 0.536 0.176 0.048 0.272 0.096 0.020 0.848 0.696 0.976 0.936
```

```
residuals(simulationOutput, quantileFunction = qnorm, outlierValues = c(-7,7))
```

```
##  [1] -1.49085336  0.04011681 -0.11051620 -0.93071695 -0.19167090  0.45654238
##  [7]  0.41246313  1.91103565 -0.32656093 -0.53594003  0.73227620  1.97736843
## [13] -0.78577383  0.40157070 -0.70630256 -0.08029831 -0.39072570  0.03008408
```


Biology Open • Supplementary information

```
## [19] -0.10043372  0.55923698 -0.18146832 -0.96209875 -1.32853933 -0.04011681
## [25]  0.29499199 -0.19167090  0.59476585  0.22240323  0.52440051  1.11698673
## [31] -0.33715508  1.37865873 -1.01103433 -1.02789335 -0.70630256 -0.53594003
## [37] -0.39072570 -0.15096922  1.19522278  0.40157070 -0.15096922 -0.63106198
## [43] -0.57099947  0.14083537 -1.02789335  0.49018923 -0.66820930  1.15522085
## [49]  1.01103433  0.04011681 -0.81338039 -0.21213720 -1.08031934 -1.23723460
## [55] -0.53594003 -1.15522085 -0.71922873 -0.97815029  0.22240323 -0.17128459
## [61]  0.43439724 -0.22240323 -1.25908398  7.00000000 -1.01103433 -1.97736843
## [67] -2.14441062 -1.58926756 -0.04011681  1.49085336  0.60677536  0.28453554
## [73] -0.03008408  0.09036144 -0.93071695 -1.66456286 -0.60677536 -1.30468539
## [79] -2.05374891  1.02789335  0.51293041  1.97736843  1.52203624
```

```
plot(simulationOutput)
```

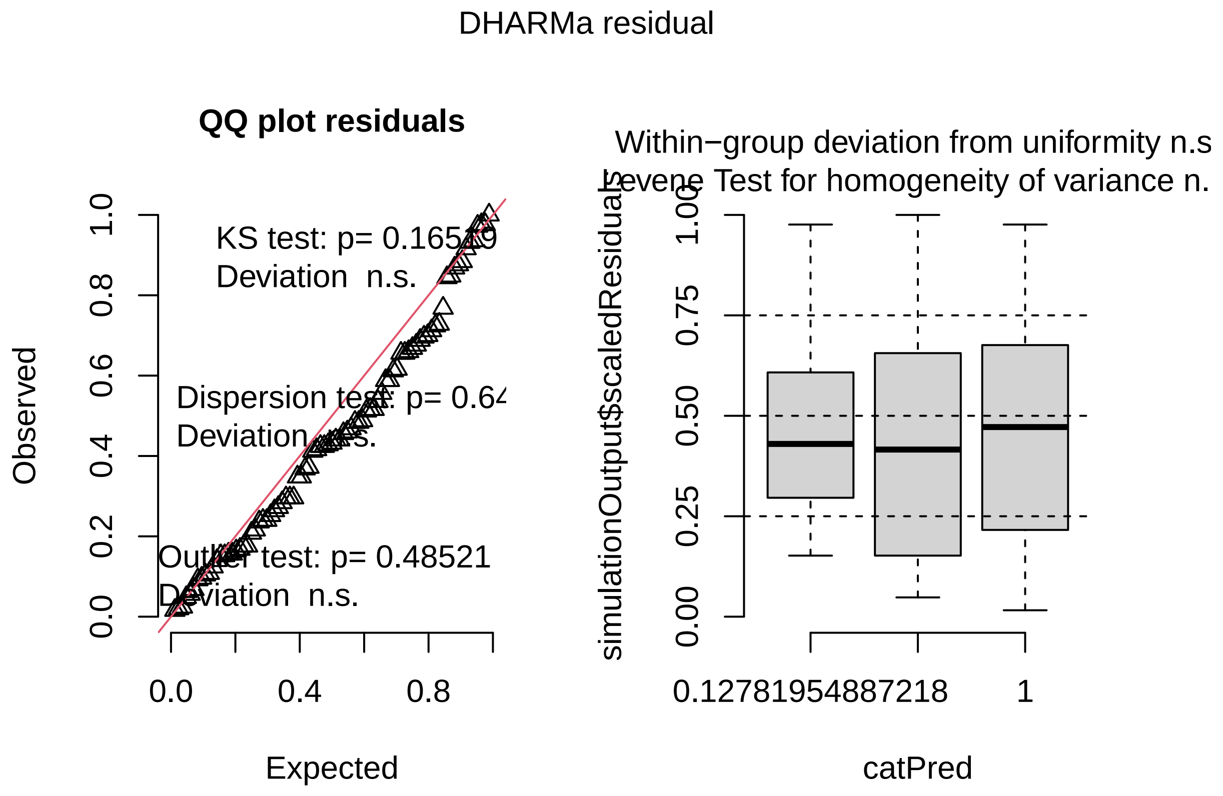


```
# non-parametric alternative
m4b <- kruskal.test(dry_mass ~ treatment, data = Df_Adults)
m4b
```

```
##
##  Kruskal-Wallis rank sum test
##
## data:  dry_mass by treatment
## Kruskal-Wallis chi-squared = 2.4566, df = 2, p-value = 0.2928
```


Biology Open • Supplementary information

Figure 2 top

```
# Figure 2a ----
sample_sizes1 <- Df1 %>%
  group_by(treatment) %>%
  summarise(n = n())
Fig2a <- Df1 %>%
  mutate(gr = interaction(rel_BWG_treatment, treatment)) %>%
  ggplot(aes(x = treatment, y = rel_BWG_treatment, fill = treatment)) +
  scale_fill_manual(values = c(alpha("#0072B2", 1),
                               alpha("#E69F00", 1),
                               alpha("#D55E00", 1))) +
  stat_summary(fun = mean, geom = "bar") +
  stat_summary(fun.data = "mean_se", geom = "errorbar", width = 0.1, alpha = 0.4) + # "mean_cl_normal" 95% CI
  geom_text(data = data.frame(x = c("37°C", "38°C"),
                              y = c(8, 3.3),
                              label = c("", "*")), # from post-hoc test
            aes(x = x, y = y, label = label), size = 5, color = "black", vjust = -0.5, inherit.aes = FALSE) +
  geom_text(data = sample_sizes1, aes(x = treatment, y = -6, label = paste0("n=", n)),
            size = 3, vjust = -0.5, hjust = 0.5, color = "grey") +
  scale_y_continuous(expand = expansion(mult = c(0.01, 0.12))) +
  #scale_y_continuous(expand = expansion(mult = c(0.1, 0.13))) +
  geom_hline(yintercept = 0, linetype = "dashed", color = "#666666") +
  labs(title = "(a) L4 larvae",
       x = "",
       y = "Relative body weight gain (%)") +
  theme_classic() +
  theme(
    plot.title = element_text(hjust = 0, vjust = 1),
    text = element_text(size = 10),
    legend.position = "none",
    plot.margin = margin(0, 1, 0, 0, "cm")
  )

# Figure 2b ----
sample_sizes3 <- Df3 %>%
  group_by(treatment) %>%
  summarise(n = n())
Fig2b <- Df3 %>%
  mutate(gr = interaction(rel_BWG_during_pupation, treatment)) %>%
  ggplot(aes(x = treatment, y = rel_BWG_during_pupation, fill = treatment)) +
  #geom_boxplot(alpha = 0.8)+
  #geom_jitter(aes(fill = treatment), size = 2, alpha = 0.2, width = 0.15) +
  stat_summary(fun = mean, geom = "bar") +
  stat_summary(fun.data = "mean_se", geom = "errorbar", width = 0.1, alpha = 0.4) +
  scale_y_continuous(expand = expansion(mult = c(0.05, 0.1)),
                     breaks =  c(0, -10, -20, -30, -40, -50)) +
  geom_text(data = sample_sizes3, aes(x = treatment, y = 0.5, label = paste0("n=", n)),
            size = 3, vjust = -0.5, hjust = 0.5, color = "grey") +
  scale_fill_manual(values = c(alpha("#0072B2", 1),
                               alpha("#E69F00", 1),
                               alpha("#D55E00", 1))) +
  geom_hline(yintercept = 0, linetype = "dashed", color = "#666666") +
```


Biology Open • Supplementary information

```
  labs(title = "(b) Pupae",
       x = "",
       y = "Relative body weight loss (%)") +
  theme_classic() +
  theme(
    plot.title = element_text(hjust = 0, vjust = 1),
    text = element_text(size = 10),
    legend.position = "none",
    plot.margin = margin(0, 1, 0, 0, "cm")
  )

# Fig2c ----
Fig2c <- ggplot(Df_Adults,aes(x=treatment, y=dry_mass*1000, fill = treatment)) +
  geom_boxplot(outlier.shape = NA)+
  geom_jitter(aes(fill = treatment), size = 2, alpha = 0.2, width = 0.15) +
  scale_fill_manual(values = c(alpha("#0072B2", 1),
                               alpha("#E69F00", 1),
                               alpha("#D55E00", 1))) +
  labs(title = "(c) Adults",
       x = "",
       y = "Dry mass (mg)") +
  theme_classic() +
  theme(
    plot.title = element_text(hjust = 0, vjust = 1),
    text = element_text(size = 10),
    legend.position = "none",
    plot.margin = margin(0, 1, 0, 0, "cm")
  )
```

```
Df_Adults <- Df1 %>%
  filter(developmental_stage_reached == "Adult")

m0d <- glmmTMB(
  log10(itd) ~ 1 + (1|colony) , # + (1|sex)
  family = Gamma(link = "log"), Df_Adults)
# FULL MODEL
m4 <- glmmTMB(
  log10(itd) ~ treatment + (1|colony) , #+ (1|sex)
  family = Gamma(link = "log"), Df_Adults)
summary(m4)
```

Analysis of adult body size using ITD (intertegular distance) as proxy

```
##  Family: Gamma  ( log )
## Formula:          log10(itd) ~ treatment + (1 | colony)
## Data: Df_Adults
##
##      AIC      BIC   logLik deviance df.resid
##   -277.0   -264.9    143.5   -287.0       78
##
```

```
## Random effects:
##
## Conditional model:
##  Groups Name        Variance Std.Dev.
##  colony (Intercept) 0.001184 0.03442
## Number of obs: 83, groups:  colony, 5
##
## Dispersion estimate for Gamma family (sigma^2): 0.00889
##
## Conditional model:
##                 Estimate Std. Error z value Pr(>|z|)
## (Intercept)   -0.806819   0.022924  -35.19   <2e-16 ***
## treatment37°C -0.008044   0.024173   -0.33    0.739
## treatment38°C -0.045521   0.028773   -1.58    0.114
## ---
## Signif. codes:  0 '***' 0.001 '**' 0.01 '*' 0.05 '.' 0.1 ' ' 1
```

```
Anova(m4)
```

```
## Analysis of Deviance Table (Type II Wald chisquare tests)
##
## Response: log10(itd)
##           Chisq Df Pr(>Chisq)
## treatment 2.584  2     0.2747
```

```
### Post-hoc tests
means4 <- emmeans(m4, ~ treatment) # Obtain marginal means
posthoc_4 <- pairs(means4, adjust="Tukey")
posthoc_4
```

```
##  contrast    estimate     SE  df z.ratio p.value
##  Ctrl - 37°C  0.00804 0.0242 Inf   0.333  0.9408
##  Ctrl - 38°C  0.04552 0.0288 Inf   1.582  0.2534
##  37°C - 38°C  0.03748 0.0298 Inf   1.258  0.4192
##
## Results are given on the log (not the response) scale.
## P value adjustment: tukey method for comparing a family of 3 estimates
```

```
# Lower AIC or BIC values indicate better-fitting models
AIC(m0d, m4)
```

```
##     df       AIC
## m0d  3 -278.4876
## m4   5 -277.0046
```

```
BIC(m0d, m4)
```

```
##     df       BIC
## m0d  3 -271.2311
## m4   5 -264.9104
```


Biology Open • Supplementary information

```
# Compared to NULL model
lrtest(m0d, m4)
```

```
## Likelihood ratio test
##
## Model 1: log10(itd) ~ 1 + (1 | colony)
## Model 2: log10(itd) ~ treatment + (1 | colony)
##   #Df LogLik Df Chisq Pr(>Chisq)
## 1   3 142.24
## 2   5 143.50  2 2.517     0.2841
```

```
anova(m0d, m4)
```

```
## Data: Df_Adults
## Models:
## m0d: log10(itd) ~ 1 + (1 | colony), zi=~0, disp=~1
## m4: log10(itd) ~ treatment + (1 | colony), zi=~0, disp=~1
##     Df     AIC     BIC logLik deviance Chisq Chi Df Pr(>Chisq)
## m0d  3 -278.49 -271.23 142.24  -284.49
## m4   5 -277.00 -264.91 143.50  -287.00 2.517      2     0.2841
```

```
# Model diagnostics
testDispersion(m4)
```

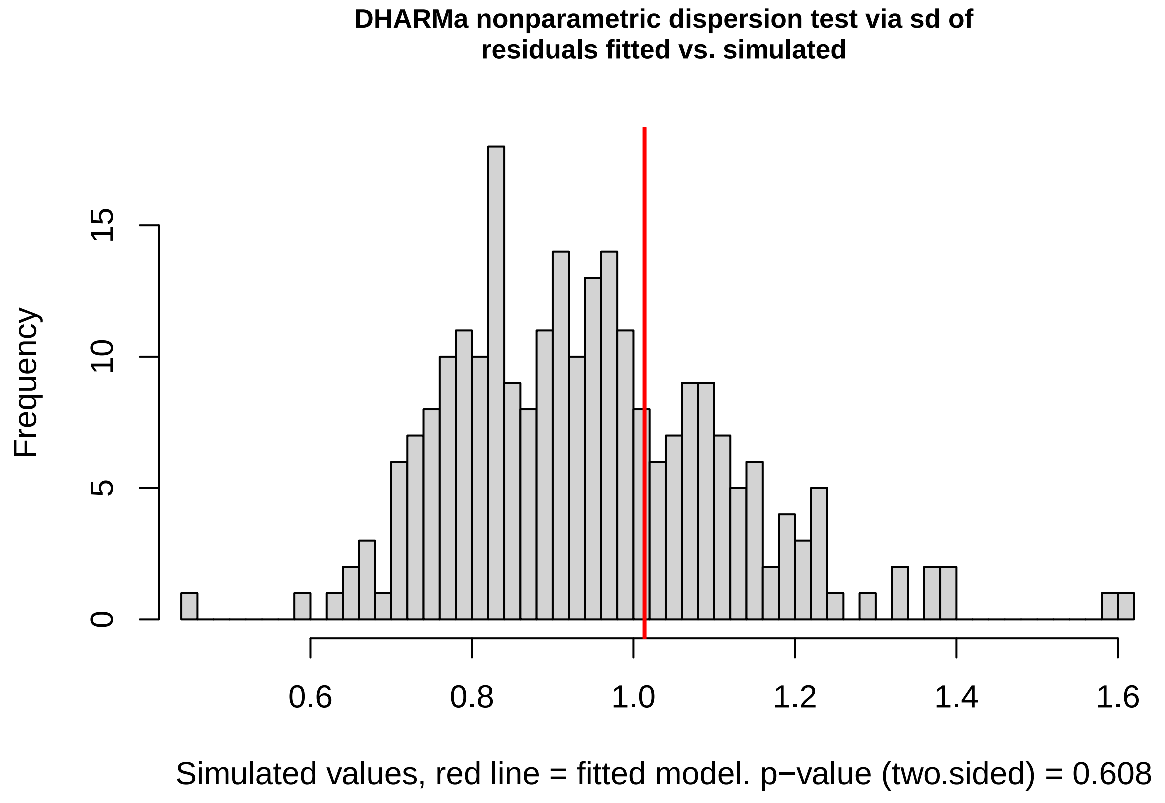

Biology Open • Supplementary information

```
##
##  DHARMa nonparametric dispersion test via sd of residuals fitted vs.
##  simulated
##
## data:  simulationOutput
## dispersion = 1.074, p-value = 0.608
## alternative hypothesis: two.sided
```

```
simulationOutput <- simulateResiduals(fittedModel = m4, plot = F)
residuals(simulationOutput)
```

```
##  [1] 0.076 0.624 0.556 0.152 0.468 0.816 0.808 0.960 0.716 0.332 0.768 0.996
## [13] 0.376 0.392 0.456 0.424 0.272 0.324 0.264 0.532 0.468 0.256 0.268 0.600
## [25] 0.692 0.396 0.612 0.560 0.496 0.500 0.584 0.972 0.288 0.336 0.564 0.344
## [37] 0.288 0.728 0.856 0.352 0.904 0.488 0.184 0.432 0.120 0.948 0.604 0.340
## [49] 0.564 0.956 0.340 0.920 0.380 0.192 0.636 0.104 0.352 0.440 0.964 0.216
## [61] 0.772 0.824 0.104 0.008 0.196 0.024 0.020 0.016 0.452 0.992 0.776 0.628
## [73] 0.356 0.660 0.336 0.084 0.336 0.632 0.272 0.976 0.884 0.976 0.972
```

```
residuals(simulationOutput, quantileFunction = qnorm, outlierValues = c(-7,7))
```

```
##  [1] -1.43250272  0.31600330  0.14083537 -1.02789335 -0.08029831  0.90022599
##  [7]  0.87054983  1.75068607  0.57099947 -0.43439724  0.73227620  2.65206981
## [13] -0.31600330 -0.27411012 -0.11051620 -0.19167090 -0.60677536 -0.45654238
## [19] -0.63106198  0.08029831 -0.08029831 -0.65572668 -0.61887304  0.25334710
## [25]  0.50152740 -0.26371440  0.28453554  0.15096922 -0.01002668  0.00000000
## [31]  0.21213720  1.91103565 -0.55923698 -0.42340472  0.16111859 -0.40157070
## [37] -0.55923698  0.60677536  1.06251930 -0.37992647  1.30468539 -0.03008408
## [43] -0.90022599 -0.17128459 -1.17498679  1.62576339  0.26371440 -0.41246313
## [49]  0.16111859  1.70604340 -0.41246313  1.40507156 -0.30548079 -0.87054983
## [55]  0.34778720 -1.25908398 -0.37992647 -0.15096922  1.79911811 -0.78577383
## [61]  0.74544955  0.93071695 -1.25908398 -2.40891555 -0.85599599 -1.97736843
## [67] -2.05374891 -2.14441062 -0.12060993  2.40891555  0.75875354  0.32656093
## [73] -0.36917136  0.41246313 -0.42340472 -1.37865873 -0.42340472  0.33715508
## [79] -0.60677536  1.97736843  1.19522278  1.97736843  1.91103565
```

```
plot(simulationOutput)
```


Biology Open • Supplementary information

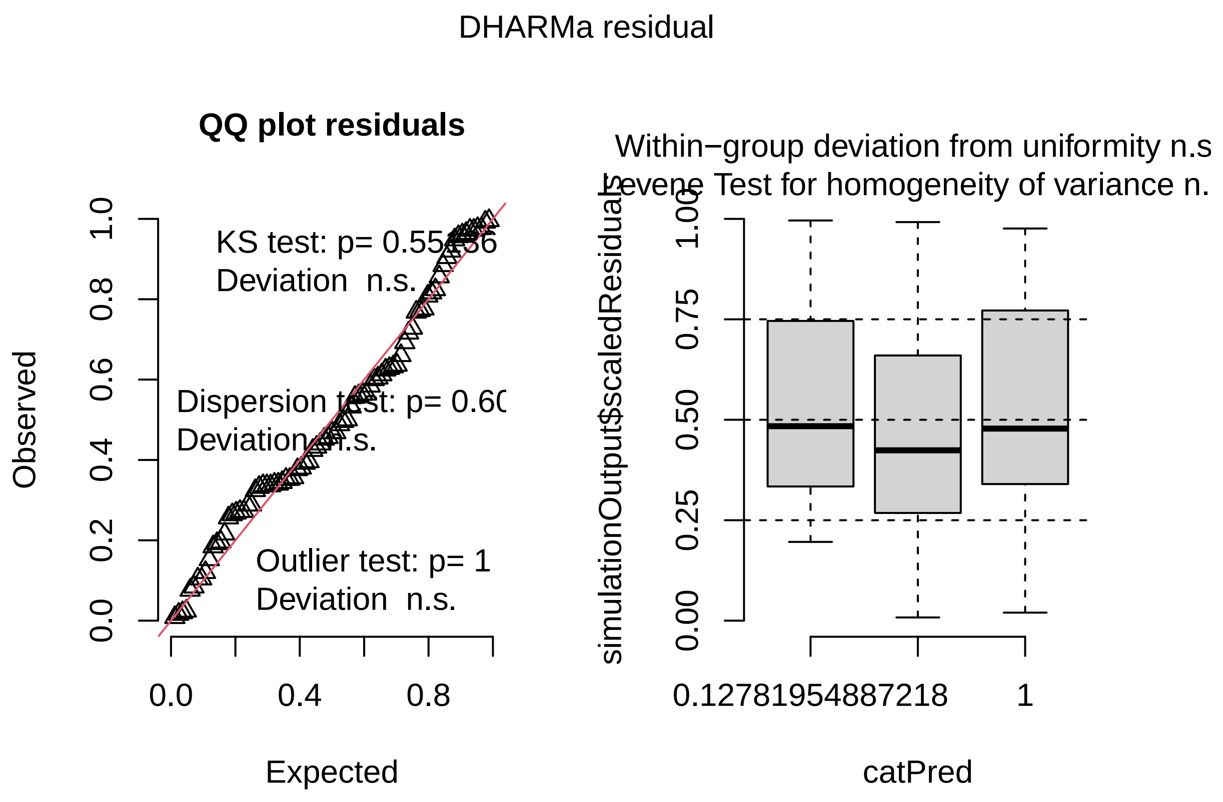


```
Df_Adults <- Df1 %>%
  filter(developmental_stage_reached == "Adult")

m0d3 <- glmmTMB(
  log10(headwidth) ~ 1 + (1|colony) + (1|sex),
  family = Gamma(link = "log"), Df_Adults)
# FULL MODEL
m4d <- glmmTMB(
  log10(headwidth) ~ treatment + (1|colony) + (1|sex),
  family = Gamma(link = "log"), Df_Adults)
summary(m4d)
```

Analysis of adult body size using head width as proxy

```
##  Family: Gamma  ( log )
## Formula:          log10(headwidth) ~ treatment + (1 | colony) + (1 | sex)
## Data: Df_Adults
##
##      AIC      BIC   logLik deviance df.resid
##   -342.8   -328.3    177.4   -354.8       77
##
## Random effects:
##
```

```
## Conditional model:
##  Groups Name        Variance  Std.Dev.
##  colony (Intercept) 8.234e-05 0.009074
##  sex    (Intercept) 1.575e-03 0.039685
## Number of obs: 83, groups:  colony, 5; sex, 2
##
## Dispersion estimate for Gamma family (sigma^2): 0.00294
##
## Conditional model:
##               Estimate Std. Error z value Pr(>|z|)
## (Intercept)   -0.65922    0.03026 -21.787   <2e-16 ***
## treatment37°C -0.00034    0.01367  -0.025    0.980
## treatment38°C -0.02300    0.01658  -1.387    0.165
## ---
## Signif. codes:  0 '***' 0.001 '**' 0.01 '*' 0.05 '.' 0.1 ' ' 1
```

```
Anova(m4d)
```

```
## Analysis of Deviance Table (Type II Wald chisquare tests)
##
## Response: log10(headwidth)
##            Chisq Df Pr(>Chisq)
## treatment 2.2459  2     0.3253
```

```
### Post-hoc tests
means4d <- emmeans(m4d, ~ treatment) # Obtain marginal means
posthoc_4d <- pairs(means4d, adjust="Tukey")
posthoc_4d
```

```
##  contrast    estimate     SE  df z.ratio p.value
##  Ctrl - 37°C  0.00034 0.0137 Inf   0.025  0.9997
##  Ctrl - 38°C  0.02300 0.0166 Inf   1.387  0.3475
##  37°C - 38°C  0.02266 0.0168 Inf   1.346  0.3697
##
## Results are given on the log (not the response) scale.
## P value adjustment: tukey method for comparing a family of 3 estimates
```

```
# Lower AIC or BIC values indicate better-fitting models
AIC(m0d3, m4d)
```

```
##      df       AIC
## m0d3  4 -344.5745
## m4d   6 -342.7764
```

```
BIC(m0d3, m4d)
```

```
##      df       BIC
## m0d3  4 -334.8992
## m4d   6 -328.2634
```


Biology Open • Supplementary information

```r
# Compared to NULL model
lrtest(m0d3, m4d)
```

```
## Likelihood ratio test
##
## Model 1: log10(headwidth) ~ 1 + (1 | colony) + (1 | sex)
## Model 2: log10(headwidth) ~ treatment + (1 | colony) + (1 | sex)
##   #Df LogLik Df  Chisq Pr(>Chisq)
## 1   4 176.29
## 2   6 177.39  2 2.2019     0.3326
```

```r
anova(m0d3, m4d)
```

```
## Data: Df_Adults
## Models:
## m0d3: log10(headwidth) ~ 1 + (1 | colony) + (1 | sex), zi=~0, disp=~1
## m4d: log10(headwidth) ~ treatment + (1 | colony) + (1 | sex), zi=~0, disp=~1
##      Df     AIC     BIC logLik deviance  Chisq Chi Df Pr(>Chisq)
## m0d3  4 -344.57 -334.90 176.29  -352.57
## m4d   6 -342.78 -328.26 177.39  -354.78 2.2019      2     0.3326
```

```r
# Model diagnostics
testDispersion(m4d)
```

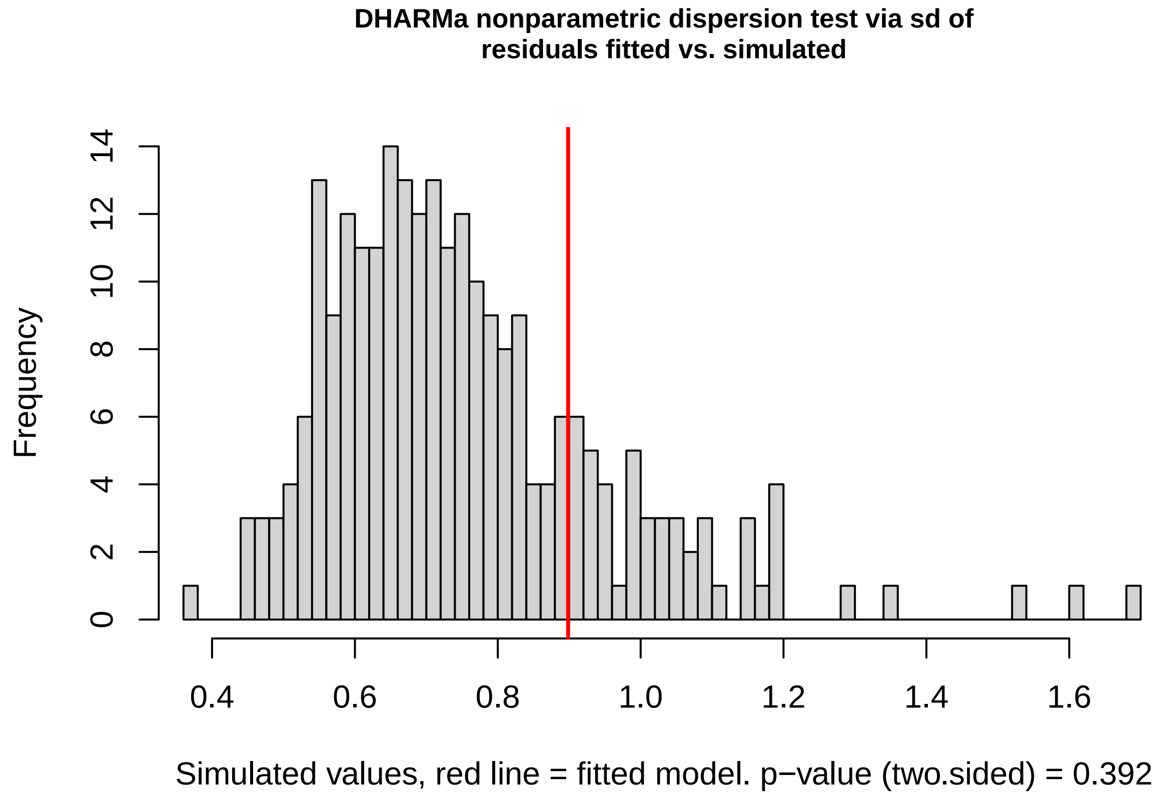

Biology Open • Supplementary information

```
##
##  DHARMa nonparametric dispersion test via sd of residuals fitted vs.
##  simulated
##
## data:  simulationOutput
## dispersion = 1.1906, p-value = 0.392
## alternative hypothesis: two.sided
```

```
simulationOutput <- simulateResiduals(fittedModel = m4d, plot = F)
residuals(simulationOutput)
```

```
##  [1] 0.124 0.756 0.668 0.104 0.524 0.732 0.612 0.876 0.644 0.268 0.704 0.980
## [13] 0.316 0.428 0.252 0.276 0.176 0.328 0.360 0.496 0.320 0.088 0.220 0.636
## [25] 0.600 0.588 0.544 0.476 0.788 0.424 0.756 0.860 0.308 0.228 0.192 0.104
## [37] 0.060 0.468 0.868 0.492 0.740 0.336 0.092 0.564 0.148 0.768 0.176 0.416
## [49] 0.404 0.800 0.328 0.444 0.164 0.160 0.356 0.028 0.232 0.304 0.720 0.140
## [61] 0.884 0.504 0.056 0.016 0.084 0.004 0.012 0.068 0.396 0.964 0.584 0.628
## [73] 0.368 0.536 0.164 0.060 0.248 0.312 0.020 0.932 0.632 0.968 0.800
```

```
residuals(simulationOutput, quantileFunction = qnorm, outlierValues = c(-7,7))
```

```
##  [1] -1.15522085  0.69349335  0.43439724 -1.25908398  0.06019541  0.61887304
##  [7]  0.28453554  1.15522085  0.36917136 -0.61887304  0.53594003  2.05374891
## [13] -0.47891373 -0.18146832 -0.66820930 -0.59476585 -0.93071695 -0.44544251
## [19] -0.35845879 -0.01002668 -0.46769880 -1.35317415 -0.77219321  0.34778720
## [25]  0.25334710  0.22240323  0.11051620 -0.06019541  0.79950094 -0.19167090
## [31]  0.69349335  1.08031934 -0.50152740 -0.74544955 -0.87054983 -1.25908398
## [37] -1.55477359 -0.08029831  1.11698673 -0.02005437  0.64334541 -0.42340472
## [43] -1.32853933  0.16111859 -1.04504970  0.73227620 -0.93071695 -0.21213720
## [49] -0.24300697  0.84162123 -0.44544251 -0.14083537 -0.97815029 -0.99445788
## [55] -0.36917136 -1.91103565 -0.73227620 -0.51293041  0.58284151 -1.08031934
## [61]  1.19522278  0.01002668 -1.58926756 -2.14441062 -1.37865873 -2.65206981
## [67] -2.25712924 -1.49085336 -0.26371440  1.79911811  0.21213720  0.32656093
## [73] -0.33715508  0.09036144 -0.97815029 -1.55477359 -0.68079692 -0.49018923
## [79] -2.05374891  1.49085336  0.33715508  1.85217986  0.84162123
```

```
plot(simulationOutput)
```


Biology Open • Supplementary information

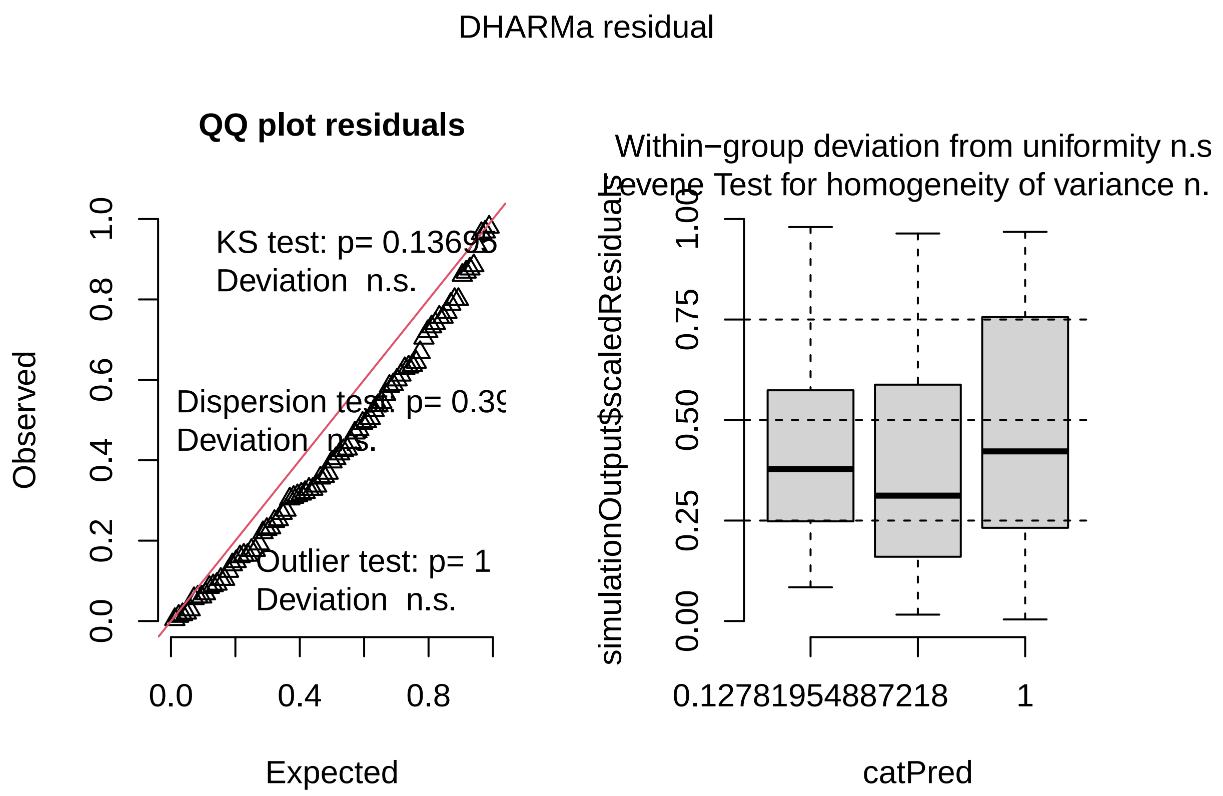


```
Df_Adults <- Df_Adults %>%
  mutate(rel_fat = fat/dry_mass)

m0d4 <- glmmTMB(
  rel_fat ~ 1 + (1|colony) + (1|sex),
  family = "gaussian", Df_Adults)
```

Analysis of relative lipid content

```
## Warning in finalizeTMB(TMBStruc, obj, fit, h, data.tmb.old): failed to invert
## Hessian from numDeriv::jacobian(), falling back to internal vcov estimate
```

```
# FULL MODEL
m4d <- glmmTMB(
  rel_fat ~ treatment + (1|colony) + (1|sex),
  family = "gaussian", Df_Adults)
```

```
## Warning in finalizeTMB(TMBStruc, obj, fit, h, data.tmb.old): Model convergence
## problem; non-positive-definite Hessian matrix. See vignette('troubleshooting')
```


Biology Open • Supplementary information

```r
summary(m4d)
```

```
##  Family: gaussian  ( identity )
## Formula:          rel_fat ~ treatment + (1 | colony) + (1 | sex)
## Data: Df_Adults
##
##      AIC      BIC   logLik deviance df.resid
##       NA       NA       NA       NA       77
##
## Random effects:
##
## Conditional model:
##  Groups   Name        Variance  Std.Dev.
##  colony   (Intercept) 5.506e-11 7.420e-06
##  sex      (Intercept) 6.401e-12 2.530e-06
##  Residual             3.142e-03 5.605e-02
## Number of obs: 83, groups:  colony, 5; sex, 2
##
## Dispersion estimate for gaussian family (sigma^2): 0.00314
##
## Conditional model:
##                Estimate Std. Error z value Pr(>|z|)
## (Intercept)    0.122504   0.009613  12.743   <2e-16 ***
## treatment37°C 0.026336   0.013698   1.923   0.0545 .
## treatment38°C 0.022537   0.016994   1.326   0.1848
## ---
## Signif. codes:  0 '***' 0.001 '**' 0.01 '*' 0.05 '.' 0.1 ' ' 1
```

```r
Anova(m4d)
```

```
## Analysis of Deviance Table (Type II Wald chisquare tests)
##
## Response: rel_fat
##            Chisq Df Pr(>Chisq)
## treatment 4.0727  2     0.1305
```

```r
### Post-hoc tests
means4d <- emmeans(m4d, ~ treatment) # Obtain marginal means
posthoc_4d <- pairs(means4d, adjust="Tukey")
posthoc_4d
```

```
##  contrast    estimate     SE df t.ratio p.value
##  Ctrl - 37°C  -0.0263 0.0137 77  -1.923  0.1392
##  Ctrl - 38°C  -0.0225 0.0170 77  -1.326  0.3851
##  37°C - 38°C   0.0038 0.0171 77   0.222  0.9731
##
## P value adjustment: tukey method for comparing a family of 3 estimates
```

```r
# Lower AIC or BIC values indicate better-fitting models
AIC(m0d4, m4d)
```


Biology Open • Supplementary information

```
##      df       AIC
## m0d4  4 -230.7964
## m4d   6        NA
```

```
BIC(m0d4, m4d)
```

```
##      df       BIC
## m0d4  4 -221.1211
## m4d   6        NA
```

```
# Compared to NULL model
lrtest(m0d4, m4d)
```

```
## Likelihood ratio test
##
## Model 1: rel_fat ~ 1 + (1 | colony) + (1 | sex)
## Model 2: rel_fat ~ treatment + (1 | colony) + (1 | sex)
##   #Df LogLik Df Chisq Pr(>Chisq)
## 1   4  119.4
## 2   6         2
```

```
anova(m0d4, m4d)
```

```
## Data: Df_Adults
## Models:
## m0d4: rel_fat ~ 1 + (1 | colony) + (1 | sex), zi=~0, disp=~1
## m4d: rel_fat ~ treatment + (1 | colony) + (1 | sex), zi=~0, disp=~1
##      Df    AIC     BIC logLik deviance Chisq Chi Df Pr(>Chisq)
## m0d4  4 -230.8 -221.12  119.4   -238.8
## m4d   6                                           2
```

```
# Model diagnostics
testDispersion(m4d)
```

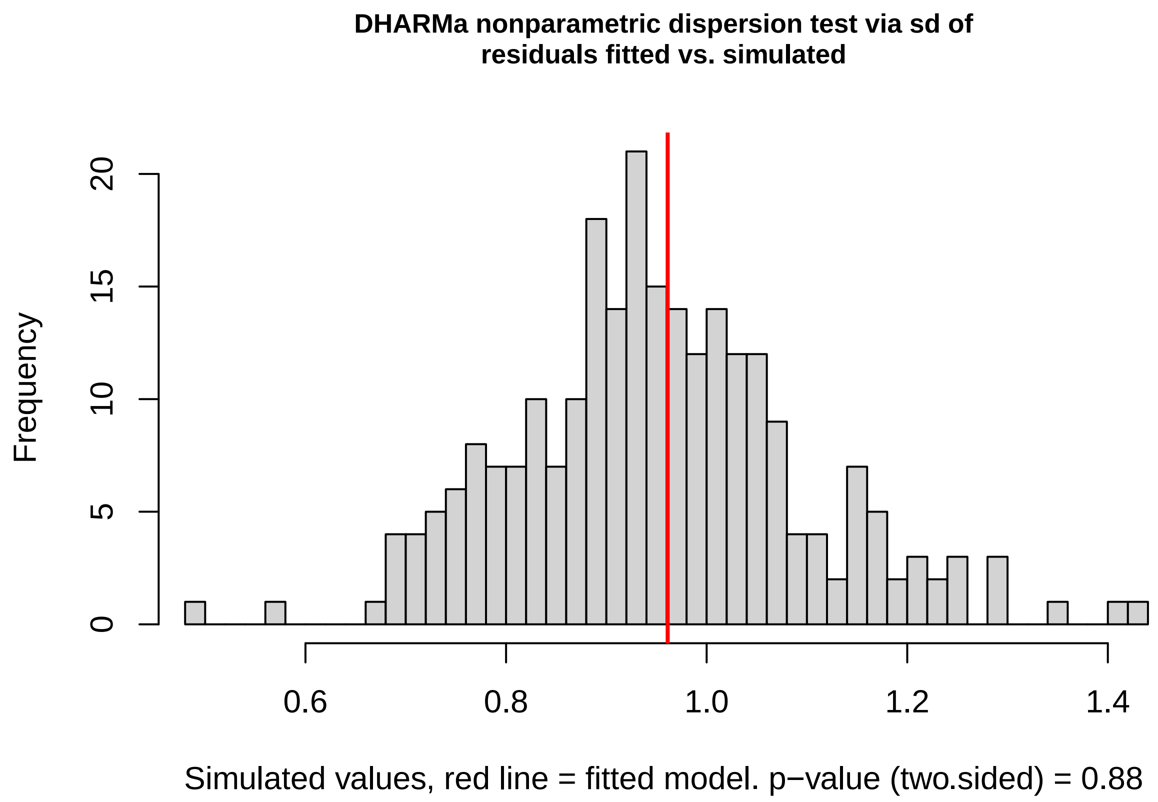


```
##
##  DHARMa nonparametric dispersion test via sd of residuals fitted vs.
##  simulated
##
## data:  simulationOutput
## dispersion = 1.0102, p-value = 0.88
## alternative hypothesis: two.sided
```

```
simulationOutput <- simulateResiduals(fittedModel = m4d, plot = F)
residuals(simulationOutput)
```

```
##  [1] 0.924 0.516 0.344 0.732 0.028 0.548 0.240 0.264 0.168 0.608 0.752 0.676
## [13] 0.856 0.256 0.664 0.076 0.144 0.644 0.244 0.412 0.484 0.196 0.164 0.904
## [25] 0.184 0.088 0.576 0.456 0.412 0.044 0.708 0.692 0.784 0.528 0.564 0.288
## [37] 0.148 0.448 0.748 0.476 0.952 0.520 0.300 0.964 0.824 0.676 0.352 0.020
## [49] 0.020 0.864 0.944 0.476 0.236 0.616 0.812 0.544 0.588 0.956 0.996 0.308
## [61] 0.972 0.608 0.804 0.004 0.228 0.200 0.032 0.064 0.912 0.708 0.892 0.344
## [73] 0.528 0.852 0.620 0.432 0.452 0.680 0.632 0.644 0.332 0.744 0.612
```

```
residuals(simulationOutput, quantileFunction = qnorm, outlierValues = c(-7,7))
```

```
##  [1]  1.43250272  0.04011681 -0.40157070  0.61887304 -1.91103565  0.12060993
##  [7] -0.70630256 -0.63106198 -0.96209875  0.27411012  0.68079692  0.45654238
## [13]  1.06251930 -0.65572668  0.42340472 -1.43250272 -1.06251930  0.36917136
```


Biology Open • Supplementary information

```
## [19] -0.69349335 -0.22240323 -0.04011681 -0.85599599 -0.97815029  1.30468539
## [25] -0.90022599 -1.35317415  0.19167090 -0.11051620 -0.22240323 -1.70604340
## [31]  0.54755135  0.50152740  0.78577383  0.07024331  0.16111859 -0.55923698
## [37] -1.04504970 -0.13071597  0.66820930 -0.06019541  1.66456286  0.05015358
## [43] -0.52440051  1.79911811  0.93071695  0.45654238 -0.37992647 -2.05374891
## [49] -2.05374891  1.09846842  1.58926756 -0.06019541 -0.71922873  0.29499199
## [55]  0.88529045  0.11051620  0.22240323  1.70604340  2.65206981 -0.50152740
## [61]  1.91103565  0.27411012  0.85599599 -2.65206981 -0.74544955 -0.84162123
## [67] -1.85217986 -1.52203624  1.35317415  0.54755135  1.23723460 -0.40157070
## [73]  0.07024331  1.04504970  0.30548079 -0.17128459 -0.12060993  0.46769880
## [79]  0.33715508  0.36917136 -0.43439724  0.65572668  0.28453554
```

```
plot(simulationOutput)
```

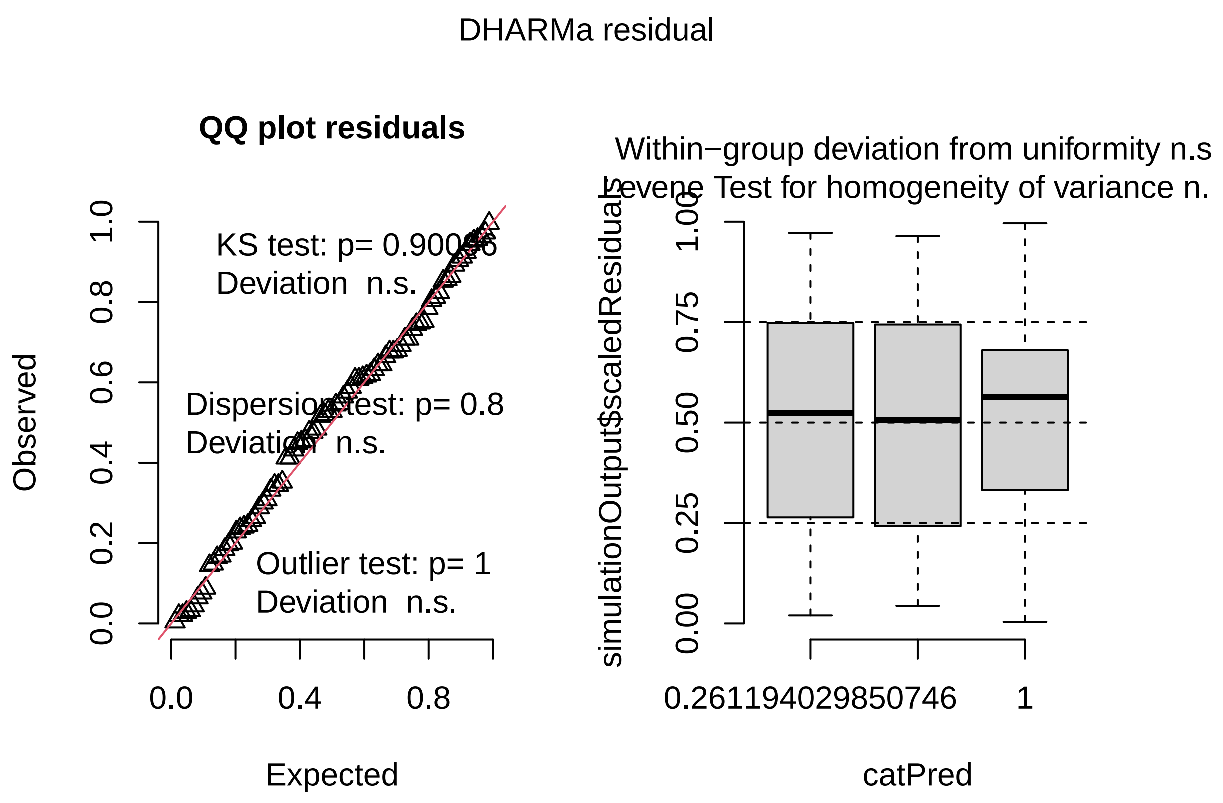

Figure 2 bottom

```
# Fig2d ----
Fig2d <- ggplot(Df_Adults,aes(x=treatment, y=itd, fill = treatment)) +
  geom_boxplot(outlier.shape = NA)+
  geom_jitter(aes(fill = treatment), size = 2, alpha = 0.2, width = 0.15) +
  scale_fill_manual(values = c(alpha("#0072B2", 1),
                               alpha("#E69F00", 1),
                               alpha("#D55E00", 1))) +
```


Biology Open • Supplementary information

```
  labs(title = "(d) Adults",
       x = "Treatment",
       y = "ITD (mm)") +
  theme_classic() +
  theme(
    plot.title = element_text(hjust = 0, vjust = 1),
    text = element_text(size = 10),
    legend.position = "none",
    plot.margin = margin(0, 1, 0, 0, "cm")
  )

# Fig2e ----
Fig2e <- ggplot(Df_Adults,aes(x=treatment, y=headwidth, fill = treatment)) +
  geom_boxplot(outlier.shape = NA)+
  geom_jitter(aes(fill = treatment), size = 2, alpha = 0.2, width = 0.15) +
  scale_fill_manual(values = c(alpha("#0072B2", 1),
                               alpha("#E69F00", 1),
                               alpha("#D55E00", 1))) +
  labs(title = "(e) Adults",
       x = "Treatment",
       y = "Head width (mm)") +
  theme_classic() +
  theme(
    plot.title = element_text(hjust = 0, vjust = 1),
    text = element_text(size = 10),
    legend.position = "none",
    plot.margin = margin(0, 1, 0, 0, "cm")
  )

# Fig2f ----

Fig2f <- ggplot(Df_Adults,aes(x=treatment, y=rel_fat*100, fill = treatment)) +
  geom_boxplot(outlier.shape = NA)+
  geom_jitter(aes(fill = treatment), size = 2, alpha = 0.2, width = 0.15) +
  scale_fill_manual(values = c(alpha("#0072B2", 1),
                               alpha("#E69F00", 1),
                               alpha("#D55E00", 1))) +
  labs(title = "(f) Adults",
       x = "Treatment",
       y = "Relative lipid content (%)") +
  theme_classic() +
  theme(
    plot.title = element_text(hjust = 0, vjust = 1),
    text = element_text(size = 10),
    legend.position = "none",
    plot.margin = margin(0, 1, 0, 0, "cm")
  )
```

Arrangement of Figure 2

```
Fig2 <- grid.arrange(
  arrangeGrob(Fig2a),
```

```
  arrangeGrob(Fig2b),
  arrangeGrob(Fig2c),
  arrangeGrob(Fig2d),
  arrangeGrob(Fig2e),
  arrangeGrob(Fig2f),
  layout_matrix = matrix(c(1, 2, 3,
                           4, 5, 6),
                         nrow = 2, byrow = TRUE)
)
```

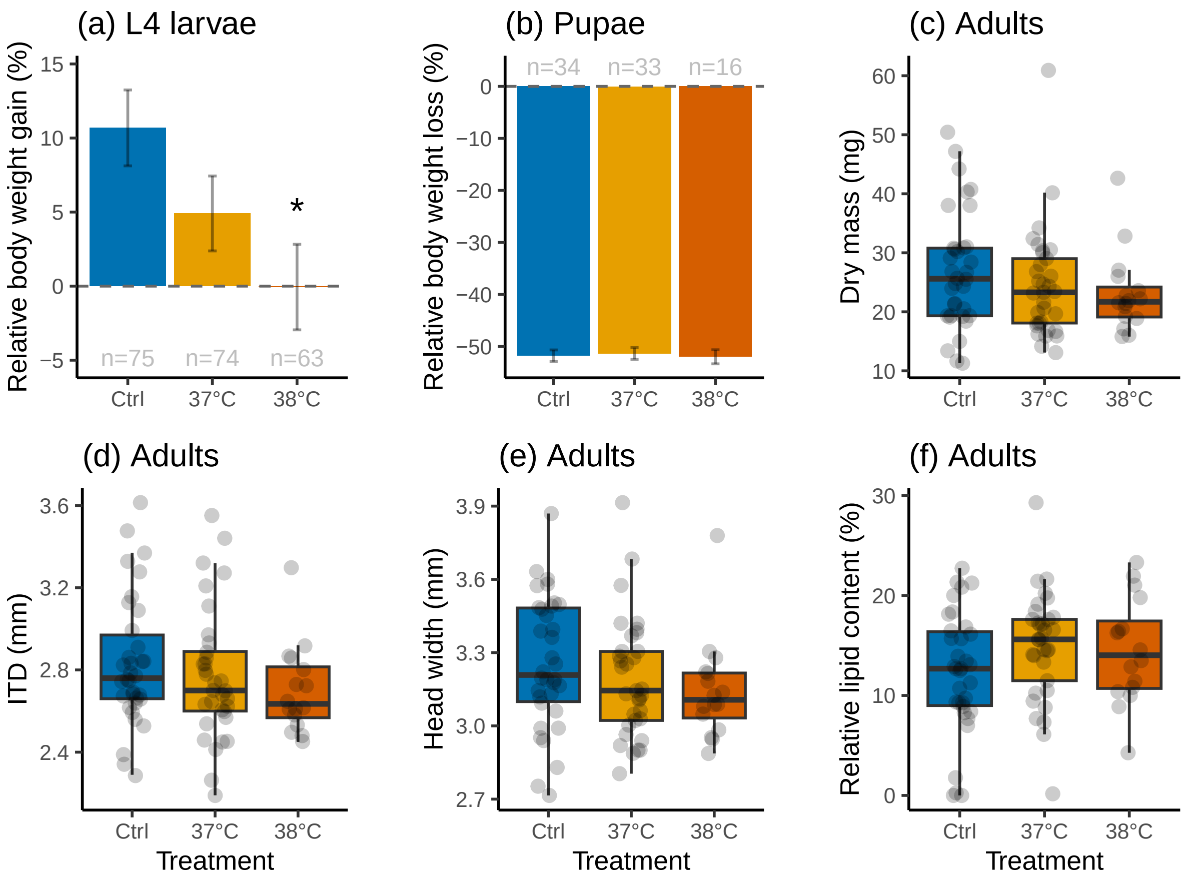


```
ggsave(Fig2, filename = "Fig2.png", width = 6, height = 6, dpi = 300, units = "in", device='png')
```

Analysis of effects of body weight gain during treatment on adult survival

```
Df <- Df[!is.na(Df$rel_BWG_treatment), ]

# NULL MODEL
m0g <- glmmTMB(
  reached_adulthood ~ 1 + (1|colony),
  family = "binomial", Df)
# FULL MODEL
m7 <- glmmTMB(
  reached_adulthood ~ treatment + rel_BWG_treatment + (1|colony),
```


Biology Open • Supplementary information

```r
  family = "binomial", Df)
summary(m7)
```

```
##  Family: binomial  ( logit )
## Formula:
## reached_adulthood ~ treatment + rel_BWG_treatment + (1 | colony)
## Data: Df
##
##      AIC      BIC   logLik deviance df.resid
##    251.9    268.7   -120.9    241.9      207
##
## Random effects:
##
## Conditional model:
##  Groups Name        Variance Std.Dev.
##  colony (Intercept) 0.4542   0.674
## Number of obs: 212, groups:  colony, 5
##
## Conditional model:
##                    Estimate Std. Error z value Pr(>|z|)
## (Intercept)        0.124435   0.410137   0.303 0.761585
## treatment37°C     -0.249947   0.376751  -0.663 0.507057
## treatment38°C     -1.593563   0.436826  -3.648 0.000264 ***
## rel_BWG_treatment -0.045249   0.009176  -4.931 8.17e-07 ***
## ---
## Signif. codes:  0 '***' 0.001 '**' 0.01 '*' 0.05 '.' 0.1 ' ' 1
```

```r
Anova(m7)
```

```
## Analysis of Deviance Table (Type II Wald chisquare tests)
##
## Response: reached_adulthood
##                    Chisq Df Pr(>Chisq)
## treatment         14.629  2  0.0006659 ***
## rel_BWG_treatment 24.318  1  8.168e-07 ***
## ---
## Signif. codes:  0 '***' 0.001 '**' 0.01 '*' 0.05 '.' 0.1 ' ' 1
```

```r
m8 <- glmmTMB(
  reached_adulthood ~ treatment * rel_BWG_treatment + (1|colony),
  family = "binomial", Df)
summary(m8)
```

```
##  Family: binomial  ( logit )
## Formula:
## reached_adulthood ~ treatment * rel_BWG_treatment + (1 | colony)
## Data: Df
##
##      AIC      BIC   logLik deviance df.resid
##    255.9    279.4   -120.9    241.9      205
##
## Random effects:
```


Biology Open • Supplementary information

```
##
## Conditional model:
##  Groups Name        Variance Std.Dev.
##  colony (Intercept) 0.461    0.679
## Number of obs: 212, groups:  colony, 5
##
## Conditional model:
##                                  Estimate Std. Error z value Pr(>|z|)
## (Intercept)                       0.123629   0.421060   0.294 0.769053
## treatment37°C                    -0.245550   0.391446  -0.627 0.530469
## treatment38°C                    -1.575125   0.445231  -3.538 0.000404 ***
## rel_BWG_treatment                -0.045259   0.014098  -3.210 0.001326 **
## treatment37°C:rel_BWG_treatment -0.001562   0.019195  -0.081 0.935161
## treatment38°C:rel_BWG_treatment  0.003088   0.023537   0.131 0.895631
## ---
## Signif. codes:  0 '***' 0.001 '**' 0.01 '*' 0.05 '.' 0.1 ' ' 1
```

```
Anova(m8)
```

```
## Analysis of Deviance Table (Type II Wald chisquare tests)
##
## Response: reached_adulthood
##                             Chisq Df Pr(>Chisq)
## treatment                 14.7587  2   0.000624 ***
## rel_BWG_treatment         24.3525  1  8.022e-07 ***
## treatment:rel_BWG_treatment  0.0401  2   0.980155
## ---
## Signif. codes:  0 '***' 0.001 '**' 0.01 '*' 0.05 '.' 0.1 ' ' 1
```

```
### Post-hoc tests
### Comparing slopes
em_means7 <- emmeans(m7, ~ treatment | rel_BWG_treatment) # Obtain marginal means
em_means7
```

```
## rel_BWG_treatment = 5.47:
##  treatment emmean    SE  df asymp.LCL asymp.UCL
##  Ctrl      -0.123 0.405 Inf    -0.918     0.672
##  37°C      -0.373 0.403 Inf    -1.164     0.418
##  38°C      -1.717 0.466 Inf    -2.631    -0.803
##
## Results are given on the logit (not the response) scale.
## Confidence level used: 0.95
```

```
em_means8 <- emmeans(m8, ~ treatment | rel_BWG_treatment) # Obtain marginal means
em_means8
```

```
## rel_BWG_treatment = 5.47:
##  treatment emmean    SE  df asymp.LCL asymp.UCL
##  Ctrl      -0.124 0.408 Inf    -0.924     0.676
##  37°C      -0.378 0.407 Inf    -1.177     0.421
##  38°C      -1.682 0.504 Inf    -2.669    -0.695
##
## Results are given on the logit (not the response) scale.
## Confidence level used: 0.95
```


Biology Open • Supplementary information

```r
# Lower AIC or BIC values indicate better-fitting models
AIC(m0g, m7, m8)
```

```
##     df      AIC
## m0g  2 285.9248
## m7   5 251.8973
## m8   7 255.8576
```

```r
BIC(m0g, m7, m8)
```

```
##     df      BIC
## m0g  2 292.6380
## m7   5 268.6802
## m8   7 279.3537
```

```r
lrtest(m7, m8)
```

```
## Likelihood ratio test
##
## Model 1: reached_adulthood ~ treatment + rel_BWG_treatment + (1 | colony)
## Model 2: reached_adulthood ~ treatment * rel_BWG_treatment + (1 | colony)
##   #Df  LogLik Df  Chisq Pr(>Chisq)
## 1   5 -120.95
## 2   7 -120.93  2 0.0397     0.9803
```

```r
# Compared to NULL model
lrtest(m0g, m7)
```

```
## Likelihood ratio test
##
## Model 1: reached_adulthood ~ 1 + (1 | colony)
## Model 2: reached_adulthood ~ treatment + rel_BWG_treatment + (1 | colony)
##   #Df  LogLik Df  Chisq Pr(>Chisq)
## 1   2 -140.96
## 2   5 -120.95  3 40.028  1.051e-08 ***
## ---
## Signif. codes:  0 '***' 0.001 '**' 0.01 '*' 0.05 '.' 0.1 ' ' 1
```

```r
anova(m0g, m7)
```

```
## Data: Df
## Models:
## m0g: reached_adulthood ~ 1 + (1 | colony), zi=~0, disp=~1
## m7: reached_adulthood ~ treatment + rel_BWG_treatment + (1 | colony), zi=~0, disp=~1
##     Df    AIC    BIC  logLik deviance  Chisq Chi Df Pr(>Chisq)
## m0g  2 285.93 292.64 -140.96   281.93
## m7   5 251.90 268.68 -120.95   241.90 40.028      3  1.051e-08 ***
## ---
## Signif. codes:  0 '***' 0.001 '**' 0.01 '*' 0.05 '.' 0.1 ' ' 1
```


Biology Open • Supplementary information

```
lrtest(m0g, m8)
```

```
## Likelihood ratio test
##
## Model 1: reached_adulthood ~ 1 + (1 | colony)
## Model 2: reached_adulthood ~ treatment * rel_BWG_treatment + (1 | colony)
##   #Df  LogLik Df  Chisq Pr(>Chisq)
## 1   2 -140.96
## 2   7 -120.93  5 40.067  1.447e-07 ***
## ---
## Signif. codes:  0 '***' 0.001 '**' 0.01 '*' 0.05 '.' 0.1 ' ' 1
```

```
anova(m0g, m8)
```

```
## Data: Df
## Models:
## m0g: reached_adulthood ~ 1 + (1 | colony), zi=~0, disp=~1
## m8: reached_adulthood ~ treatment * rel_BWG_treatment + (1 | colony), zi=~0, disp=~1
##     Df    AIC    BIC  logLik deviance  Chisq Chi Df Pr(>Chisq)
## m0g  2 285.93 292.64 -140.96   281.93
## m8   7 255.86 279.35 -120.93   241.86 40.067      5  1.447e-07 ***
## ---
## Signif. codes:  0 '***' 0.001 '**' 0.01 '*' 0.05 '.' 0.1 ' ' 1
```

```
# Model diagnostics
testDispersion(m7)
```


Biology Open • Supplementary information

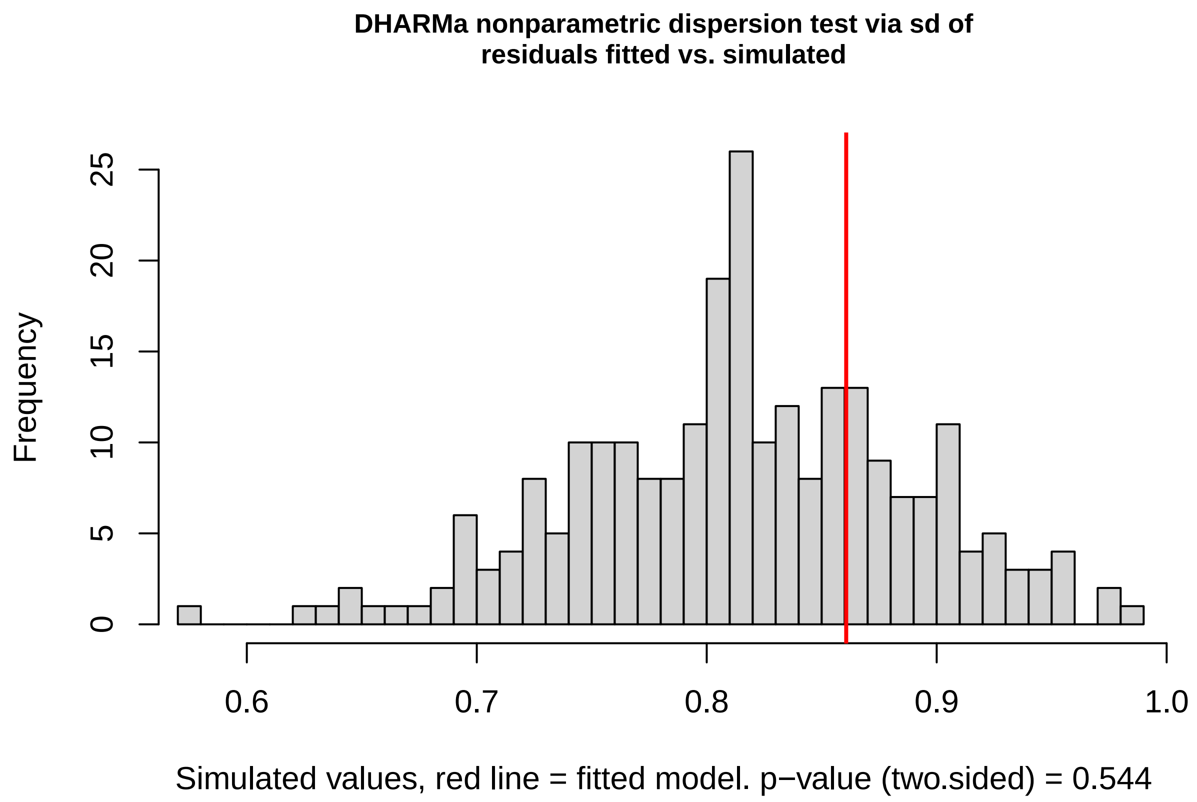


```
##
##  DHARMa nonparametric dispersion test via sd of residuals fitted vs.
##  simulated
##
## data:  simulationOutput
## dispersion = 1.0559, p-value = 0.544
## alternative hypothesis: two.sided
```

```
simulationOutput <- simulateResiduals(fittedModel = m7, plot = F)
residuals(simulationOutput)
```

```
##   [1] 0.324299843 0.309455162 0.929199181 0.229358871 0.029733374 0.444524181
##   [7] 0.185557748 0.764361944 0.259899367 0.822206700 0.131801435 0.497466706
##  [13] 0.518249592 0.582094105 0.486291943 0.239976415 0.062767557 0.222008156
##  [19] 0.222821161 0.350216961 0.050818096 0.379093835 0.735138171 0.424855951
##  [25] 0.585254410 0.201469731 0.669287126 0.922914992 0.837254973 0.869404967
##  [31] 0.999174838 0.877904705 0.776079465 0.411764957 0.245897881 0.809230190
##  [37] 0.091066864 0.328933330 0.453130837 0.188168981 0.338320782 0.834031648
##  [43] 0.407819584 0.296536104 0.581760058 0.561730992 0.921144479 0.342664741
##  [49] 0.282776747 0.226529370 0.429029149 0.611674922 0.702075875 0.401170650
##  [55] 0.897131719 0.041627238 0.724898481 0.953696141 0.974609756 0.647474226
##  [61] 0.962228158 0.471263615 0.405123675 0.590009385 0.912043243 0.742274414
##  [67] 0.178343853 0.963241267 0.357199105 0.567892355 0.375557361 0.786216931
##  [73] 0.163516465 0.872279120 0.107649990 0.458210493 0.489955144 0.515410011
##  [79] 0.877859582 0.764739541 0.415102031 0.369115769 0.001989429 0.983845923
```


Biology Open • Supplementary information

```
##  [85] 0.647573101 0.875090412 0.984637864 0.407214774 0.853816693 0.288241631
##  [91] 0.721340935 0.698076696 0.785032397 0.727647755 0.861225596 0.992428948
##  [97] 0.498571550 0.723597494 0.015323441 0.679090558 0.741930488 0.442649989
## [103] 0.457211560 0.839903188 0.938251491 0.394480103 0.543382458 0.805058859
## [109] 0.427627759 0.836615841 0.990963963 0.809757933 0.935454837 0.067233279
## [115] 0.562502847 0.919111348 0.554910020 0.355288024 0.755107662 0.246230661
## [121] 0.251053271 0.998656932 0.537221687 0.041120597 0.991406319 0.101953867
## [127] 0.761816029 0.800594296 0.050291303 0.415545318 0.588018968 0.844951672
## [133] 0.647937173 0.289006926 0.430502213 0.102057225 0.353461279 0.716627166
## [139] 0.845217976 0.982417017 0.206450602 0.407430554 0.666832660 0.292443056
## [145] 0.215921307 0.855154196 0.349641891 0.404072501 0.915111720 0.455948805
## [151] 0.828230664 0.787570029 0.868277782 0.406521260 0.993226728 0.804298685
## [157] 0.102563486 0.371529152 0.022506128 0.534202014 0.714788627 0.047665046
## [163] 0.597094937 0.431367025 0.008193744 0.003776040 0.547324902 0.108774580
## [169] 0.209452691 0.028841959 0.275177965 0.669515622 0.348022332 0.918733084
## [175] 0.826785019 0.706451980 0.883587367 0.977626357 0.177443630 0.591290471
## [181] 0.337444508 0.211304234 0.307338609 0.468959423 0.483021718 0.155344682
## [187] 0.185615745 0.212390845 0.419096405 0.246152590 0.079803446 0.097262579
## [193] 0.124597327 0.909002266 0.518212785 0.168128548 0.763037288 0.115490358
## [199] 0.246311622 0.991595097 0.248361131 0.113570524 0.126482469 0.250299862
## [205] 0.783066352 0.583437116 0.656086974 0.769030840 0.475743409 0.903872077
## [211] 0.688060244 0.381406453
```

```
residuals(simulationOutput, quantileFunction = qnorm, outlierValues = c(-7,7))
```

```
##   [1] -0.455708392 -0.497395293  1.469852756 -0.740959919 -1.884726688
##   [6] -0.139508471 -0.894385823  0.720404287 -0.643655683  0.923807402
##  [11] -1.117916002 -0.006350069  0.045760909  0.207253624 -0.034367768
##  [16] -0.706378432 -1.531948624 -0.765428696 -0.762700013 -0.384734783
##  [21] -1.636972613 -0.307861568  0.628427912 -0.189486031  0.215354193
##  [26] -0.836383018  0.437945560  1.424955670  0.983238534  1.123582775
##  [31]  3.146862930  1.164576180  0.759019203 -0.223007185 -0.687455456
##  [36]  0.875063014 -1.334214017 -0.442860473 -0.117755142 -0.884663844
##  [41] -0.417050371  0.970220282 -0.233157421 -0.534389319  0.206398191
##  [46]  0.155359368  1.412811888 -0.405201392 -0.574612354 -0.750325414
##  [51] -0.178846417  0.283687134  0.530380347 -0.250318177  1.265376033
##  [56] -1.732107208  0.597455905  1.681799543  1.953330145  0.378510293
##  [61]  1.777149265 -0.072093838 -0.240106946  0.227569120  1.353444987
##  [66]  0.650373218 -0.921694970  1.789604807 -0.365955597  0.171010778
##  [71] -0.317169857  0.793363382 -0.980107760  1.137230930 -1.239122919
##  [76] -0.104943066 -0.025181381  0.038636781  1.164353372  0.721631742
##  [81] -0.214439859 -0.334196166 -2.879833015  2.140577146  0.378776555
##  [86]  1.150788699  2.160625612 -0.234715512  1.052944099 -0.558528920
##  [91]  0.586829638  0.518876871  0.789302533  0.605714294  1.085842211
##  [96]  2.428962387 -0.003580601  0.593562138 -2.161627724  0.465157203
## [101]  0.649308453 -0.144253903 -0.107461180  0.994060070  1.540259881
## [106] -0.267661085  0.108958907  0.859830867 -0.182416960  0.980644020
## [111]  2.364138122  0.877004610  1.517698881 -1.496718355  0.157317909
## [116]  1.399118992  0.138076498 -0.371082548  0.690651340 -0.686399339
## [121] -0.671178943  3.001544706  0.093436712 -1.737827615  2.382674965
## [126] -1.270496769  0.712156383  0.843745909 -1.642035704 -0.213302998
## [131]  0.222451964  1.015019239  0.379757203 -0.556288200 -0.175095714
## [136] -1.269916288 -0.375992385  0.572850876  1.016137219  2.106442113
## [141] -0.818798820 -0.234159560  0.431183871 -0.546261622 -0.786042459
```


Biology Open • Supplementary information

```
## [146]  1.058798390 -0.386287466 -0.242819793  1.372922118 -0.110645316
## [151]  0.947196472  0.798018177  1.118287002 -0.236502820  2.469068086
## [156]  0.857076459 -1.267079198 -0.327806062 -2.004539921  0.085837026
## [161]  0.567429007 -1.667927635  0.245834788 -0.172894887 -2.400169367
## [166] -2.671465692  0.118905536 -1.233071312 -0.808321755 -1.898092342
## [171] -0.597226857  0.438576047 -0.390665284  1.396600194  0.941536561
## [176]  0.543049037  1.193112629  2.007022326 -0.925151208  0.230865803
## [181] -0.419447636 -0.801904042 -0.503408327 -0.077885862 -0.042571098
## [186] -1.013776597 -0.894168974 -0.798153030 -0.204205632 -0.686647038
## [191] -1.406394909 -1.297308217 -1.152307700  1.334636129  0.045668550
## [196] -0.961587017  0.716106771 -1.197836394 -0.686142514  2.390841553
## [201] -0.679656064 -1.207756217 -1.143177413 -0.673546426  0.782591054
## [206]  0.210694371  0.401807024  0.735658879 -0.060839769  1.303934705
## [211]  0.490359527 -0.301789014
```

```
plot(simulationOutput)
```

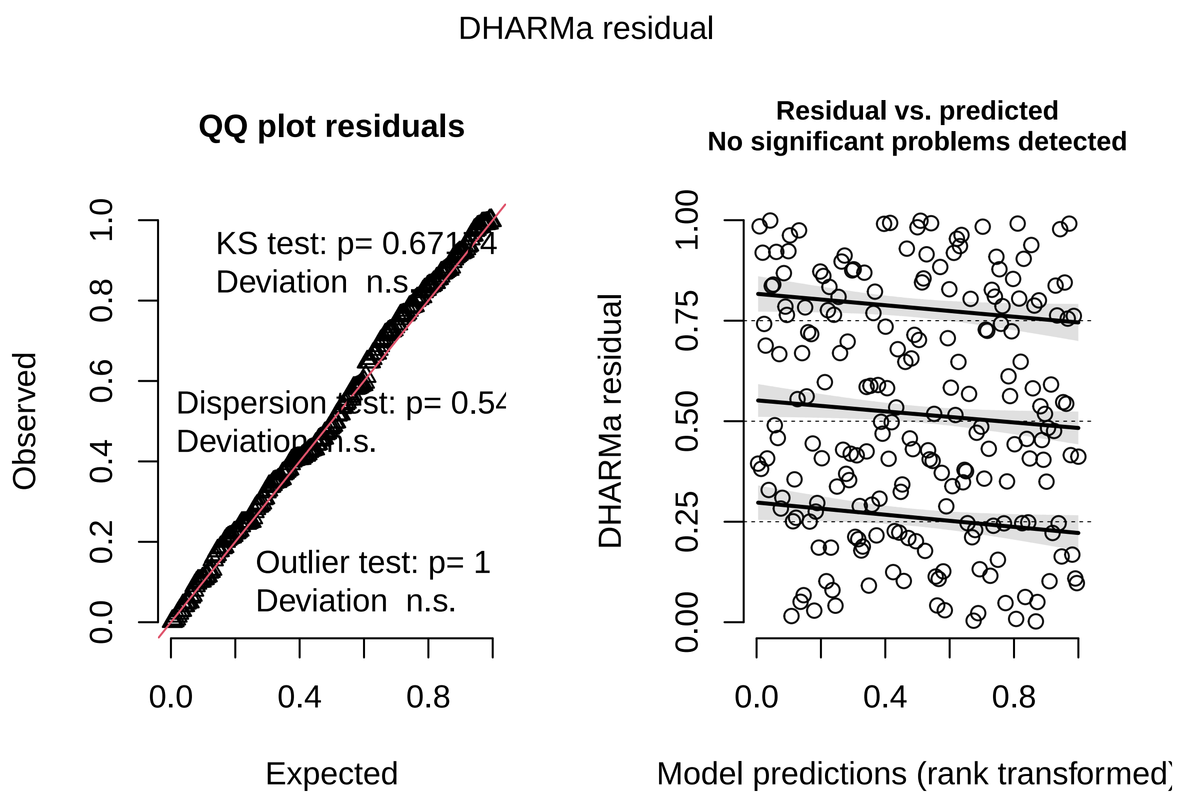


```
testDispersion(m8)
```

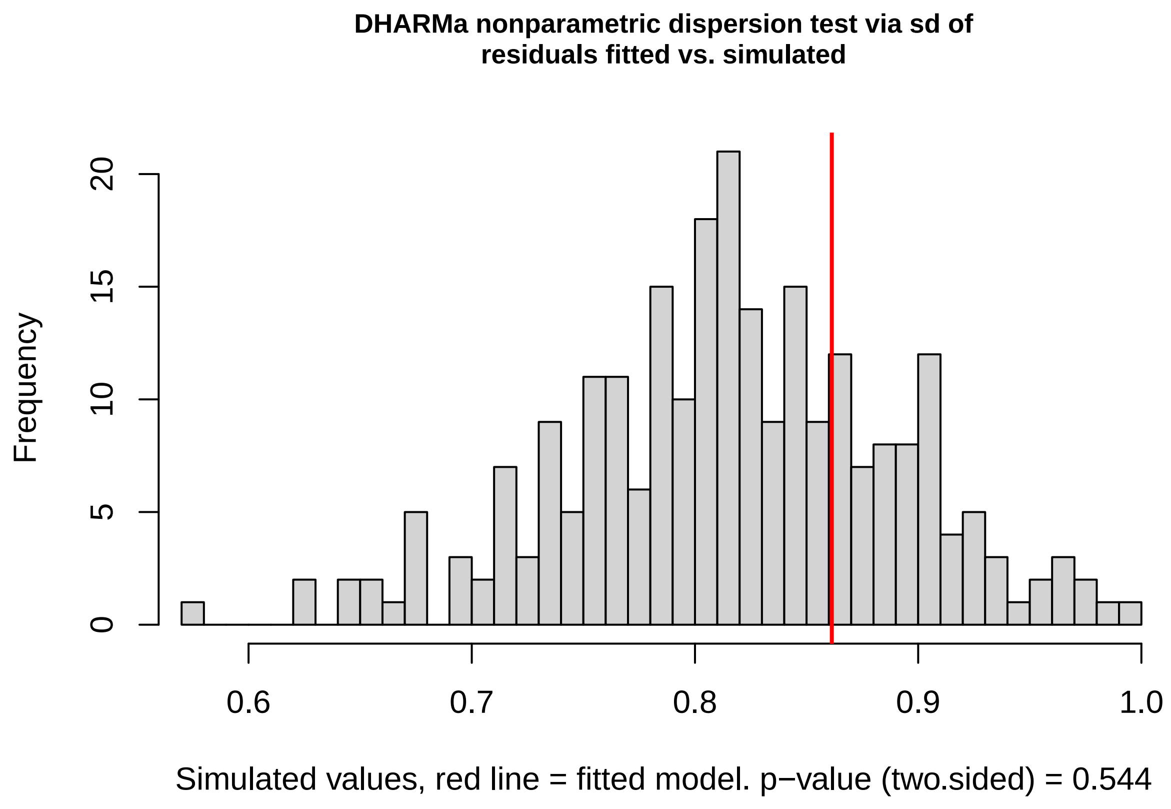


```
##
##  DHARMa nonparametric dispersion test via sd of residuals fitted vs.
##  simulated
##
## data:  simulationOutput
## dispersion = 1.0557, p-value = 0.544
## alternative hypothesis: two.sided
```

```
simulationOutput <- simulateResiduals(fittedModel = m8, plot = F)
residuals(simulationOutput)
```

```
##   [1] 0.324299843 0.308009110 0.929199181 0.229358871 0.029733374 0.451093504
##   [7] 0.185557748 0.757475801 0.259899367 0.822206700 0.131801435 0.500375868
##  [13] 0.518249592 0.598174052 0.486291943 0.238025387 0.062011321 0.219124933
##  [19] 0.221518114 0.353135436 0.050349727 0.376616098 0.738669662 0.427152470
##  [25] 0.585254410 0.200263326 0.675513053 0.922914992 0.836345783 0.867565600
##  [31] 0.998980683 0.878770629 0.786858347 0.441014213 0.245897881 0.805489606
##  [37] 0.091066864 0.328933330 0.449734134 0.187135085 0.338320782 0.830343463
##  [43] 0.405738872 0.296536104 0.584191686 0.561730992 0.905262678 0.340575322
##  [49] 0.279039610 0.230598760 0.429029149 0.611674922 0.698690374 0.403693736
##  [55] 0.899189084 0.041075884 0.724898481 0.953696141 0.972493903 0.647474226
##  [61] 0.958794354 0.471263615 0.420508118 0.593380868 0.913875676 0.742274414
##  [67] 0.178343853 0.963241267 0.357199105 0.567892355 0.375557361 0.786216931
##  [73] 0.163516465 0.874939972 0.108344506 0.450541698 0.483363819 0.515410011
##  [79] 0.876114719 0.764739541 0.415102031 0.371110989 0.001989429 0.983709024
```


Biology Open • Supplementary information

```
##  [85] 0.640214316 0.875090412 0.980683495 0.395741511 0.852897301 0.290077565
##  [91] 0.721340935 0.698076696 0.785032397 0.727647755 0.855320302 0.992577400
##  [97] 0.495801708 0.721704327 0.015392466 0.682822063 0.735824065 0.435015057
## [103] 0.457211560 0.832470417 0.938251491 0.394480103 0.540773215 0.803817196
## [109] 0.427627759 0.847575874 0.990963963 0.808379367 0.937823467 0.065946518
## [115] 0.565400179 0.919111348 0.554910020 0.348857472 0.753667119 0.246230661
## [121] 0.248706978 0.998670498 0.537221687 0.041120597 0.991454598 0.103350495
## [127] 0.761816029 0.801817644 0.050291303 0.395391708 0.588018968 0.844951672
## [133] 0.643047412 0.292165472 0.433226911 0.100510904 0.353461279 0.720023503
## [139] 0.845217976 0.982612383 0.207537184 0.407430554 0.666832660 0.295656716
## [145] 0.215921307 0.852167685 0.349641891 0.407797048 0.921366646 0.455948805
## [151] 0.838140433 0.780972825 0.872041274 0.406521260 0.993309329 0.804298685
## [157] 0.103163273 0.373880602 0.022506128 0.534202014 0.714788627 0.045831775
## [163] 0.597094937 0.435118043 0.008274075 0.003749817 0.549882388 0.108774580
## [169] 0.215038096 0.028563293 0.273802075 0.669515622 0.359859826 0.922922100
## [175] 0.824274657 0.720841589 0.883587367 0.977626357 0.183023618 0.586871989
## [181] 0.337444508 0.211304234 0.305453096 0.468959423 0.483021718 0.155344682
## [187] 0.184696855 0.213452800 0.419096405 0.242913740 0.078629866 0.095766232
## [193] 0.127617868 0.907612988 0.518212785 0.168128548 0.760461607 0.122881741
## [199] 0.253775610 0.991595097 0.248361131 0.113570524 0.126482469 0.250299862
## [205] 0.783066352 0.583437116 0.656086974 0.769030840 0.478920722 0.903872077
## [211] 0.688060244 0.381406453
```

```
residuals(simulationOutput, quantileFunction = qnorm, outlierValues = c(-7,7))
```

```
##   [1] -0.4557083924 -0.5015015032  1.4698527562 -0.7409599190 -1.8847266875
##   [6] -0.1228990871 -0.8943858226  0.6982059621 -0.6436556830  0.9238074022
##  [11] -1.1179160023  0.0009421618  0.0457609086  0.2486236723 -0.0343677680
##  [16] -0.7126687236 -1.5381062341 -0.7751519699 -0.7670761737 -0.3768691191
##  [21] -1.6414721044 -0.3143803276  0.6392494384 -0.1836284541  0.2153541934
##  [26] -0.8406810282  0.4551881366  1.4249556705  0.9795497212  1.1149571137
##  [31]  3.0845454796  1.1688632251  0.7955677689 -0.1483983187 -0.6874554559
##  [36]  0.8613945278 -1.3342140166 -0.4428604727 -0.1263330849 -0.8885031342
##  [41] -0.4170503708  0.9555234063 -0.2385200799 -0.5343893192  0.2126286424
##  [46]  0.1553593677  1.3121348338 -0.4108934797 -0.5856968971 -0.7368763958
##  [51] -0.1788464170  0.2836871342  0.5206375988 -0.2437977383  1.2769445305
##  [56] -1.7383351984  0.5974559050  1.6817995433  1.9187798977  0.3785102928
##  [61]  1.7368633601 -0.0720938383 -0.2005937621  0.2362505392  1.3650140031
##  [66]  0.6503732175 -0.9216949695  1.7896048073 -0.3659555970  0.1710107778
##  [71] -0.3171698565  0.7933633822 -0.9801077598  1.1500578216 -1.2353802685
##  [76] -0.1242928656 -0.0417128141  0.0386367810  1.1557814521  0.7216317419
##  [81] -0.2144398591 -0.3289122990 -2.8798330151  2.1371972531  0.3590317081
##  [86]  1.1507886988  2.0680745953 -0.2643853194  1.0489407323 -0.5531581375
##  [91]  0.5868296378  0.5188768715  0.7893025330  0.6057142944  1.0595279758
##  [96]  2.4361336321 -0.0105237518  0.5879120215 -2.1598416288  0.4756049142
## [101]  0.6305239002 -0.1636202347 -0.1074611803  0.9639735900  1.5402598813
## [106] -0.2676610850  0.1023818746  0.8553351964 -0.1824169595  1.0260919323
## [111]  2.3641381216  0.8719397161  1.5367560220 -1.5066786937  0.1646751948
## [116]  1.3991189921  0.1380764979 -0.3884068930  0.6860750840 -0.6863993390
## [121] -0.6785643196  3.0046343177  0.0934367120 -1.7378276154  2.3847484317
## [126] -1.2626889416  0.7121563832  0.8481315520 -1.6420357038 -0.2652934422
## [131]  0.2224519644  1.0150192389  0.3666163961 -0.5470695602 -0.1681645597
## [136] -1.2786458184 -0.3759923849  0.5829113291  1.0161372187  2.1109660101
## [141] -0.8149964037 -0.2341595603  0.4311838714 -0.5369336694 -0.7860424586
```


Biology Open • Supplementary information

```
## [146]  1.0457756411 -0.3862874657 -0.2332154672  1.4143242903 -0.1106453164
## [151]  0.9868439734  0.7754829269  1.1360934580 -0.2365028199  2.4734556192
## [156]  0.8570764586 -1.2637311352 -0.3215927936 -2.0045399210  0.0858370261
## [161]  0.5674290071 -1.6866870111  0.2458347877 -0.1633586146 -2.3965961932
## [166] -2.6738036731  0.1253642069 -1.2330713123 -0.7890612778 -1.9023410198
## [171] -0.6013541187  0.4385760470 -0.3588334978  1.4250048497  0.9317791221
## [176]  0.5853434239  1.1931126289  2.0070223264 -0.9039022497  0.2195058528
## [181] -0.4194476356 -0.8019040415 -0.5087803523 -0.0778858625 -0.0425710979
## [186] -1.0137765970 -0.8976096170 -0.7944979544 -0.2042056321 -0.6969605548
## [191] -1.4143480641 -1.3060590902 -1.1377240293  1.3261982945  0.0456685503
## [196] -0.9615870169  0.7077882137 -1.1607010818 -0.6626554926  2.3908415535
## [201] -0.6796560638 -1.2077562172 -1.1431774125 -0.6735464265  0.7825910537
## [206]  0.2106943711  0.4018070239  0.7356588795 -0.0528625251  1.3039347050
## [211]  0.4903595270 -0.3017890144
```

```
plot(simulationOutput)
```

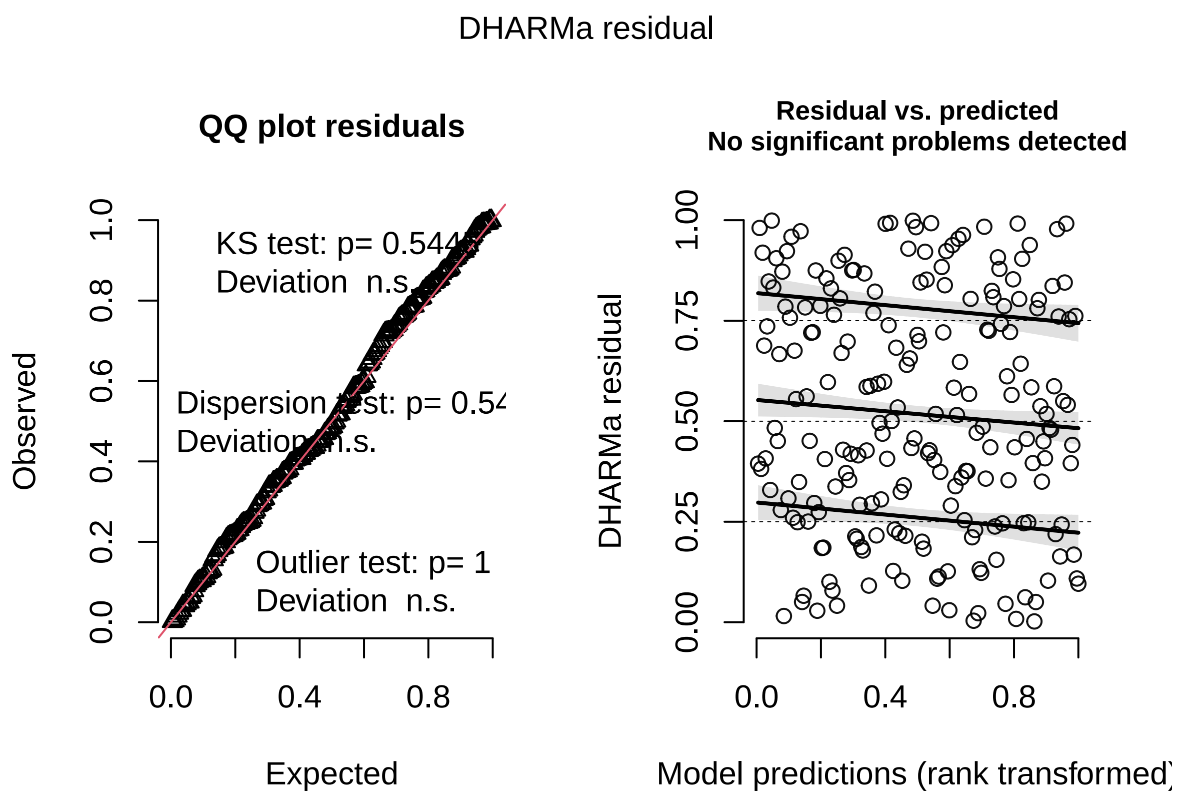

Analysis of effects of body weight gain during treatment on pupation

```
# NULL MODEL
m0h <- glmmTMB(
  pupated ~ 1 + (1|colony),
  family = "binomial", Df)
```


Biology Open • Supplementary information

```
# FULL MODEL
m9 <- glmmTMB(
  pupated ~ treatment + rel_BWG_treatment + (1|colony),
  family = "binomial", Df)
summary(m9)
```

```
##  Family: binomial  ( logit )
## Formula:          pupated ~ treatment + rel_BWG_treatment + (1 | colony)
## Data: Df
##
##      AIC      BIC   logLik deviance df.resid
##    186.0    202.8    -88.0    176.0      207
##
## Random effects:
##
## Conditional model:
##  Groups Name        Variance Std.Dev.
##  colony (Intercept) 0.06051  0.246
## Number of obs: 212, groups:  colony, 5
##
## Conditional model:
##                    Estimate Std. Error z value Pr(>|z|)
## (Intercept)        2.295980   0.412733   5.563 2.65e-08 ***
## treatment37°C     -0.263276   0.480133  -0.548    0.583
## treatment38°C     -0.818014   0.504900  -1.620    0.105
## rel_BWG_treatment -0.038333   0.009837  -3.897 9.75e-05 ***
## ---
## Signif. codes:  0 '***' 0.001 '**' 0.01 '*' 0.05 '.' 0.1 ' ' 1
```

```
Anova(m9)
```

```
## Analysis of Deviance Table (Type II Wald chisquare tests)
##
## Response: pupated
##                     Chisq Df Pr(>Chisq)
## treatment          2.7504  2     0.2528
## rel_BWG_treatment 15.1848  1  9.748e-05 ***
## ---
## Signif. codes:  0 '***' 0.001 '**' 0.01 '*' 0.05 '.' 0.1 ' ' 1
```

```
m10 <- glmmTMB(
  pupated ~ treatment * rel_BWG_treatment + (1|colony),
  family = "binomial", Df)
summary(m10)
```

```
##  Family: binomial  ( logit )
## Formula:          pupated ~ treatment * rel_BWG_treatment + (1 | colony)
## Data: Df
##
##      AIC      BIC   logLik deviance df.resid
##    187.9    211.4    -86.9    173.9      205
##
```

```
## Random effects:
##
## Conditional model:
##  Groups Name        Variance Std.Dev.
##  colony (Intercept) 0.05272  0.2296
## Number of obs: 212, groups:  colony, 5
##
## Conditional model:
##                                 Estimate Std. Error z value Pr(>|z|)
## (Intercept)                      2.426090   0.507703   4.779 1.77e-06 ***
## treatment37°C                   -0.157761   0.684633  -0.230  0.81776
## treatment38°C                   -1.031032   0.595932  -1.730  0.08361 .
## rel_BWG_treatment               -0.044155   0.015861  -2.784  0.00537 **
## treatment37°C:rel_BWG_treatment -0.008209   0.024203  -0.339  0.73449
## treatment38°C:rel_BWG_treatment  0.022027   0.020828   1.058  0.29025
## ---
## Signif. codes:  0 '***' 0.001 '**' 0.01 '*' 0.05 '.' 0.1 ' ' 1
```

```
Anova(m10)
```

```
## Analysis of Deviance Table (Type II Wald chisquare tests)
##
## Response: pupated
##                             Chisq Df Pr(>Chisq)
## treatment                  2.5560  2  0.2785999
## rel_BWG_treatment         14.5684  1  0.0001352 ***
## treatment:rel_BWG_treatment  2.1576  2  0.3399966
## ---
## Signif. codes:  0 '***' 0.001 '**' 0.01 '*' 0.05 '.' 0.1 ' ' 1
```

```
m11<- glmmTMB(
  pupated ~ rel_BWG_treatment + (1|colony),
  family = "binomial", Df)
summary(m11)
```

```
##  Family: binomial  ( logit )
## Formula:          pupated ~ rel_BWG_treatment + (1 | colony)
## Data: Df
##
##      AIC      BIC   logLik deviance df.resid
##    184.7    194.8    -89.4    178.7      209
##
## Random effects:
##
## Conditional model:
##  Groups Name        Variance Std.Dev.
##  colony (Intercept) 0.02032  0.1425
## Number of obs: 212, groups:  colony, 5
##
## Conditional model:
##                   Estimate Std. Error z value Pr(>|z|)
## (Intercept)        1.906350   0.236263   8.069  7.1e-16 ***
## rel_BWG_treatment -0.033840   0.009012  -3.755 0.000173 ***
```


Biology Open • Supplementary information

```
## ---
## Signif. codes:  0 '***' 0.001 '**' 0.01 '*' 0.05 '.' 0.1 ' ' 1
```

```
Anova(m11)
```

```
## Analysis of Deviance Table (Type II Wald chisquare tests)
##
## Response: pupated
##                    Chisq Df Pr(>Chisq)
## rel_BWG_treatment 14.101  1  0.0001732 ***
## ---
## Signif. codes:  0 '***' 0.001 '**' 0.01 '*' 0.05 '.' 0.1 ' ' 1
```

```
### Post-hoc tests
### Comparing slopes
em_means9 <- emmeans(m9, ~ treatment | rel_BWG_treatment) # Obtain marginal means
em_means9
```

```
## rel_BWG_treatment = 5.47:
##  treatment emmean    SE  df asymp.LCL asymp.UCL
##  Ctrl        2.09 0.388 Inf     1.326      2.85
##  37°C        1.82 0.362 Inf     1.114      2.53
##  38°C        1.27 0.354 Inf     0.575      1.96
##
## Results are given on the logit (not the response) scale.
## Confidence level used: 0.95
```

```
em_means10 <- emmeans(m10, ~ treatment | rel_BWG_treatment) # Obtain marginal means
em_means10
```

```
## rel_BWG_treatment = 5.47:
##  treatment emmean    SE  df asymp.LCL asymp.UCL
##  Ctrl        2.18 0.450 Inf     1.302      3.07
##  37°C        1.98 0.424 Inf     1.151      2.81
##  38°C        1.27 0.339 Inf     0.609      1.94
##
## Results are given on the logit (not the response) scale.
## Confidence level used: 0.95
```

```
em_means11 <- emmeans(m11, ~ rel_BWG_treatment) # Obtain marginal means
em_means11
```

```
##  rel_BWG_treatment emmean    SE  df asymp.LCL asymp.UCL
##               5.47   1.72 0.215 Inf       1.3      2.14
##
## Results are given on the logit (not the response) scale.
## Confidence level used: 0.95
```

```
# Lower AIC or BIC values indicate better-fitting models
AIC(m0h, m9, m10, m11)
```


Biology Open • Supplementary information

```
##     df      AIC
## m0h  2 200.3096
## m9   5 185.9677
## m10  7 187.8631
## m11  3 184.7403
```

```
BIC(m0h, m9, m10, m11)
```

```
##     df      BIC
## m0h  2 207.0227
## m9   5 202.7506
## m10  7 211.3592
## m11  3 194.8101
```

```
lrtest(m9, m11)
```

```
## Likelihood ratio test
##
## Model 1: pupated ~ treatment + rel_BWG_treatment + (1 | colony)
## Model 2: pupated ~ rel_BWG_treatment + (1 | colony)
##   #Df  LogLik Df  Chisq Pr(>Chisq)
## 1   5 -87.984
## 2   3 -89.370 -2 2.7726       0.25
```

```
# Compared to NULL model
lrtest(m0h, m11)
```

```
## Likelihood ratio test
##
## Model 1: pupated ~ 1 + (1 | colony)
## Model 2: pupated ~ rel_BWG_treatment + (1 | colony)
##   #Df  LogLik Df  Chisq Pr(>Chisq)
## 1   2 -98.155
## 2   3 -89.370  1 17.569   2.77e-05 ***
## ---
## Signif. codes:  0 '***' 0.001 '**' 0.01 '*' 0.05 '.' 0.1 ' ' 1
```

```
anova(m0h, m11)
```

```
## Data: Df
## Models:
## m0h: pupated ~ 1 + (1 | colony), zi=~0, disp=~1
## m11: pupated ~ rel_BWG_treatment + (1 | colony), zi=~0, disp=~1
##     Df    AIC    BIC  logLik deviance  Chisq Chi Df Pr(>Chisq)
## m0h  2 200.31 207.02 -98.155   196.31
## m11  3 184.74 194.81 -89.370   178.74 17.569      1   2.77e-05 ***
## ---
## Signif. codes:  0 '***' 0.001 '**' 0.01 '*' 0.05 '.' 0.1 ' ' 1
```


Biology Open • Supplementary information

```r
# Model diagnostics
testDispersion(m11)
```

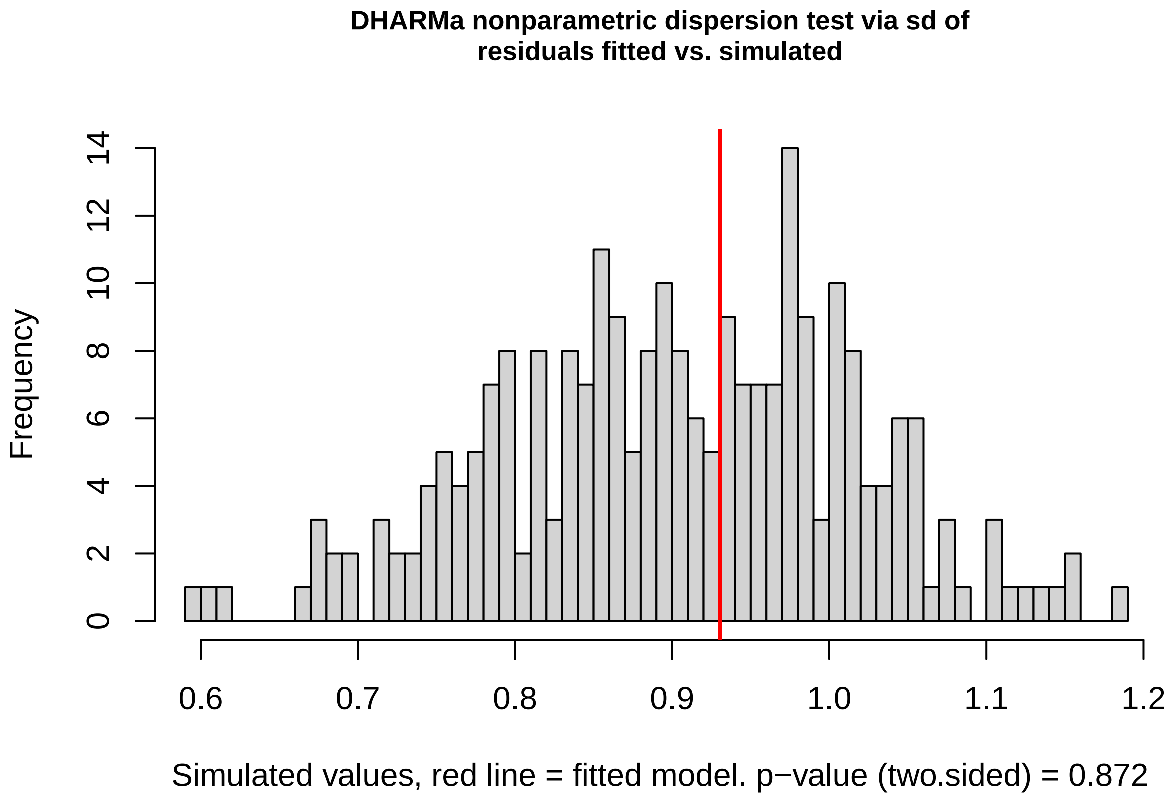


```
##
##  DHARMa nonparametric dispersion test via sd of residuals fitted vs.
##  simulated
##
## data:  simulationOutput
## dispersion = 1.0306, p-value = 0.872
## alternative hypothesis: two.sided
```

```r
simulationOutput <- simulateResiduals(fittedModel = m11, plot = F)
residuals(simulationOutput)
```

```
##   [1] 0.57132483 0.07664076 0.85053161 0.52598465 0.00814613 0.70674344
##   [7] 0.45174115 0.13772287 0.14288306 0.59823332 0.38766934 0.78837743
##  [13] 0.80872717 0.84790197 0.86105357 0.53488330 0.25717796 0.74090769
##  [19] 0.06906153 0.75557528 0.23553951 0.12388687 0.33961117 0.05511645
##  [25] 0.06938583 0.46083628 0.25215003 0.60080978 0.78815871 0.66707463
##  [31] 0.99102030 0.80776485 0.92429065 0.19402049 0.67477659 0.19577433
##  [37] 0.01278131 0.51773213 0.24593196 0.05686425 0.61794834 0.17384643
##  [43] 0.58543383 0.09542003 0.43586240 0.70700546 0.99423782 0.10447096
##  [49] 0.44638822 0.46071851 0.73707699 0.40953310 0.31951422 0.69427877
##  [55] 0.61938736 0.13223276 0.46427599 0.90386437 0.78947256 0.32642397
```


Biology Open • Supplementary information

```
##  [61] 0.78138116 0.82052700 0.66399329 0.87618199 0.65550270 0.56725181
##  [67] 0.32717574 0.91448758 0.68173196 0.17939190 0.67512785 0.66841810
##  [73] 0.02637362 0.53435096 0.22321128 0.56886013 0.16258601 0.96247750
##  [79] 0.69115923 0.98724382 0.65691728 0.62510621 0.08962444 0.97029293
##  [85] 0.93491670 0.55878710 0.62874466 0.12038321 0.79497561 0.05691395
##  [91] 0.89893462 0.11572394 0.94101644 0.51831917 0.37699151 0.98508057
##  [97] 0.72198428 0.57782357 0.39069880 0.29101402 0.80685522 0.11053046
## [103] 0.77028502 0.94866583 0.91458739 0.87647394 0.39204636 0.71814243
## [109] 0.71093147 0.95528141 0.96963299 0.69395841 0.86380378 0.24962483
## [115] 0.34810027 0.59359275 0.24831590 0.06591316 0.65571018 0.69557665
## [121] 0.05983045 0.99689331 0.35266129 0.16076676 0.98904064 0.39862114
## [127] 0.68983736 0.71618329 0.25155836 0.20393242 0.06603422 0.80266576
## [133] 0.44012231 0.54006184 0.15258306 0.26127339 0.51857257 0.27170698
## [139] 0.53356228 0.96190354 0.05867543 0.74682569 0.82382733 0.44478456
## [145] 0.05742588 0.69238932 0.14270977 0.12845603 0.78822608 0.22524355
## [151] 0.62012551 0.69388973 0.46182065 0.68791250 0.98215821 0.65337648
## [157] 0.21115046 0.60929373 0.18697403 0.78874871 0.41175154 0.19603813
## [163] 0.77447775 0.94572184 0.16511047 0.16550672 0.39387572 0.46489467
## [169] 0.07540297 0.16995662 0.06466682 0.28436416 0.61146473 0.80227843
## [175] 0.72637054 0.32368839 0.78128536 0.96960806 0.30783944 0.47420071
## [181] 0.10513346 0.05282606 0.61727784 0.73381450 0.26369760 0.42011792
## [187] 0.03308003 0.07327484 0.62320551 0.82798007 0.22771538 0.39912337
## [193] 0.25368112 0.83814907 0.35370008 0.76853256 0.69993309 0.27404580
## [199] 0.50491134 0.98834658 0.70087370 0.35421971 0.36752645 0.09654423
## [205] 0.93476006 0.19058946 0.19753627 0.47165805 0.26286346 0.85960264
## [211] 0.40644909 0.80508651
```

```r
residuals(simulationOutput, quantileFunction = qnorm, outlierValues = c(-7,7))
```

```
##   [1]  0.17974809 -1.42803589  1.03871610  0.06517998 -2.40230183  0.54389587
##   [7] -0.12126354 -1.09060723 -1.06745565  0.24877690 -0.28539874  0.80080398
##  [13]  0.87321544  1.02747668  1.08506502  0.08755119 -0.65207016  0.64614629
##  [19] -1.48281692  0.69213995 -0.72072453 -1.15577366 -0.41352454 -1.59714722
##  [25] -1.48038075 -0.09832711 -0.66773922  0.25544368  0.80004870  0.43184959
##  [31]  2.36645416  0.86968917  1.43453833 -0.86317549  0.45314156 -0.85681224
##  [37] -2.23279254  0.04446251 -0.68734726 -1.58165433  0.30009681 -0.93907380
##  [43]  0.21581450 -1.30809802 -0.16146801  0.54465753  2.52638329 -1.25648021
##  [49] -0.13479186 -0.09862374  0.63435981 -0.22874618 -0.46905765  0.50801552
##  [55]  0.30387217 -1.11589866 -0.08966683  1.30388950  0.80459249 -0.44980933
##  [61]  0.77686624  0.91737530  0.42338633  1.15611038  0.40021985  0.16938176
##  [67] -0.44772529  1.36891822  0.47254744 -0.91768500  0.45411743  0.43554924
##  [73] -1.93698443  0.08621176 -0.76139269  0.17347289 -0.98388507  1.78018920
##  [79]  0.49913889  2.23355516  0.40406430  0.31891947 -1.34307135  1.88511623
##  [85]  1.51344526  0.14789479  0.32853038 -1.17307327  0.82380780 -1.58121929
##  [91]  1.27550440 -1.19663747  1.56336350  0.04593550 -0.31339180  2.17222295
##  [97]  0.58874635  0.19632875 -0.27749819 -0.55042479  0.86636588 -1.22371191
## [103]  0.73978584  1.63205289  1.36955704  1.15753924 -0.27398947  0.57733208
## [109]  0.55610795  1.69837411  1.87542690  0.50710211  1.09756970 -0.67567084
## [115] -0.39045444  0.23679670 -0.67979891 -1.50693887  0.40078336  0.51172041
## [121] -1.55619847  2.73630265 -0.37814541 -0.99131147  2.29177353 -0.25691774
## [127]  0.49538938  0.57154035 -0.66959386 -0.82765691 -1.50599510  0.85118162
## [133] -0.15065912  0.10058951 -1.02541773 -0.63942455  0.04657135 -0.60765854
## [139]  0.08422764  1.77321597 -1.56599038  0.66453396  0.93004973 -0.13884946
## [145] -1.57675566  0.50263438 -1.06822389 -1.13371989  0.80028129 -0.75460320
```


Biology Open • Supplementary information

```
## [151]  0.30581043  0.50690634 -0.09584799  0.48994191  2.10051481  0.39445244
## [157] -0.80243578  0.27747872 -0.88910240  0.80208709 -0.22304166 -0.85585812
## [163]  0.75367484  1.60471599 -0.97366897 -0.97207458 -0.26923164 -0.08810989
## [169] -1.43669056 -0.95433668 -1.51673484 -0.56992536  0.28313866  0.84978769
## [175]  0.60187263 -0.45740945  0.77654157  1.87506425 -0.50198385 -0.06471436
## [181] -1.25283179 -1.61804863  0.29833916  0.62439073 -0.63198727 -0.20159183
## [187] -1.83733763 -1.45182711  0.31391053  0.94621319 -0.74639182 -0.25561681
## [193] -0.66295052  0.98687920 -0.37535004  0.73402272  0.52420808 -0.60062228
## [199]  0.01231120  2.26836701  0.52691504 -0.37395282 -0.33841178 -1.30149678
## [205]  1.51221224 -0.87572617 -0.85045432 -0.07110261 -0.63454238  1.07853578
## [211] -0.23668886  0.85993117
```

```
plot(simulationOutput)
```

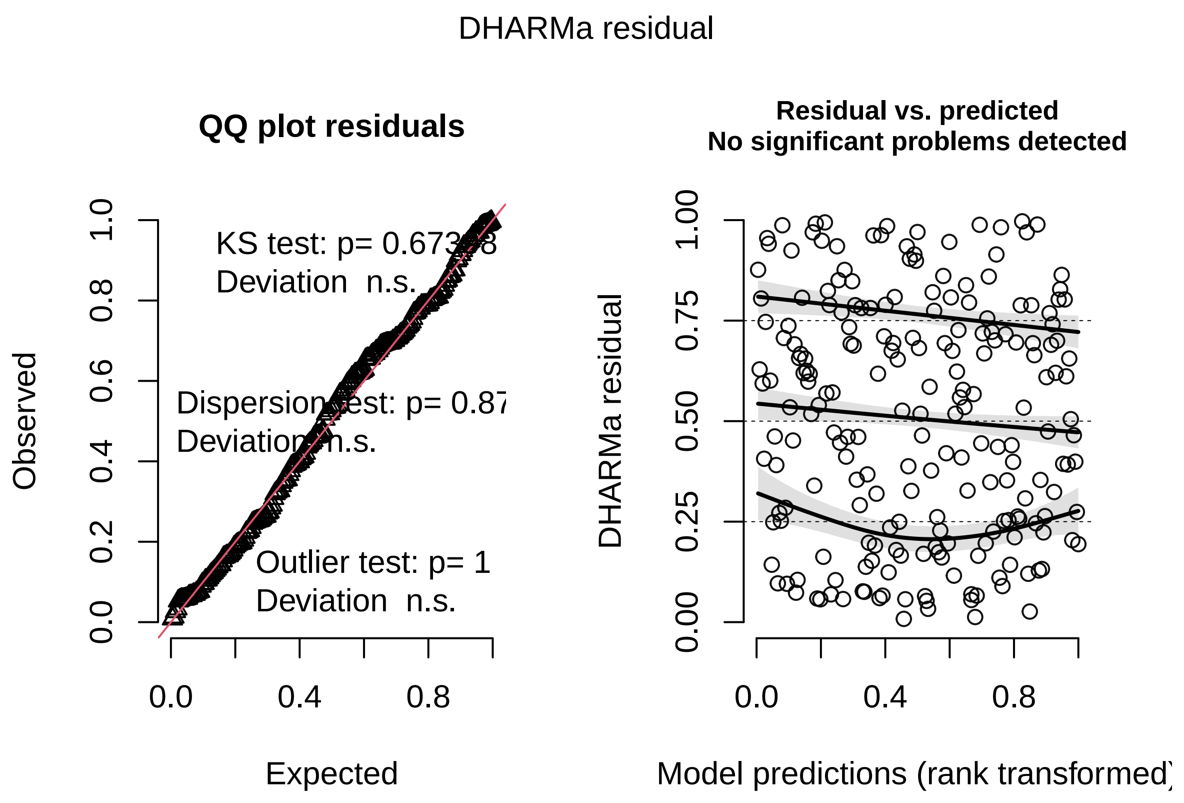

Session info

```
sessionInfo()
```

```
## R version 4.4.2 (2024-10-31)
## Platform: aarch64-apple-darwin20
## Running under: macOS Sequoia 15.3.1
##
## Matrix products: default
```


Biology Open • Supplementary information

```
## BLAS:   /Library/Frameworks/R.framework/Versions/4.4-arm64/Resources/lib/libRblas.0.dylib
## LAPACK: /Library/Frameworks/R.framework/Versions/4.4-arm64/Resources/lib/libRlapack.dylib;  LAPACK version 3.12.0
##
## locale:
## [1] en_US.UTF-8/en_US.UTF-8/en_US.UTF-8/C/en_US.UTF-8/en_US.UTF-8
##
## time zone: Europe/Berlin
## tzcode source: internal
##
## attached base packages:
## [1] stats     graphics  grDevices utils     datasets  methods   base
##
## other attached packages:
##  [1] lmtest_0.9-40   zoo_1.8-12      car_3.1-3       carData_3.0-5
##  [5] lme4_1.1-35.5   Matrix_1.7-1    DHARMa_0.4.6    glmmTMB_1.1.10
##  [9] emmeans_1.10.4  gridExtra_2.3   ggforce_0.4.2   ggsignif_0.6.4
## [13] lubridate_1.9.3 forcats_1.0.0   stringr_1.5.1   dplyr_1.1.4
## [17] purrr_1.0.2     tidyr_1.3.1     tibble_3.2.1    ggplot2_3.5.1
## [21] tidyverse_2.0.0 readr_2.1.5     Rmisc_1.5.1     plyr_1.8.9
## [25] lattice_0.22-6
##
## loaded via a namespace (and not attached):
##  [1] tidyselect_1.2.1    farver_2.1.2        fastmap_1.2.0
##  [4] TH.data_1.1-2       tweenr_2.0.3        promises_1.3.0
##  [7] digest_0.6.37       mime_0.12           timechange_0.3.0
## [10] estimability_1.5.1  lifecycle_1.0.4     survival_3.7-0
## [13] magrittr_2.0.3      compiler_4.4.2      rlang_1.1.5
## [16] tools_4.4.2         utf8_1.2.4          yaml_2.3.10
## [19] knitr_1.49          labeling_0.4.3      bit_4.5.0
## [22] gap.datasets_0.0.6  multcomp_1.4-26     abind_1.4-8
## [25] withr_3.0.2         numDeriv_2016.8-1.1 grid_4.4.2
## [28] polyclip_1.10-7     xtable_1.8-4        colorspace_2.1-1
## [31] iterators_1.0.14    scales_1.3.0        MASS_7.3-61
## [34] cli_3.6.3           mvtnorm_1.3-1       rmarkdown_2.29
## [37] crayon_1.5.3        ragg_1.3.3          reformulas_0.3.0
## [40] generics_0.1.3      rstudioapi_0.16.0   tzdb_0.4.0
## [43] minqa_1.2.8         splines_4.4.2       parallel_4.4.2
## [46] vctrs_0.6.5         boot_1.3-31         sandwich_3.1-1
## [49] hms_1.1.3           bit64_4.5.2         qgam_1.3.4
## [52] Formula_1.2-5       systemfonts_1.1.0   foreach_1.5.2
## [55] gap_1.6             glue_1.8.0          nloptr_2.1.1
## [58] codetools_0.2-20    stringi_1.8.4       gtable_0.3.6
## [61] later_1.3.2         munsell_0.5.1       pillar_1.10.1
## [64] htmltools_0.5.8.1   R6_2.5.1            TMB_1.9.15
## [67] textshaping_0.4.0   Rdpack_2.6.1        doParallel_1.0.17
## [70] shiny_1.9.1         vroom_1.6.5         evaluate_1.0.3
## [73] rbibutils_2.2.16    httpuv_1.6.15       Rcpp_1.0.14
## [76] nlme_3.1-166        mgcv_1.9-1          xfun_0.50
## [79] pkgconfig_2.0.3
```


Biology Open • Supplementary information
